# Field screening and genetic mapping of wheat blast resistance for a panel of common wheat from Bangladesh

**DOI:** 10.1371/journal.pone.0349201

**Published:** 2026-06-11

**Authors:** Md. Farhad, Xinyao He, Hirokazu Handa, Muhammad Rezaul Kabir, Krishna Kanta Roy, Felix Marza, Masahiro Kishi, Soichiro Asuke, Pawan K. Singh

**Affiliations:** 1 Bangladesh Wheat and Maize Research Institute (BWMRI), Nashipur, Dinajpur, Bangladesh; 2 International Maize and Wheat Improvement Center (CIMMYT), Mexico DF, Mexico; 3 Graduate School of Life and Environmental Sciences, Kyoto Prefectural University, Kyoto, Japan; 4 Instituto Nacional de Innovación Agropecuaria y Forestal (INIAF), La Paz, Bolivia; 5 Japan International Research Center for Agricultural Sciences, Tsukuba, Japan; 6 Graduate School of Agricultural Science, Kobe University Kobe, Japan; International Center for Agricultural Research in the Dry Areas, MOROCCO

## Abstract

Wheat blast, caused by *Magnaporthe oryzae* pathotype *Triticum* (MoT), is a rapidly emerging disease threatening wheat production in South Asia and beyond. To identify novel sources of resistance and dissect the underlying genetic architecture, a genome-wide association study (GWAS) was conducted using 432 diverse Bangladeshi wheat genotypes phenotyped across 14 multi-environment trials. Disease severity varied widely, with most genotypes exhibiting high susceptibility under favourable environments, although a few showed consistent resistance across locations. Genotyping of the wheat panel using ddRAD-seq revealed moderate population structure and diverse resistance allele distribution. GWAS using six different models identified a total of 1,121 significant SNPs distributed across multiple chromosomes. The 2NS translocation and known resistance genes (*Rmg8*, *Rwt3*, *Rwt4-1B*, *Rwt4-1D*) were unevenly distributed across the panel. Based on linkage disequilibrium (LD), the significant markers were grouped into 45 quantitative trait loci (QTL) associated with wheat blast resistance, including the 2NS/2AS translocation. These QTLs either coincide with known blast resistance loci or represent potentially novel ones. These findings enhance the understanding of wheat blast resistance and provide valuable markers and candidate genotypes for resistance breeding aimed to mitigate the threat of wheat blast under changing climates.

## Introduction

Wheat is the second most important cereal crop in Bangladesh, playing a critical role in national food security and dietary diversification. Over the past decade, wheat consumption in the country has increased rapidly, reaching approximately 7 million metric tons (MMT) annually, whereas domestic production remains around 1.2 MMT [1]. This production gap underscores the strategic importance of strengthening local wheat productivity and resilience. However, wheat cultivation in Bangladesh faces a major threat from wheat blast (WB), a devastating fungal disease that emerged in the country in 2016 [2].

Wheat blast is caused by *Magnaporthe oryzae* pathotype *Triticum* (MoT) and affects all aerial parts of the wheat plant, with bleached spikes being the most conspicuous and yield-limiting symptom, causing losses of up to 100% under severe infection [3]. First reported in Brazil in 1985, the disease remained confined to South America until its sudden outbreak in Bangladesh in 2016 [4]. In 2018, WB was reported in Zambia, marking its spread to Africa [5]. The intercontinental dissemination of WB is largely attributed to its seed-borne nature and global wheat trade [6,7], emphasizing the importance of quarantine and surveillance systems. Predictive models indicate that tropical and subtropical wheat-growing regions—including parts of India, Ethiopia, China, Pakistan, USA, and Australia—are at moderate to high risk [7], and climate change may further expand vulnerable zones.

WB develops rapidly, often destroying spikes within days after symptom onset, making curative control ineffective. Therefore, prevention through resistant cultivars remains central to disease management. Although no complete immunity has been identified in common wheat or its relatives, varietal resistance is an indispensable and farmer-friendly strategy. Early germplasm screening efforts in South America identified resistant sources; however, many of these were later rendered susceptible due to the rapid evolution of virulent MoT isolates [6].

Currently, the most widely deployed resistance source is the 2NS chromosomal segment introgressed from *Aegilops ventricosa* via the French line VPM1 [8]. This alien segment replaces the distal region of chromosome 2AS and confers substantial protection, reducing blast severity by 50.4–80.5% across diverse germplasm [9]. Nevertheless, virulent MoT isolates capable of overcoming 2NS resistance have emerged in South America [6,10], highlighting the vulnerability of relying on a single major resistance source and the urgent need to diversify resistance.

Resistance to WB can be qualitative or quantitative. Qualitative resistance has primarily been observed at the seedling stage, with several major genes identified, including *Rmg2*, *Rmg3*, *Rmg7*, *Rmg8*, *Rmg10*, and *Rmg11*. Among these, *Rmg2*, *Rmg3*, and *Rmg7* have been overcome by new MoT isolates; *Rmg8* showed promising resistance under greenhouse conditions but require large-scale field validation [11]; and *Rmg10* and *Rmg11* exhibit stronger resistance at the seedling stage than at heading [12]. In addition, non-host resistance genes *Rwt3* and *Rwt4* recognize pathogen effectors *PWT3* and *PWT4*, respectively, conferring incompatibility responses [13,14].

Considerable efforts have been made to dissect quantitative resistance through bi-parental mapping and genome-wide association studies (GWAS). In most studies, the 2NS locus remains the only consistently significant major-effect region, whereas non-2NS loci often exhibit minor and environment-dependent effects [15–24]. He *et al*. [15] demonstrated the predominant role of 2NS along with minor QTLs on chromosomes 1AS, 2BL, 3AL, 4BS, 4DL, and 7BS. Subsequent GWAS in South Asian germplasm reinforced the stable effect of 2NS while identifying additional MTAs on 1BS, 2AS, 6BS, and 7BL. Roy *et al.* [20] detected 40 significant markers, of which 82.5% mapped to the 2NS region. *Juliana et al.* [17] confirmed the strong 2NS effect in CIMMYT breeding lines and identified additional MTAs on 3BL, 4AL, and 7BL. Wu *et al.* [23] further refined the 2NS interval, identifying 58 SNPs within a 28.9-Mb region explaining up to 28.5% of phenotypic variation. Beyond 2NS, Goddard *et al.*[25] identified multiple QTL from non-2NS sources in controlled experiments, though field validation remains necessary. More recently, a major QTL, *Qwb.cim-7D*, derived from *Aegilops tauschii* (KU-2097), was mapped on chromosome 7D, explaining 7.7–50.6% of phenotypic variation across field experiments [26].

In Bangladesh, the 2016 WB outbreak resulted in severe yield losses in affected districts, prompting temporary suspension of wheat cultivation and promotion of alternative crops such as boro rice, maize, and gram [7]. The subsequent release of resistant cultivars BARI Gom 33, BWMRI Gom 3, and BWMRI Gom 5, all carrying the 2NS/2AS translocation, enabled partial recovery of wheat production in epidemic regions [7,27]. However, given the rapid evolution of MoT and evidence of 2NS breakdown elsewhere [6,10], reliance on a single resistance source poses substantial risk. Although fungicides and timely sowing can reduce disease pressure [15,28], chemical control may be inconsistent and can promote fungicide resistance when overused [29]. Therefore, identification of additional and potentially complementary resistance loci remains a high priority for sustainable wheat production in Bangladesh and neighbouring regions.

Given the continued threat of wheat blast in Bangladesh and beyond, the erosion risk of 2NS-based resistance, and the need to diversify the genetic basis of resistance, systematic evaluation of locally adapted germplasm under field conditions is essential. Integrating robust phenotyping with genome-wide association approaches enables the identification of both major and minor resistance loci that may contribute to durable resistance under diverse epidemic scenarios. Accordingly, the aims of the present study were: (1) to evaluate a diverse panel of Bangladeshi wheat genotypes for wheat blast resistance under multi-environment field conditions, and (2) to identify molecular markers and associated chromosomal regions (QTL) linked to wheat blast resistance through genome-wide association analysis and linkage disequilibrium (LD)-based locus delineation. These findings are intended to provide a genomic foundation for resistance breeding and future validation studies in Bangladesh and similar agro-ecological regions.

## Materials and methods

### Plant materials

A diverse panel of 432 common wheat (*Triticum aestivum* L.) genotypes with spring growth habit was used in this study. The panel included a representative collection of germplasm cultivated or evaluated in Bangladesh, including a wide range of agro-morphologically and genetically diverse accessions along with synthetic derivatives. These are made up of officially released varieties, advanced breeding lines under national and international trials, and their respective parental lines. A significant portion of the panel included breeding lines developed by the International Maize and Wheat Improvement Centre (CIMMYT), which have been widely utilized in South Asia for their high yield potential and resistance to biotic and abiotic stresses. The panel also incorporated elite cultivars and breeding lines sourced from India and China, selected based on their regional importance, agronomic adaptation, and relevance to wheat blast resistance breeding efforts.

### Field experiments

The field experiments were conducted across three locations: Quirusillas (Quir), and Okinawa (Okin) in Bolivia, and Jashore (Jash) in Bangladesh. The cropping season spans from December to April in both Quirusillas and Jashore, and from May to September in Okinawa. Field trials were conducted during the 2019–20 and 2020–21 cropping seasons in Quirusillas, the 2020 and 2021 seasons in Okinawa, and the 2018–19, 2019–20, and 2020–21 seasons in Jashore. Each trial included two sowing dates approximately 10 days apart, resulting in a total of 14 experiments. The experiments are designated using a naming style that includes the location, year of disease evaluation, and sowing time (with ‘a’ for the first sowing and ‘b’ for the second). For example, Okin21b refers to the second sowing in the 2021 trial conducted at Okinawa.

The plants were sown in 1m double rows separated by 20 cm spacing in all three locations, and no replication was arranged within each sowing. To ensure a favourable micro-environment for wheat blast development, mist-irrigation systems were installed at each site and operated daily from 8:00 AM to 7:00 PM, providing 10 minutes of misting per hour during the disease development period. Field inoculations were conducted twice in each experiment using a backpack sprayer—once at anthesis and again two days later. A mixture of locally collected MoT isolates with high virulence was used as inoculum, involving isolates QUI1505, QUI1601, QUI1612, OKI1503 and OKI1704 in Quirusillas and Okinawa, and BHO17001, MEH17003, GOP17001.2, RAJ17001, CHU16001.3 and JES16001 in Jashore. Spore production was conducted on oatmeal agar medium following the protocol by [15], and the inoculum solution was adjusted to a concentration of 80,000 spores/mL for field application. Local checks used in the experiments were Urubo (resistant check) and Atlax (susceptible check) in Bolivia and BARI Gom 33 (resistant check) and BARI Gom 26 (susceptible check) in Bangladesh.

For WB evaluation, 10 spikes per plot were marked with coloured scotch tape at anthesis and evaluated at 21 days after anthesis [17,19,23], for which the total and infected number of spikelet were counted to derive incidence (the proportion of spikes with blast infection) and severity (the proportion of spikelets damaged in infected spikes). WB index was calculated with the formula WB index = incidence × severity and was used in all subsequent analysis. Days to heading (HD) and plant height in cm (PHT) were recorded for all genotypes alongside the WB index during field evaluations to account for potential phenological escape effects and to support interpretation of disease response independent of maturity differences. Field phenotyping data were curated to retain a biologically consistent and quality-controlled dataset for the study.

The WB index (%), capturing the spectrum of wheat blast responses across multiple environments and epidemic conditions, was used as input for subsequent genome-wide association studies (GWAS) to identify resistance-associated loci and for downstream QTL mapping. To reduce environment-specific noise, GWAS analyses were based on phenotypic information aggregated across environments, rather than on single-environment observations, consistent with common practice in large-scale disease resistance GWAS.

Within-environment analysis of variance (ANOVA) was performed using the Metan v1.19.0 package in R [30] to summarize disease variation among genotypes. In each location-season combination, different planting times were considered as independent temporal evaluations, reflecting variation in disease pressure under artificial inoculation. Although plot-level replication within environments was not implemented, the evaluation across multiple locations, seasons, and planting times provided environmental replication, enabling assessment of genotype responses under diverse disease pressure, as commonly applied in wheat blast screening and GWAS studies [17]. Heritability of means ($\overset{\text{2}}{\underset{gm}{H}}$) was calculated on the mean basis using the Metan v1.19.0 package in R, estimated by


$$\begin{array}{c} \overset{\text{2}}{\underset{gm}{H}}=\overset{\text{2}}{\underset{g}{\sigma }}/[\overset{\text{2}}{\underset{g}{\sigma }}+\left(\frac{\overset{\text{2}}{\underset{i}{\sigma }}}{e}\right)+\left(\frac{\overset{\text{2}}{\underset{e}{\sigma }}}{eb}\right)] \end{array}$$

Where $\overset{\text{2}}{\underset{g}{\sigma }}$ is the genotypic variance; $\overset{\text{2}}{\underset{i}{\sigma }}$ is the genotype-by-environment interaction variance; and $\overset{\text{2}}{\underset{e}{\sigma }}$ is the residual variance. e and b are the number of environments and temporal replication, respectively.

Hierarchical clustering of environments was performed using Ward’s method (Ward.D2) based on a dissimilarity matrix derived from 1−r, where 𝑟 represents the Pearson correlation coefficient among environments. Correlations were computed using pairwise complete observations to accommodate missing values. This approach groups environments according to similarity in genotype performance patterns.

### Genotyping

All the 432 accessions in this study were genotyped using the double digest restriction-site associated DNA sequencing (ddRAD-seq) method. Genomic DNA was digested with *Bgl*II and *Eco*RI, ligated with Y-shaped adaptors, amplified by PCR with KAPA HiFi HS ReadyMix (Kapabio Systems, UK), and then size-selected with the E-Gel size select (Life technologies, CA, USA). Approximately 450 bp library fragments were retrieved. Further details of the library preparation method were described in a previous study [31]. Sequencing was performed with paired-end 151 bp mode of HiSeqX (Illumina, CA, USA). After quality filtering and cleaning of sequence reads performed by Cutadapt ver. 3.1 [32], we mapped them to the wheat reference genome sequence, IWGSC RefSeq v2.1 [33] by BWA ver. 0.7.17 [34]. Sequence variants were called by Haplotype Caller in GATK ver. 4.1.9.0 [35]. We retained only SNPs and then filtered those for missing data less than 25%, which resulted in 82,948 SNPs. Furthermore, we used BEAGLE v3.3.2 [36] to impute the data based on the available allele frequencies obtained after specifying the haplotype phase for all individuals. After removal of duplicate genotypes, we retained the markers for minor allele frequency greater than 5%, resulting in 7,211 markers for 432 accessions.

To identify the 2NS/2AS translocation, four sequence-tagged site (STS) markers previously reported for this region were employed: Ventriup-LN2 [37], WGGB156 and WGGB159 [38], and cslVrgal3 [39]. For the presence of the *Rmg8* gene [40], we used the functional marker for *Rmg8*, KM171 [41]. Genotyping for *Rwt3*, *Rwt4-1B* and *Rwt4-1D* followed the previously used protocols [13].

### Population structure and phylogenetic tree annotation

The population structure of the wheat genotypes was assessed using SNPs derived from filtered ddRAD-seq data. A highly annotated phylogenetic tree was generated in TASSEL 5.2 [42] with visualization in Archaeopteryx, and a Neighbor-Joining tree was constructed to examine genetic diversity and potential for GWAS marker discovery. To elucidate the distribution patterns of known wheat blast resistance loci, the NJ tree was annotated with five concentric tracks, each representing genotypic calls for a specific resistance source: the 2NS translocation, *Rwt3* (*Rmg6*), *Rwt4-B1* (*Rmg1*), *Rwt4-D1* (*Rmg1*), and *KM200* (*Rmg8*) using online tool Evolview Version-3 [43,44]. Genotypic calls were categorized as homozygous positive (1, blue), heterozygous (0.5, green), homozygous negative (–1, red), or missing/no call (0, light green).

### Genome-wide marker-trait associations

GWAS was performed using ‘R’ package GAPIT 3.4.0 [45–47] using six different models namely GLM -General Linear Model [48], MLM -Mixed Linear Model [49,50], MLMM -Multiple Loci Mixed Model [51], SUPER -Settlement of MLM Under Progressively Exclusive Relationship [52], FarmCPU -Fixed and Random Model Circulating Probability Unification [53] and Blink -Bayesian Information and Linkage-Disequilibrium Iteratively Nested Keyway [54]. The GWAS models implemented in GAPIT incorporate population structure as fixed effects (derived from principal component analysis) and genetic relatedness as random effects through a kinship matrix, thereby controlling for confounding due to population stratification and familial relatedness. Statistical details of each model are provided in S1 File. GWAS analyses were conducted within individual environments as well as across environments to capture marker–trait associations expressed under contrasting disease pressure and environmental conditions, and to account for potential genotype × environment interactions in wheat blast response. This approach was intended to identify loci that are environment-specific as well as those showing consistent effects across environments, rather than to explicitly infer race-specific resistance. Manhattan plots with the -log_10_ *p*-values of the markers were generated using the ‘R’ package CMplot v4.5.1 [55]. The standard Bonferroni corrections at α levels of 0.05 were used as the significance of the markers for all models in the study. Common MTAs detected in at least two GWAS models or in two environments were retained for downstream QTL analysis.

### QTL construction based on LD decay

To delineate QTLs associated with wheat blast resistance, linkage disequilibrium (LD) analysis was performed across all 21 wheat chromosomes using TASSEL v5.2 [42]. After genome-wide association analysis (GWAS), SNP markers significantly associated with wheat blast resistance were identified by applying a stringent Bonferroni correction to control for multiple testing, ensuring robust marker-trait associations. To construct QTL intervals from these significant SNPs, we investigated the local LD landscape surrounding each associated marker. Pairwise LD was estimated between the significant SNPs and their flanking markers using two metrics: r², which measures allelic correlation, and D′, a normalized measure of recombination between loci. LD heatmaps were generated for each chromosome to visualize the extent and structure of LD blocks by using TASSEL v5.2.

QTL regions were defined by identifying contiguous blocks of high LD surrounding each lead SNP, where LD thresholds of r² ≥ 0.70 and/or D′ ≥ 0.85 were used to delineate QTL boundaries. These regions were interpreted as representing genomic segments harbouring either causal variants or closely linked loci contributing to wheat blast resistance. To visualize the chromosomal distribution of QTLs, a phenogram was constructed using the Phenogram Web Tool developed by the University of Pennsylvania, which enables integration of QTL positions onto wheat genome ideograms [56]. Each QTL was assigned a unique identifier (e.g., *Qwb.cim*-*X*.*Y*, where *Qwb.cim* = QTL for wheat blast identified by CIMMYT, X denotes the chromosome and Y the serial order of QTLs identified on that chromosome).

For comparative mapping, the physical positions of known wheat blast resistance genes, i.e., *Rmg1*, *Rmg6*, *Rmg8* and the 2NS/2AS translocation segment, were retrieved from published literature and placed onto the same map. The physical distance between QTLs and these known genes was calculated in Megabases (Mb) using their reference genome positions of IWGSC RefSeq v2.1, [33,57] allowing evaluation of potential co-localization, tight linkage, or independence. This integrative LD-based QTL mapping approach facilitates the identification of both known and novel resistance loci and provides a genomic framework for downstream applications in fine-mapping, marker-assisted selection (MAS), and functional validation for wheat blast resistance.

## Results

### Phenotypic evaluation of wheat blast resistance across multi-environment trials

**Fig 1:**
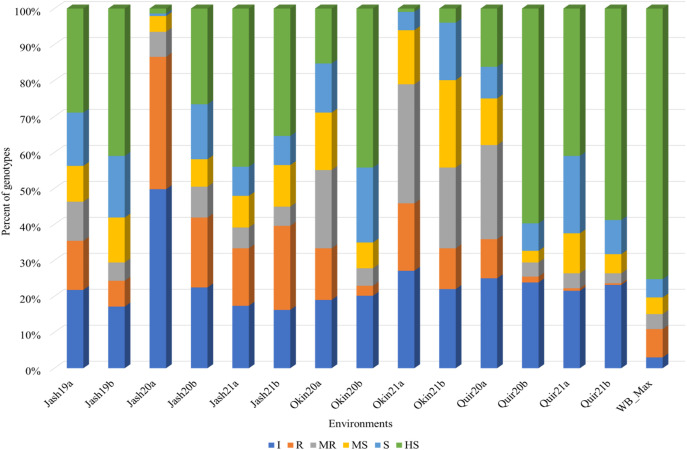
Distribution of wheat blast (WB) severity based on WB index for 432 diverse wheat genotypes evaluated under artificial inoculation across 14 environments in Bangladesh and Bolivia.Reaction categories: I = immune (no symptoms), R = resistant (WB index 1–10%), MR = moderately resistant (11–25%), MS = moderately susceptible (26–40%), S = susceptible (41–60%), and HS = highly susceptible (61–100%).

The wheat genotypes were phenotypically evaluated for wheat blast resistance under artificial inoculation across 14 distinct environments, representing seven growing seasons in three locations in Bangladesh and Bolivia. The maximum severity scored for each genotype across all environments was used to assess its overall resistance performance. Substantial phenotypic variation was observed among the genotypes, reflecting a broad spectrum of resistance responses (Fig 1). Only 3% of the entries exhibited complete resistance across all environments, while an additional 8% showed resistant reactions and 4% were classified as moderately resistant (S1 Table). In contrast, 5% displayed moderately susceptible to susceptible responses, and a striking 75% of the genotypes recorded highly severe infection (WB Index >60%) in at least one environment.

Environmental effects significantly influenced disease expression. Certain environments like Jash20a, Okin21a, and Okin21b exhibited relatively lower disease pressure, with most genotypes showing mild to moderate infection levels. In contrast, severe infection was observed in Jash19a, Jash21a, Jash21b (Bangladesh) and Okin20b, Quir20b, Quir21a, and Quir21b (Bolivia), suggesting that these environments were highly conducive to disease development. The contrast results among environments, including differences between first and second planting windows, suggest potential interactions between sowing time and environmental conditions favouring MoT infection. Within-environment analysis of variance revealed significant effects of both genotype and planting time on wheat blast (WB) index across all environments (Table 1). Notably, the second planting times in Bangladesh and, in a few cases, the first planting times in Bolivia were generally associated with higher WB indices, suggesting increased disease pressure under conditions favourable for blast development. These results highlight the influence of both genetic and temporal factors in shaping wheat blast response under artificial inoculation across diverse environments. Heritability on a genotype-mean basis ($\overset{\text{2}}{\underset{gm}{H}}$), derived from within environment estimates revealed that WB index was highly heritable (> 50%) in five environments, except Jashore 2020 and Okinawa 2021. Furthermore, the overall $\overset{\text{2}}{\underset{gm}{H}}$ for WB across environments was 0.897.

**Table 1:** Within-environment analysis of variance for WB index and the heritability estimates.

Environments	Average WB Index	F-value (genotype)	F-value (sowing time)	Heritability of mean $\overset{2}{\underset{gm}{H}}$
Jashore 19	44.9	3.23***	62.2***	0.691
Jashore 20	22	1.68***	396***	0.403
Jashore 21	46.7	7.92***	41.7***	0.874
Okinawa 20	38	3.21***	229***	0.689
Okinawa 21	19.4	1.83***	70.5***	0.452
Quirusillas 20	43	2.93***	368***	0.659
Quirusillas 21	54.2	8.49***	87.7***	0.882

**Fig 2:**
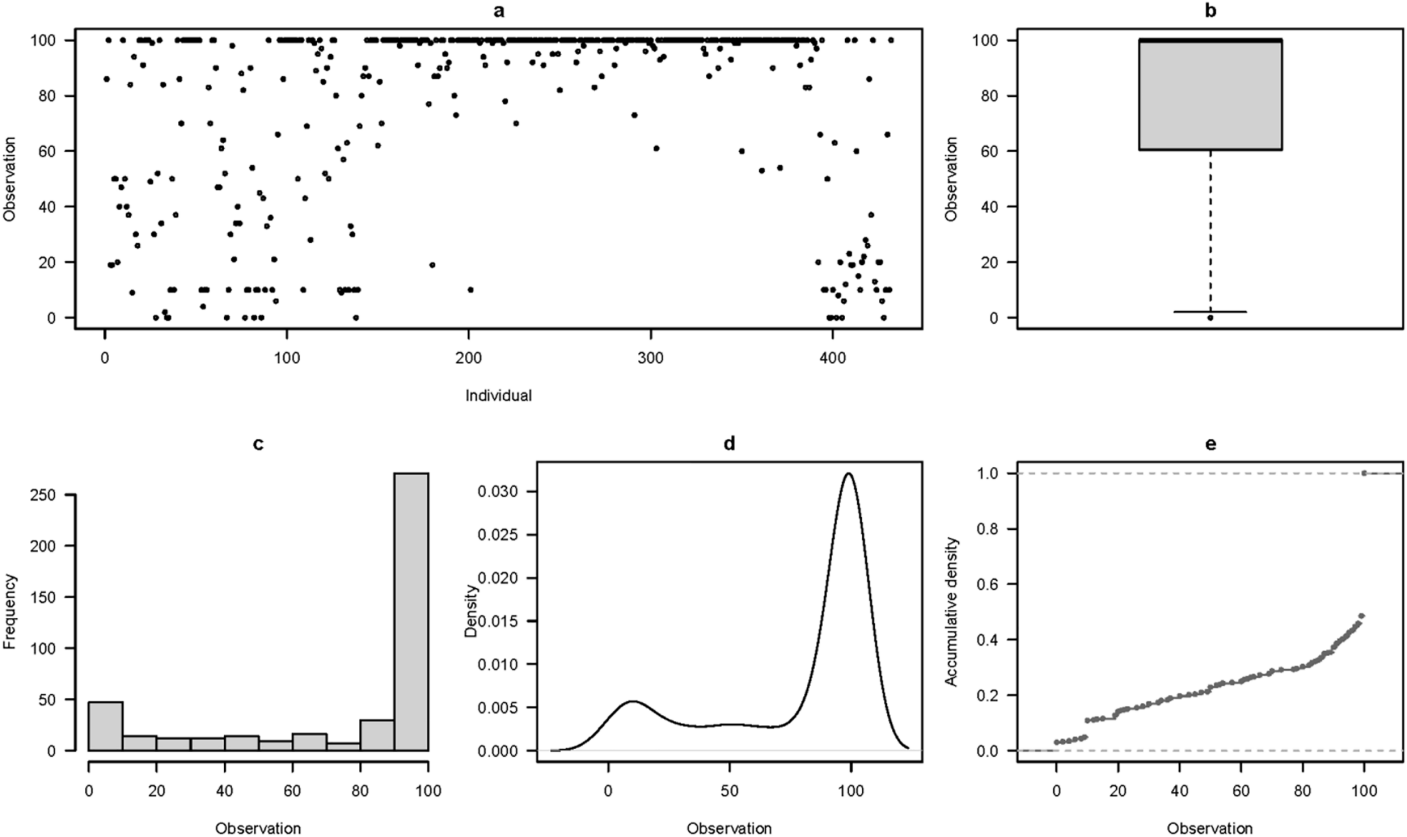
Field performance of the diverse wheat genotypes for wheat blast resistance across experiments. The figure summarizes maximum wheat blast scores pooled from all environments and includes the following visualizations: (a) scatter plot showing individual genotype scores; (b) box plot indicating that most genotypes fall within the upper quartile, suggesting high susceptibility in at least one environment; (c) histogram displaying the distribution of scores; (d) density plot illustrating the distribution pattern; and (e) cumulative density plot highlighting the prevalence of higher wheat blast scores. The wide range of values reflects substantial variability in resistance among the tested genotypes (S1 Fig illustrated for additional details of 14 environments, representing seven growing seasons in three locations).

The visualization of field data in Fig 2 and Fig S1 further supports these findings. The boxplot revealed a median position within the fourth quartile, indicating that the majority of genotypes experienced a high WB index (%) in at least one environment. The histogram, density, and cumulative distribution plots illustrated a skewed distribution toward high blast scores, reinforcing the dominance of susceptible responses. These trends collectively point to strong genotype-by-environment (G × E) interactions, wherein resistance expression varied considerably across testing conditions.

Despite the overall prevalence of susceptibility, a small subset of genotypes demonstrated consistent resistance across diverse environments, indicating their potential as sources of stable, broad-spectrum resistance. These genotypes warrant further genetic characterization and validation under both natural and artificial epidemic conditions.

**Fig 3:**
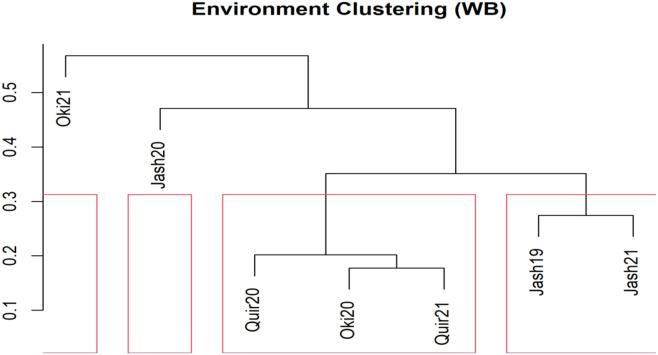
Hierarchical clustering of the environments using Ward’s method (Ward. D2).

Hierarchical clustering analysis identified four distinct environmental groups, indicating substantial heterogeneity in genotype performance across locations and years (Fig 3). The clustering of Jash19 and Jash21 suggests relative temporal consistency in genotype response at Jashore, supporting its classification as a stable testing environment. Likewise, the grouping of Oki20, Quir20, and Quir21 reflects similar genotype response patterns and possibly comparable environmental conditions or disease pressure across these sites, justifying their consideration as a common target population of environments (TPE). In contrast, Oki21 and Jash20 formed independent clusters, indicating pronounced year-specific effects that may represent distinct stress levels influencing wheat blast severity. Given the phenotypic performance of the genotypes regarding wheat blast index, the Maximum WB index was used for marker-trait association analysis, rather than BLUE. This approach ensures that genotypic susceptibility is accounted for in the final marker identification, as BLUE estimates may mask extreme susceptibility observed in specific unfavourable environments.

**Fig 4:**
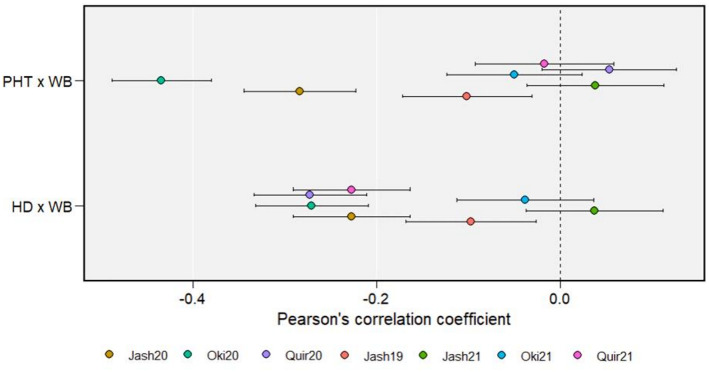
Association of WB with Heading Days (HD) and Plant Height (PHT).

To account for potential phenological escape in WB index response, we examined the correlations between WB and two key agronomic traits—Heading Days (HD) and Plant Height (PHT)—across environments (Table 2). The results indicate that WB index showed predominantly weak to moderate negative associations with HD and PHT; however, these relationships were inconsistent across environments (Fig 4).

**Table 2:** Statistical parameters of WB index along with days to heading and plant height, where different planting dates were considered as independent temporal evaluations.

Parameters	Heading days	Plant Height (cm)	WB Index (%)
Phenotypic variance	64.2	111	1030
Heritability on mean basis ($\overset{\text{2}}{\underset{gm}{H}}$)	0.881	0.821	0.897
Accuracy of Selection	0.939	0.906	0.947
Genotypic coefficient of variation	7.8	6.51	53.5
Residual coefficient of variation	9.98	8.86	49.8
Mean	61.78	86.86	40.2
Standard error of the mean	0.13	0.18	0.46
Standard deviation	10.42	14.36	35.77

Although late-heading and taller genotypes tended to exhibit slightly reduced blast severity in certain environments, the magnitude of these correlations was generally small, with confidence intervals often overlapping zero. This suggests that phenological escape does not affect much the variation in WB index, implying that genetic resistance plays a substantial role.

### Genome-Wide SNP distribution and population structure analysis

**Fig 5:**
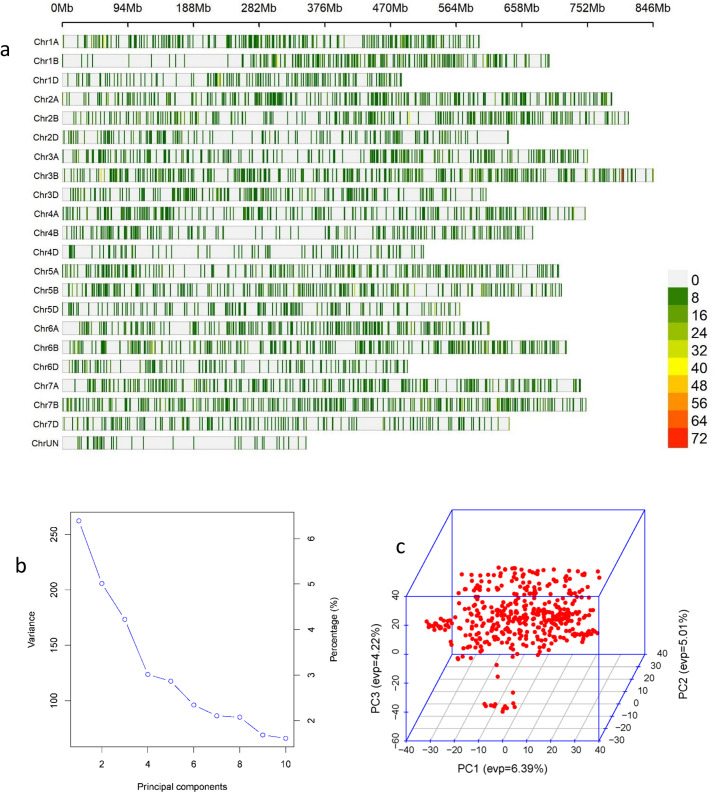
Genomic characteristics and principal component analysis (PCA) based on ddRAD-seq-derived SNP markers across 432 wheat genotypes.(a) Genome-wide marker density plot showing the distribution of SNPs across the wheat chromosomes; (b) Scree plot of eigenvalues indicating the proportion of total genetic variance explained by each principal component, used to determine the number of informative components; and (c) 3D PCA plot illustrating the genetic diversity and clustering patterns among the wheat genotypes based on the top three principal components.

The high-quality SNPs were distributed across the 21 chromosomes of the wheat genome, with the B genome exhibiting the highest marker saturation, followed by the A genome. The D genome displayed comparatively lower marker density, which is consistent with its narrower genetic diversity and lower polymorphism rate in hexaploid wheat. The highest number of markers were present in the B-genome (47.48%) followed by A-genome (40.76%) and the D-genome (10.89%). The heatmap visualization illustrates SNP density across genomic intervals, indicating adequate marker coverage for robust genome-wide association analysis (Fig 5a).

Principal Component Analysis (PCA) was conducted to explore the underlying genetic structure of the 432 genotypes. The scree PCA plot (Fig 5b) showed that the first three principal components explained 6.39%, 5.01%, and 4.22% of the total variance, respectively. The eigenvalue pattern indicates that the majority of genetic variation is captured within the top components, supporting their use in population structure correction in GWAS models. The 3D PCA plot (Fig 5c) revealed a moderately structured population, with genotypes showing continuous but distinguishable clustering. This reflects the presence of subpopulation structure and possible ancestral divergence among the genotypes. A good example is the small cluster clearly separated from the main group, being composed of synthetic wheat derivatives (Fig 5c). Such stratification necessitates correction during association analysis to control for spurious associations.

### Genotyping of the panel with markers for cloned *Rmg* genes

**Fig 6:**
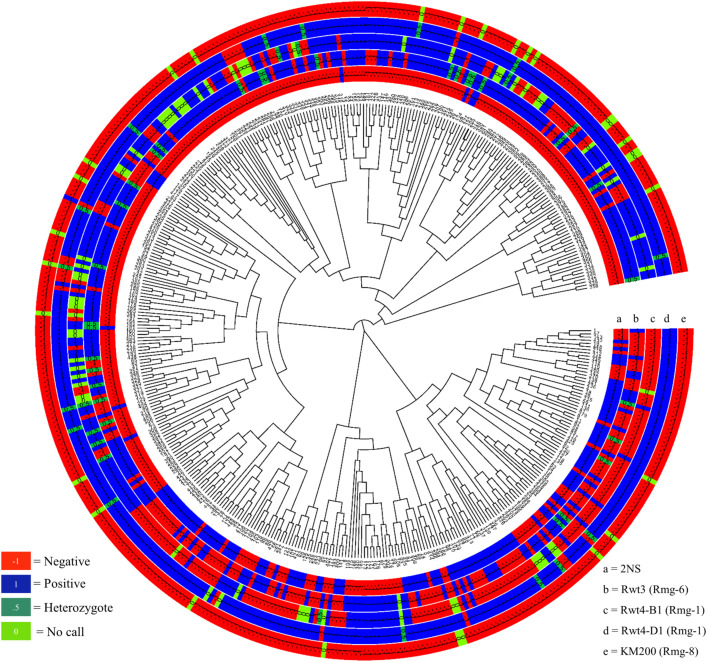
Neighbour-Joining tree based on genetic distances among the 432 bread wheat genotypes, showing the distribution of four Rwt (Rmg) genes and the 2NS translocation.

Genotyping results with the diagnostic markers indicated high frequencies of the non-MoT resistance genes, i.e., 64% of the genotypes have *Rmg6* (*Rwt3*), 52% have *Rwt4-1B* (allelic to *Rmg1*), and 95% have *Rmg1* (*Rwt4-1D*). In total, 29% of the genotypes have all three genes, whereas one genotype had none. As for the MoT resistance gene *Rmg8*, only one genotype was diagnosed to be positive (Fig 6). As expected, these non-MoT resistance genes showed no association with WB resistance. This was evident in genotypes carrying all three non-2NS resistance genes, which still exhibited moderate to high disease severity when the 2NS translocation was absent (S1 Table).

### Annotation of 2NS and *Rmg* loci into phylogenetic structure

The Neighbor-Joining (NJ) tree constructed from genome-wide SNP data revealed a well-defined population structure among the evaluated wheat genotypes (Fig 6). Integration of molecular marker data with the NJ tree showed non-random distribution patterns of resistance loci. The 2NS translocation was predominantly associated with a distinct and cohesive cluster of genotypes, suggesting a shared genetic background or selective retention of this segment. Similarly, *Rwt3 (Rmg6)* and both homeologous copies of *Rwt4—Rwt4-1B* and *Rwt4-1D* (*Rmg1*)—were enriched within specific sub-clades, indicating a structured deployment of these loci across the germplasm. A total of 182 genotypes carried both *Rwt4-1B* and *Rwt4-1D*. Notably, their co-occurrence across several phylogenetic branches supports the hypothesis that these loci have been co-maintained, potentially due to their location on homeologous chromosomes and/or co-selection during breeding, consistent with expected homeologous chromosome patterns in wheat. In contrast, the *Rmg8* gene was detected in only a single genotype, indicating its extremely low frequency within the current panel. Overall, the annotated NJ tree highlights a non-random, structured distribution of wheat blast resistance genes, reflecting clear genetic stratification among the genotypes.

### Linkage Disequilibrium (LD) decay analysis

Genome-wide LD analysis revealed genome-specific patterns across the 21 wheat chromosomes (S3 Fig). Consistent with previous findings, chromosomes of the D genome exhibited larger and more continuous LD blocks, reflecting lower historical recombination rates and reduced marker coverage. In contrast, the A and B genomes displayed a more fragmented LD landscape, indicative of more frequent historical recombination events and faster LD decay. These LD patterns align with the known evolutionary dynamics of hexaploid wheat and provide a genomic context for interpreting association signals. Genome-wide LD decayed below the threshold (defined as the 95th percentile of background r²) at approximately 144 Kb, indicating relatively rapid LD decay and high mapping resolution in the association panel (S4 Fig).

### Genome-wide association study for wheat blast resistance

Six different GWAS models—MLM, MLMM, SUPER, BLINK, FarmCPU, and GLM—were employed using GAPIT package in R to ensure robust and comprehensive detection of associated markers. Across the models, a total of 1,121 significant SNPs were identified after applying Bonferroni correction (α = 0.05/7,211 SNPs (p = 6.93 × 10 ⁻ ⁶; − log₁₀(p) = 5.16)), distributed across multiple chromosomes. These associations suggest the presence of diverse resistance loci effective under varying environmental conditions. Notably, strong associations were observed on chromosomes 2A, 3A, 3B, 5A, and 6B, highlighting these regions as potential hotspots for WB resistance. Within the D genome, chromosome 4D showed the highest number of MTAs, with 76 out of 80 significant SNPs in the genome, indicating that WB resistance may have been introgressed through natural wide crosses involving 4D, and that this chromosome could be a key target for future breeding.

**Fig 7:**
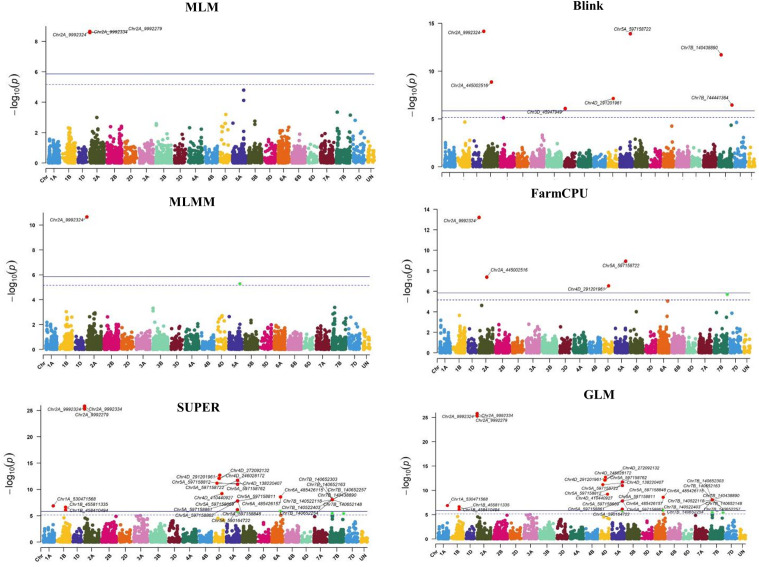
Manhattan Plots Showing GWAS Results Using Six Different Models. The plots were drawn based on the pooled highest wheat blast (WB) scores across 14 environments. Individual Manhattan plots for each environment are presented in S2 Fig.

Among the tested models, MLM, MLMM, BLINK, and FarmCPU demonstrated higher detection power and resolution, consistent with their previously reported effectiveness in analysing complex traits with moderate to high heritability. The Manhattan plots in Fig 7 represent associations based on the highest WB index values observed for each genotype across all environments. Additional Manhattan plots for individual environments are presented in Fig. S2. These SNP markers identified from GWAS were then used for QTL identification.

### QTL identification

**Fig 8:**
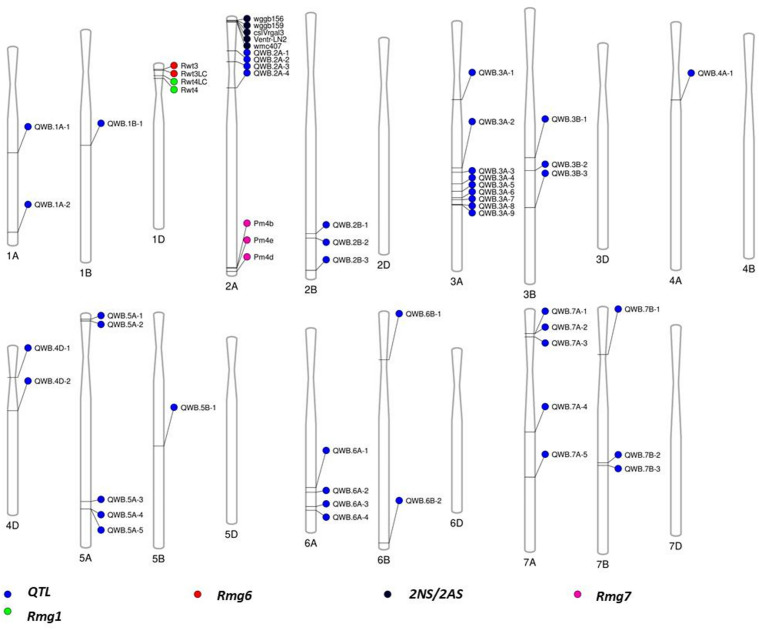
Genome wide QTL distribution for wheat blast resistance identified from the 14 different field experiments using six different models. Integrated wheat blast resistance QTL map constructed using a phenogram-based visualization tool. A total of 45 QTLs associated with wheat blast resistance are displayed in blue dots across the wheat genome. Regions enriched with QTLs are observed particularly on chromosomes 2A, 3A, 3B, 5A, 5B, 6A, 6B, 7A, and 7B. The map also shows the position of several Rmg (Rwt) genes and the 2NS/2AS translocation.

A total of 45 QTL were identified across the wheat genome, each supported by statistically robust associations derived from Bonferroni-adjusted thresholds and validated through significant linkage disequilibrium (LD) structures, reflecting both the strength and reliability of these signals (Table 3). To enhance the understanding of the genetic architecture underpinning wheat blast resistance, a comprehensive QTL map was developed using a phenogram-based visualization approach [56]. This integrated map delineates both genotyped *Rmg* (*Rwt*) genes and QTLs identified in the current study. The QTLs were predominantly distributed across chromosomes 2A, 3A, 3B, 5A, 5B, 6A, 6B, 7A, and 7B (Fig 8).

**Table 3:** QTLs associated with wheat blast resistance identified across wheat chromosomes, showing the leading SNP, mean additive allelic effect size (β), genomic interval, number of SNPs per QTL, QTL length, overlap status, R², D′, GWAS model, and detection environments.

SL	QTLs	Chromosome	Leading SNP	Mean allelic effect size of leading SNP (β)	Genomic Region (bp)	SNPs inside the QTL	QTL Length (bp)	Single/Overlap QTL	R²	D’	Model	Environments		
												Jashore, BD	Okinawa, Bolivia	Quirusillas, Bolivia
1	*Qwb.cim-1A.01*	1A	317732139	−13.52	345108382−300796734	317732139	44311648	*Single QTL*	1	1	Blink; FarmCPU; MLM; SUPER	–	–	Quir20a
2	*Qwb.cim-1A.02*	1A	559692371	−4.33	559692399−559692371	559692371	28	*Single QTL*	1	1	Blink; FarmCPU;GLM	–	Okin20a	–
3	*Qwb.cim-1B.01*	1B	346686341	7.56	380918421−346686341	346686341	34232080	*Single QTL*	0.7983	0.9008	GLM; SUPER	–	Okin20a	–
4	*Qwb.cim-2A.01*	2A	9992279	11.29	9992334−9992279	9992279 9992324 9992334	55	*Single QTL*	0.9893	1	Blink FarmCPU GLM MLM MLMM SUPER	Jash19a Jash19b Jash20b Jash21a Jash21b	Okin20a Okin20b Okin21a Okin21a Okin21b	Quir20a Quir20b Quir21a Quir21b
5	*Qwb.cim-2A.02*	2A	98005103	8.75	98005272−98005103	98005103 98005272	169	*Single QTL*	0.9445	1	SUPER	–	–	Quir20b Quir21b
6	*Qwb.cim-2A.03*	2A	107511808	11.07	153172033−107511808	107511808 107511828 107511958 124735818 124769697 124769864 131341813 132018063 151353000 153172033	45660225	16 Overlap QTLs	0.7199	0.8647	SUPER	–	Okin20b	Quir20b Quir21b
7	*Qwb.cim-2A.04*	2A	154934390	11.12	299808833−154934390	154934390 154960678 161227524 212018647 240170164 282783107	144874443	28 Overlap QTLs	0.7015	0.8963	Blink; FarmCPU; GLM; SUPER	Jash19a Jash21a	Okin20b	Quir20b Quir21b
8	*Qwb.cim-2B.01*	2B	666473489	6.65	675957356−666473489	666473489 667289691 668204109 668204249 668204295 669029040 675957356	9483867	*Single QTL*	0.7441	0.988	Blink GLM SUPER FarmCPU	Jash19b Jash21a	Okin20b Okin20b	Quir20b Quir21a Quir21b
9	*Qwb.cim-2B.02*	2B	675957356	8.45	680996234−675957125	675957356 680996170 680996234	5039109	10 Overlap QTLs	0.768	0.96	GLM Blink FarmCPU SUPER	–	Okin20a Okin20b	Quir21a Quir21b
10	*Qwb.cim-2B.03*	2B	779501416	−6.99	779501416−776929426	779501416	2571990	2 Overlap QTLs	0.7055	0.8443	GLM SUPER	Jash21a	Okin20b	
11	*Qwb.cim-3A.01*	3A	210714908	9.24	287118567−210714908	210714908 223282861 232807564 252889978 287118567	76403659	4 Overlap QTLs	0.7208	0.9035	GLM SUPER	–	Okin20a Okin20b	Quir20b Quir21a Quir21b
12	*Qwb.cim-3A.02*	3A	336993000	9.28	444505901−336993000	336993000 356891988 441070513 441524679 441524686 441524779 441524883 441524947 443260811 443260828 443260994 443261002 443261077 444505901	107512901	12 Overlap QTLs	0.7109	0.9402	GLM	–	Okin20a Okin20b	Quir20b Quir21a Quir21b
13	*Qwb.cim-3A.03*	3A	444505947	−12.42	463294783−444505947	444505947 447496978 449983833 449983834 449983866 449983935 450806302 451821639 455715905 455716129 455716160 461027854 461028067 462570191 463294667 463294783	18788836	13 Overlap QTLs	0.9249	0.9741	GLM Blink	Jash21a Jash21b	Okin20a Okin20b	Quir20b Quir21a Quir21b
14	*Qwb.cim-3A.04*	3A	490633681	−5.47	490633695−488220344	490633681 490633695	2413351	2 Overlap QTLs	0.7106	0.9681	Blink GLM MLMM SUPER	Jash20a	–	–
15	*Qwb.cim-3A.05*	3A	507740854	−8.55	514916200−507740854	507740854 507740999 507741062 507741089 507747620 507747758 511508954 513451418 514785224 514785236 514785397 514785409 514785440 514916200	7175346	8 Overlap QTLs	0.8072	0.9414	GLM SUPER	Jash21a Jash21b	Okin20a Okin20b	Quir20b Quir21b
16	*Qwb.cim-3A.06*	3A	532543119	−14.46	532720891−532543119	532543119 532543126 532720891	177772	2 Overlap QTLs	0.9737	1	GLM SUPER	Jash19b Jash21a Jash21b	Okin20a Okin20b	Quir20b Quir21a Quir21b
17	*Qwb.cim-3A.07*	3A	539707365	−14.97	539707597−537183367	539707365 539707405 539707428 539707538	2524230	3 Overlap QTLs	0.8965	0.9897	Blink GLM SUPER	Jash19b Jash21a Jash21b	Okin20a Okin20b	Quir20b Quir21a Quir21b
18	*Qwb.cim-3A.08*	3A	552881256	−14.71	553499384−552881052	552881256 552881287 553499247 553499255	618332	7 Overlap QTLs	0.9031	1	GLM Blink FarmCPU SUPER	Jash19b Jash21a Jash21b	Okin20a Okin20b	Quir20b Quir21a Quir21b
19	*Qwb.cim-3A.09*	3A	554708776	−16.71	558546840−554708776	554708776 554708807 554708989 558546840	3838064	3 Overlap QTLs	0.7492	0.9175			Okin20b	Quir20b Quir21b
20	*Qwb.cim-3B.01*	3B	449868361	−9.56	467974603−449868361	449868361 449868361 467974603	18106242	*Single QTL*	0.8484	0.9664	Blink FarmCPU GLM SUPER	Jash19a Jash20b Jash21a Jash21b	–	Quir21a Quir21b
21	*Qwb.cim-3B.02*	3B	468673457	−9.66	546274410−468673457	469284908 469285105 482203951 482203959 482203971 482203974 482204026 482204040 489699710 489699718 489699734 489699782 489699855 489699908 491675431 491675439 491675666 546274410	77600953	7 Overlap QTLs	0.71	0.8426	Blink FarmCPU GLM SUPER	Jash19a Jash19b Jash20b Jash21a Jash21b	Okin20a	Quir20a Quir21a Quir21b
22	*Qwb.cim-3B.03*	3B	601935925	−9.91	601936131−601935887	601935925	244	4 Overlap QTLs	1	1	FarmCPU GLM	Jash21b	Okin20a	–
23	*Qwb.cim-4A.01*	4A	231689567	−5.7	235193897−141129541	231689567	94064356	*Single QTL*	0.8166	0.9333	Blink FarmCPU MLMM SUPER	Jash20a	Okin20b	–
24	*Qwb.cim-4D.01*	4D	91702309	−6.73	91702309−76431551	91702309	15270758	*Single QTL*	0.8368	0.9412	GLM SUPER	–	Okin20b	–
25	*Qwb.cim-4D.02*	4D	138220407	12.79	291201961−138220407	138220407 194314945 194315018 194315048 194315109 194315120 194315122 246028172 272092132 291201961	152981554	3 Overlap QTLs	0.9283	1	Blink FarmCPU GLM SUPER	Jash19b Jash21a Jash21b	Okin20a Okin20b	Quir20b Quir21a Quir21b
26	*Qwb.cim-5A.01*	5A	11546777	−8.12	11858109−11546777	11546777 11857876 11857891 11858092 11858109	311332	4 Overlap QTLs	0.9261	0.9807	GLM	Jash21b		Quir20b
27	*Qwb.cim-5A.02*	5A	16887844	9.67	16888095−16887844	16887844 16887869 16887874 16887882 16887890 16887904 16888015 16888095	251	7 Overlap QTLs	1	1	Blink GLM SUPER	Jash21a Jash21b	Okin20b	Quir20b
28	*Qwb.cim-5A.03*	5A	574076028	7.83	574076205−574076028	574076028 574076205	177	5 Overlap QTLs	1	1	GLM		Okin20b	Quir20b
29	*Qwb.cim-5A.04*	5A	590164722	−8.36	597158862−597158669	590164722 597158669 597158722 597158762 597158811 597158812 597158848 597158861 597158862	193	4 Overlap QTLs	1	1	Blink FarmCPU GLM MLM MLMM SUPER	Jash19a Jash19b Jash21b	Okin20a Okin20b	Quir20b Quir21a Quir21b
30	*Qwb.cim-5A.05*	5A	597476187	7.84	597476270−597476187	597476187 597476191 597476192 597476270	83	3 Overlap QTLs	1	1	GLM SUPER	Jash19b	Okin20b Okin21b	Quir20b Quir21b
31	*Qwb.cim-5B.01*	5B	405326872	5.4	405326872−403758171	405326872	1568701	2 Overlap QTLs	0.9066	0.9521	Blink GLM SUPER	–	Okin20a	–
32	*Qwb.cim-6A.01*	6A	485426115	7.74	485426157−485426115	485426115 485426157	42	*Single QTL*	1	1	GLM SUPER	Jash21b	Okin20a Okin20b	Quir20b Quir21a
33	*Qwb.cim-6A.02*	6A	499689186	8.32	499697683−499689186	499689186 499689239 499689260 499689326 499689332 499689342 499697463 499697497 499697501 499697634 499697683	8497	7 Overlap QTLs	0.9399	0.9794	GLM SUPER	Jash19a Jash19b Jash21a Jash21b	Okin20a Okin20b	Quir20b Quir21a Quir21b
34	*Qwb.cim-6A.03*	6A	544174290	7.24	544174338−544174187	544174290	151	3 Overlap QTLs	1	1	Blink FarmCPU	Jash21b	–	–
35	*Qwb.cim-6A.04*	6A	555453648	8.75	555453840−555453648	555453648 555453779 555453840	192	2 Overlap QTLs	1	1	Blink GLM SUPER	Jash21a Jash21b	–	–
36	*Qwb.cim-6B.01*	6B	142160772	7.17	156854946−142160772	142160772 156854946	14694174	*Single QTL*	0.7873	0.9317	Blink FarmCPU GLM	Jash19a Jash20b	–	Quir21b
37	*Qwb.cim-6B.02*	6B	708091225	5.82	708091321−708091225	708091225	96	*Single QTL*	0.8478	1	Blink FarmCPU	–	–	Quir20a Quir21b
38	*Qwb.cim-7A.01*	7A	68671672	10.17	68671914−68671672	68671672 68671672 68671722 68671756 68671891 68671900 68671914	242	2 Overlap QTLs	0.8787	1	FarmCPU GLM	–	–	Quir21a Quir21b
39	*Qwb.cim-7A.02*	7A	73470952	10.55	73470987−73470952	68671672 68671722 68671756 68671891 68671900 68671914	35	*Single QTL*	1	1	GLM	–	Okin20b	Quir20b Quir21a Quir21b
40	*Qwb.cim-7A.03*	7A	80160295	8.67	80160465−80160295	80160295 80160453 80160465	170	2 Overlap QTLs	1	1	GLM	–	–	Quir20b Quir21b
41	*Qwb.cim-7A.04*	7A	372589417	−7.95	372589444−372589412	372589417	32	4 Overlap QTLs	1	1	GLM	–	–	Quir20b Quir21b
42	*Qwb.cim-7A.05*	7A	512534675	−7.56	512719367−512534675	512534675 512534676 512719367	184692	2 Overlap QTLs	0.9452	0.9813	GLM	Jash20b Jash21a	Okin20b	Quir20b Quir21b
43	*Qwb.cim-7B.01*	7B	132096752	7	140522225−118413417	132096752 132096816 132096817 132096825 132096962 140438703 140438760 140438767 140522131 140522138	22108808	4 Overlap QTLs	0.7988	0.9537	Blink GLM SUPER	Jash21a	Okin20b	Quir20b Quir21a
44	*Qwb.cim-7B.02*	7B	474346365	−8.03	474346365−459300575	474346365	15045790	2 Overlap QTLs	0.9172	0.9806	GLM	Jash21a Jash21b	–	–
45	*Qwb.cim-7B.03*	7B	482724515	−8.22	482724515−459300575	482724515	23423940	2 Overlap QTLs	0.8811	0.9431	GLM	Jash21a Jash21b	–	–

Among these, chromosome 2A emerged as particularly significant due to the presence of four QTLs (*Qwb.cim-2A.01* to *Qwb.cim-2A.04*), with the first corresponding to the well-characterized 2NS/2AS translocation segment. The last three QTL on this chromosome exhibited long distance up to 88 Mb from the 2NS translocation, suggesting independent resistance factors. No QTL was detected in the *Rmg7* region on chromosome 2AL, which is closely linked to the powdery mildew resistance genes *Pm4b*, *Pm4e*, and *Pm4d* [41]. The absence of any significant QTLs on chromosome 1D confirmed that *Rmg1* and *Rmg6*, being non-MoT resistance genes located on 1D, did not contribute to WB resistance.

The remaining 41 *QWB* loci, which are located in genomic regions without known *Rmg* genes, may represent new or previously uncharacterized candidate regions for blast resistance. While these regions do not overlap with reported *Rmg* loci, further comparison with previously mapped QTL is needed to determine whether they coincide with earlier resistance signals or are truly novel. These loci are distributed across 3A, 3B, 5A, 5B, 6A, 6B, 7A, and 7B, with particularly high QTL density on 3A, 5A, and 7A.

To enhance the biological and breeding relevance of the detected loci, the mean additive allelic effect size (β) of each leading SNP and the QTL overlap status were estimated and are presented in Table 3. The mean allelic effects ranged from −16.71 to 12.79 WB index units, indicating substantial variation in the magnitude and direction of allelic contributions to wheat blast resistance. Negative β values represent alleles associated with reduced disease severity (favorable alleles), whereas positive β values indicate increased susceptibility.

Several QTLs exhibited large allelic effects (|β| > 10), particularly on chromosomes 2A, 3A, 3B, 4D, and 7A, suggesting major genomic regions contributing to blast resistance. Notably, Qwb.cim-3A.09 showed the largest negative allelic effect (β = −16.71), highlighting its potential value for marker-assisted selection.

Based on physical co-localization across environments and models, QTLs were further classified as single or Overlap regions. While several loci were detected as single QTLs with consistent effects (e.g., *Qwb.cim-1A.01*, *Qwb.cim-6A.01*), a substantial proportion formed Overlap QTL clusters, particularly on chromosomes 2A, 3A, 5A, and 7A. These Overlap regions likely represent stable genomic hotspots repeatedly identified across multiple environments and GWAS models, reinforcing their robustness and potential utility in breeding programs.

## Discussion

Phenotypic evaluation of wheat blast resistance in this panel of wheat genotypes across multi-environment trials revealed that a small proportion (15%) of genotypes were consistently resistant (I to MR reactions), whereas the majority (75%) exhibited a wheat blast index greater than 50% in at least one environment. This skewed distribution highlights the scarcity of stable resistance in current germplasm and underscores the persistent threat of MoT under conducive conditions [2,3,58]. Only a small fraction of genotypes (3%) showed consistent resistance across all test environments, marking them as potential sources of durable resistance.

Strong genotype-by-environment (G × E) interactions were evident, with considerable variation in disease expression across sites and seasons. Environments such as Jash19a, Jash20a, and Quir21a were favourable for disease development, while others, like Okin21a and Jash20a, showed lower disease pressure. These findings support previous reports indicating that wheat blast severity is highly influenced by local microclimatic factors, particularly temperature and humidity during heading and grain filling stages [3,9,59,60]. The observed difference in disease incidence between early and late sowing windows may also reflect environmental shifts impacting pathogen virulence and host susceptibility.

The ddRAD-seq genotyping provided high-density SNP coverage across the wheat genome, with a higher marker density in the B and A genomes than in the D genome, aligning with established patterns of genomic diversity in hexaploid wheat [61].

This approach was selected as a cost-effective and scalable genotyping strategy suitable for evaluating a diverse wheat panel across multiple environments. After applying stringent quality filtering, a set of 7,211 SNPs was retained from the initial 82,948 SNPs. This marker density is well-justified for GWAS, as similar marker densities in studies using reduced-representation sequencing have proven sufficient for detecting major and stable resistance loci under strong disease pressure [17,22,62,63]. Principal Component Analysis (PCA) revealed a moderately structured population, which necessitated the inclusion of population structure covariates in GWAS to mitigate spurious associations [64]. The NJ tree further illustrated this structure, revealing distinct clustering patterns corresponding to the presence of known resistance loci. The integration of gene-specific markers revealed that the 2NS/2AS translocation and cloned resistance genes (*Rwt3*, *Rwt4-B1*, and *Rwt4-D1*), were unevenly distributed. Notably, 36% of genotypes lacked *Rwt3*, and nearly half did not carry *Rwt4-B1*, despite their documented contribution to non-host resistance against multiple *Magnaporthe* pathotypes [65].

The identification of 45 QTLs across multiple chromosomes through GWAS and LD mapping significantly expands the known genetic architecture of wheat blast resistance. The concentration of four QTLs on chromosome 2A reinforces its pivotal role in resistance expression [7,23,58,66]. No QTL was identified on chromosome 1D near the known resistance genes *Rmg1* and *Rmg6*, because they are non-MoT resistance genes and are not expected to show significant effects under field experiments inoculated with MoT isolates. On the contrary, *Rmg8* on chromosome 2B is a well-known MoT resistance gene that has shown good resistance effects in greenhouse experiments [67]. However, its effects under field conditions need to be further validated. No QTL was identified on the chromosomal region harbouring this gene, which was due to the extremely low frequency in this panel (only one genotype carried this gene) that made it impossible to evaluate its effects in the experiments. More specifically designed experiments are thus needed to evaluate the effects of *Rmg8* on WB resistance in field experiments. The clustering of QTLs in the genome may indicate genomic hotspots for resistance, potentially reflecting loci with pleiotropic effects, multiple tightly linked resistance genes, or regions involved in quantitative disease response mechanisms such as reactive oxygen species (ROS) regulation, pathogen-associated molecular pattern (PAMP) signalling, or systemic acquired resistance (SAR).

It is noteworthy that there are many lines lacking one or two of the tested non-host resistance genes, and there is one line lacking all three genes. Lacking *Rwt3* means those wheat genotypes are naturally susceptible to the *Magnaporthe oryzae* pathotype *Lolium* (MoL), in addition to their susceptibility to *Triticum* pathotype (MoT), imposing additional risks to farmers growing such varieties [65]. Lacking both *Rwt3* and *Rwt4* will make the wheat genotypes susceptible to *Avena* pathotype (MoA), in addition to MoT and MoL, and become more vulnerable to *Eleusine* pathotype (MoE), a pathotype known to be prevalent in India that causes the blast disease in finger millet [4]. Another implication of the results is that the genotypes without *Rwt4-D1* (*Rmg1*) may have difficulties in utilizing *Rmg8* for improving WB resistance, because the former is known to be a prerequisite for the latter to be functional under the scenario that MoT acquires the virulence factor *PWT4* [14]. Therefore, it is beneficial for breeders to introduce both *Rmg8* and *Rmg1* (if not already present in the recipient genotype) to prevent the situation of resistance breakdown of *Rmg8* in future. Collectively, this high-resolution QTL map offers a robust framework for marker-assisted selection (MAS) in wheat blast resistance breeding. It not only validates known sources of resistance but also provides novel targets for fine mapping, gene cloning, and functional characterization, which are crucial for designing durable, broad-spectrum resistance in elite wheat cultivars. The integration of these *Qwb.cim* loci into breeding pipelines holds promise for enhancing resilience against MoT under diverse agro-ecological settings.

Together, these findings offer a comprehensive framework for deploying diverse resistance loci in breeding programs. The integration of phenotypic data, population genomics, LD landscape, and QTL information presents opportunities to design targeted breeding strategies for durable resistance. Future efforts should prioritize the pyramiding of complementary resistance loci, particularly those exhibiting stable performance across environments, to enhance resilience against wheat blast under variable climatic conditions.

### Concluding remarks and recommendations

The present study provides a comprehensive genome-wide insight into the QTL associated with wheat blast resistance in a diverse panel of Bangladeshi wheat genotypes. A total of 45 *Qwb.cim* loci were identified across multiple chromosomes, with several novel loci detected outside the well-characterized 2NS translocation region. These findings reflect the complex and polygenic nature of resistance to wheat blast in Bangladeshi germplasm and underscore the presence of alternative resistance sources beyond the widely utilized 2NS locus. The identification of QTLs on chromosomes 1B, 2B, 4A, 5A, 6A, and 7A—particularly those consistently associated with resistance across multiple environments—provides valuable targets for marker-assisted selection (MAS). These loci represent a foundation for future efforts in fine mapping, functional validation, and candidate gene discovery. Integrating these findings into breeding pipelines will be critical to reduce reliance on 2NS-based resistance, which may be prone to pathogen evolution and potential breakdown over time.

Future work should prioritize validation of these loci using biparental populations and near-isogenic lines to ensure their stability and effectiveness. Fine mapping of major QTL regions is recommended to identify tightly linked markers and candidate genes for cloning. Pyramiding of non-2NS QTLs with existing resistance genes, including the 2NS segment, could enhance the durability and spectrum of resistance in future cultivars. Additionally, the integration of genomic selection approaches using the identified loci could significantly accelerate breeding for wheat blast resistance.

Given the transboundary threat of MoT, collaborative regional efforts involving phenotyping in diverse hot spot environments, sharing of germplasm, and joint validation of resistance loci are essential. The outcomes of this study provide a foundation for developing improved wheat varieties with durable resistance to wheat blast, ensuring yield stability and food security in Bangladesh and other wheat-growing regions of South Asia.

## Supporting information

S1 Fig Visualization of field phenotype details for wheat blast score in 14 different environments of Bangladesh and Bolivia. (PDF)

S2 Fig Manhattan plot for MTA with six different models. (PDF)

S3 Fig Variation in Linkage Disequilibrium across wheat chromosomes in the diverse bread wheat germplasm panel, visualized by r² and D’ values. (PDF)

S4 Fig Genome-wide LD decay showing the decline of mean pairwise linkage disequilibrium (r²) with increasing physical distance (Kb) and the estimated LD-decay distance (144 Kb). (PDF)

S1 Table List of genotypes showing immune, resistant, or moderately resistant reactions across locations, along with their genetic composition for 2NS, *Rmg1*, *Rmg6*, and *Rmg8.* (PDF)

S1 File GWAS Methods in GAPIT to Identify SNPs for Wheat Blast Resistance. (PDF)
