# Supplementary material for: Field screening and genetic mapping of wheat blast resistance for a panel of common wheat from Bangladesh
Source: PLoS One. 2026 Jun 11;21(6):e0349201. doi: 10.1371/journal.pone.0349201 (PMC13258015; doi:10.1371/journal.pone.0349201)
Supplement: S1 Fig — (PDF) [file pone.0349201.s001.pdf]

S1 Fig. Visualization of field phenotype details for wheat blast score in 14 different environments of Bangladesh and Bolivia

a) environment of Bangladesh.

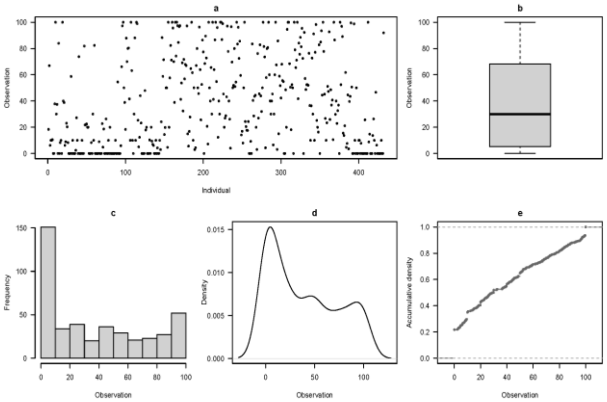

Jash 19a

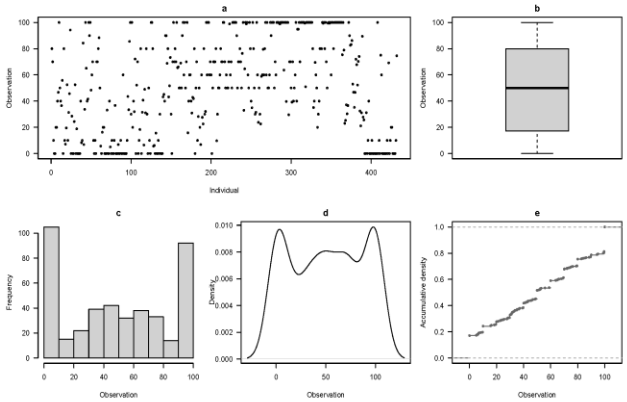

Jash 19b

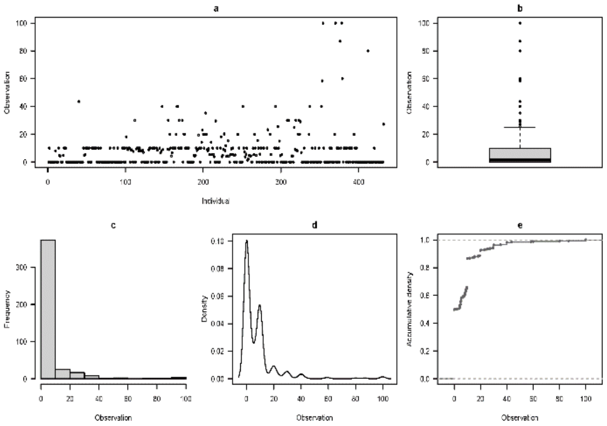

Jash 20a

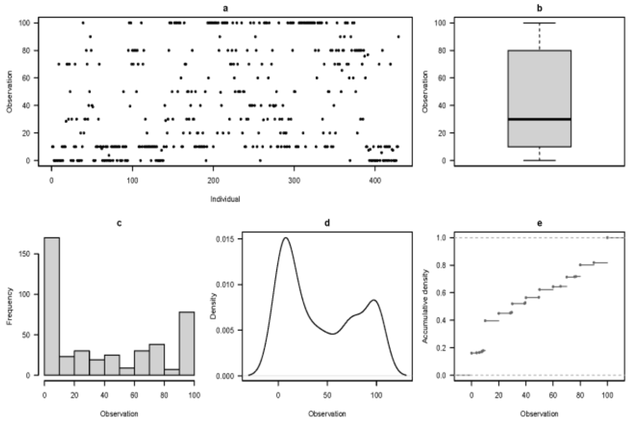

Jash 20b

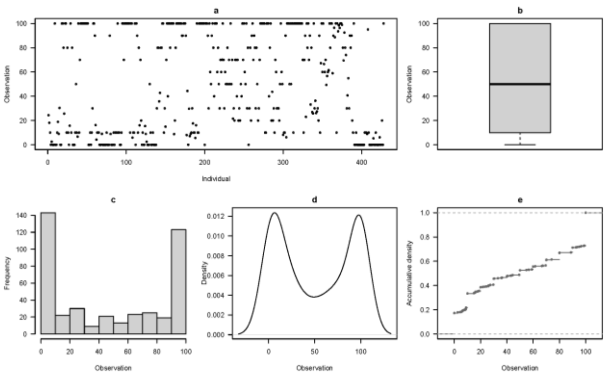

Jash 21a

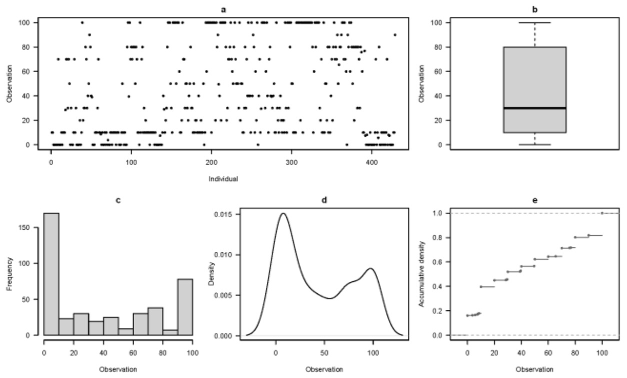

Jash 21b

b) environment of Bolivia.

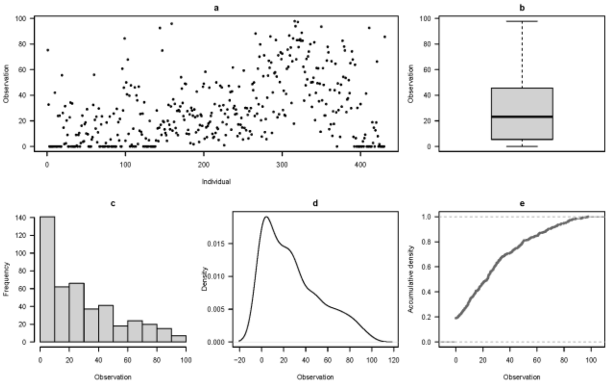

Oki 20a

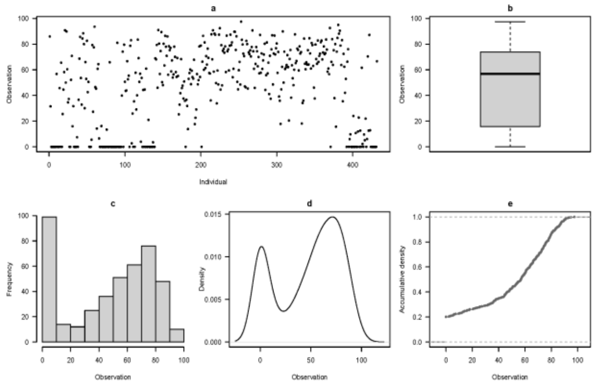

Oki 20b

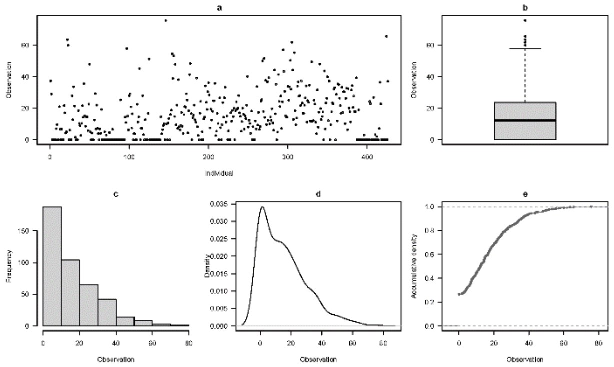

Oki 21a

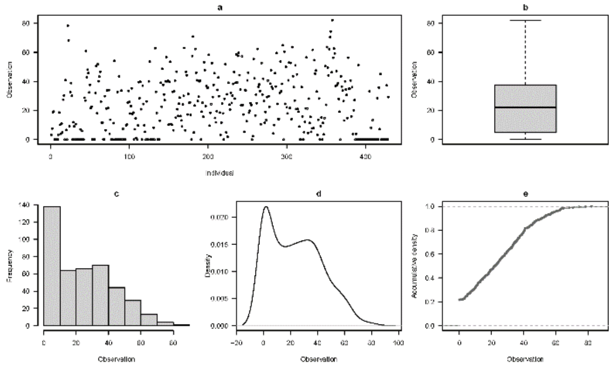

Oki 21b

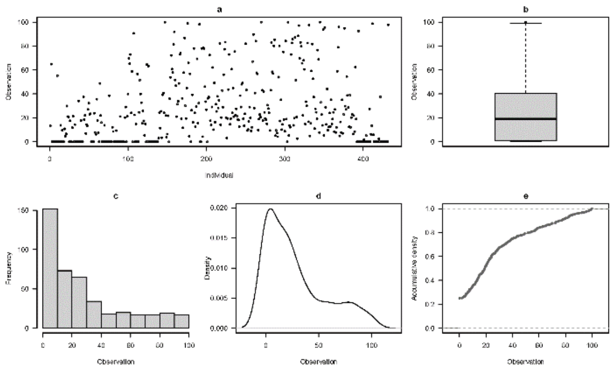

Quir 20a

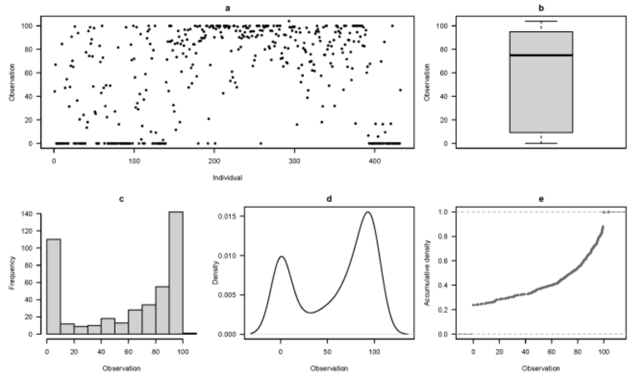

Quir 20b

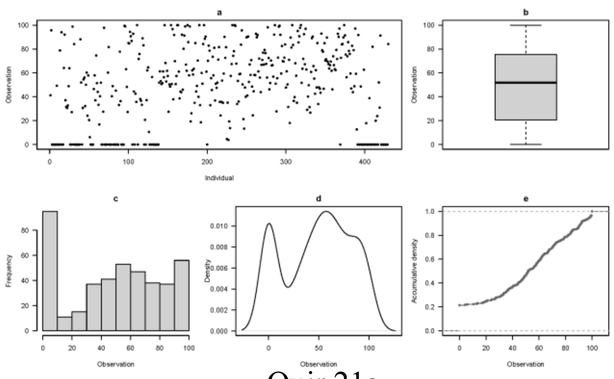

Quir 21a

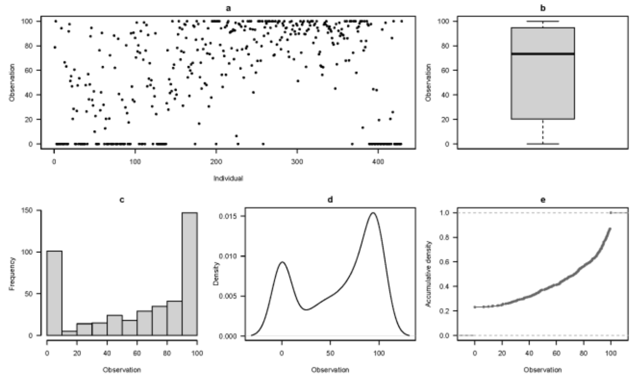

Quir 21b
