# Supplementary material for: Field screening and genetic mapping of wheat blast resistance for a panel of common wheat from Bangladesh
Source: PLoS One. 2026 Jun 11;21(6):e0349201. doi: 10.1371/journal.pone.0349201 (PMC13258015; doi:10.1371/journal.pone.0349201)
Supplement: S2 Fig — (PDF) [file pone.0349201.s002.pdf]

S2 Fig. Manhattan plot for MTA with six different models

a) Jashore, Bangladesh 2019

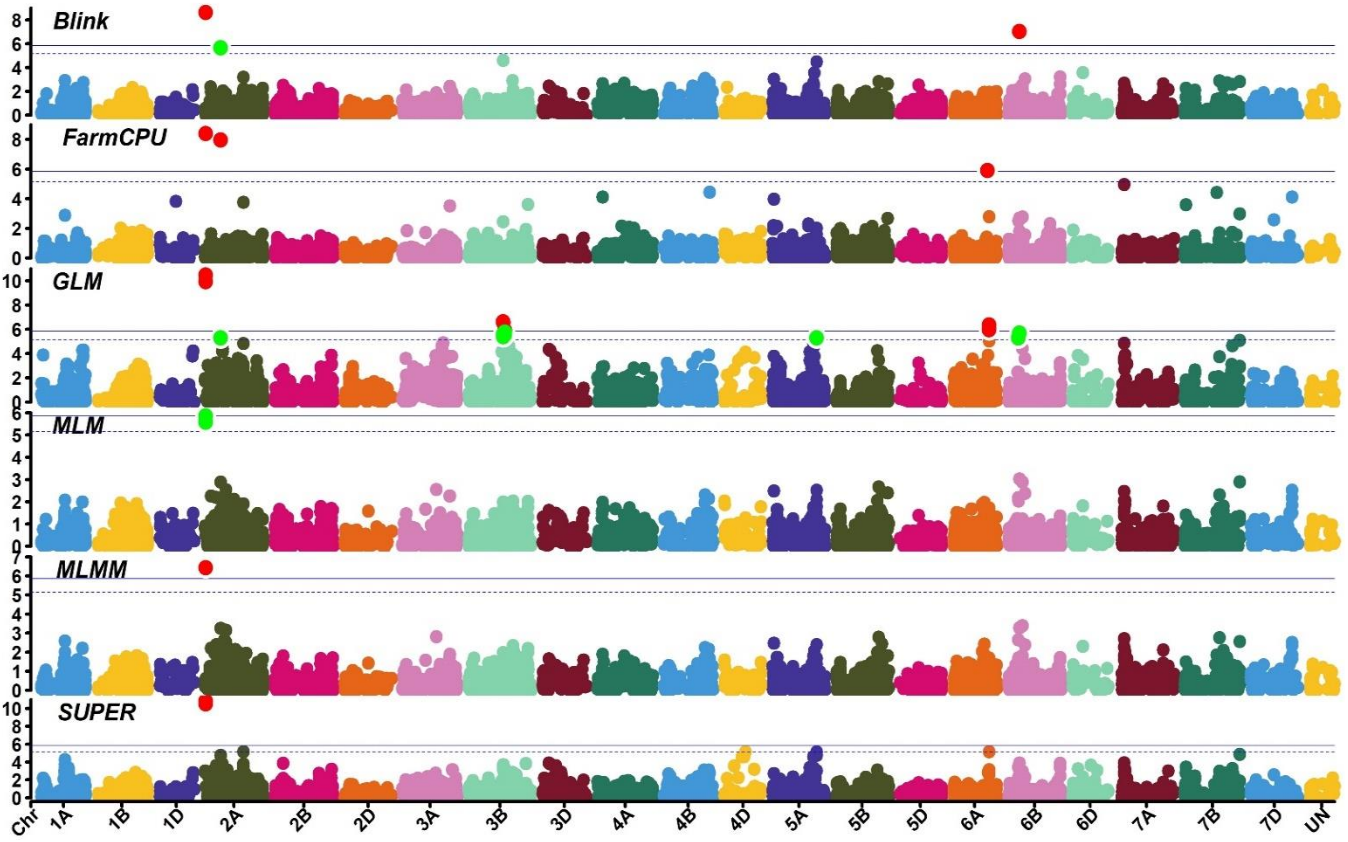

Jash19a

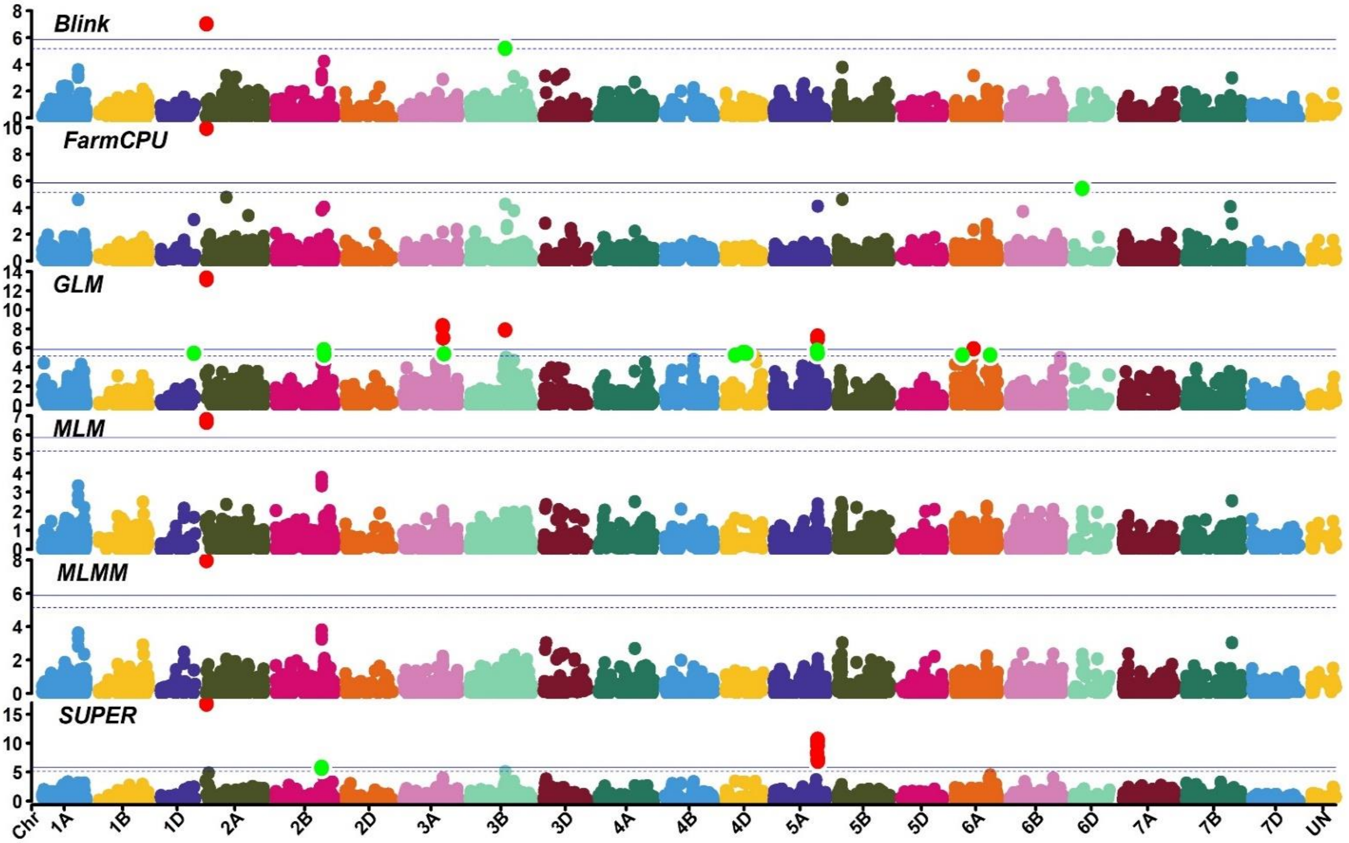

Jash19b

b) Jashore, Bangladesh 2020

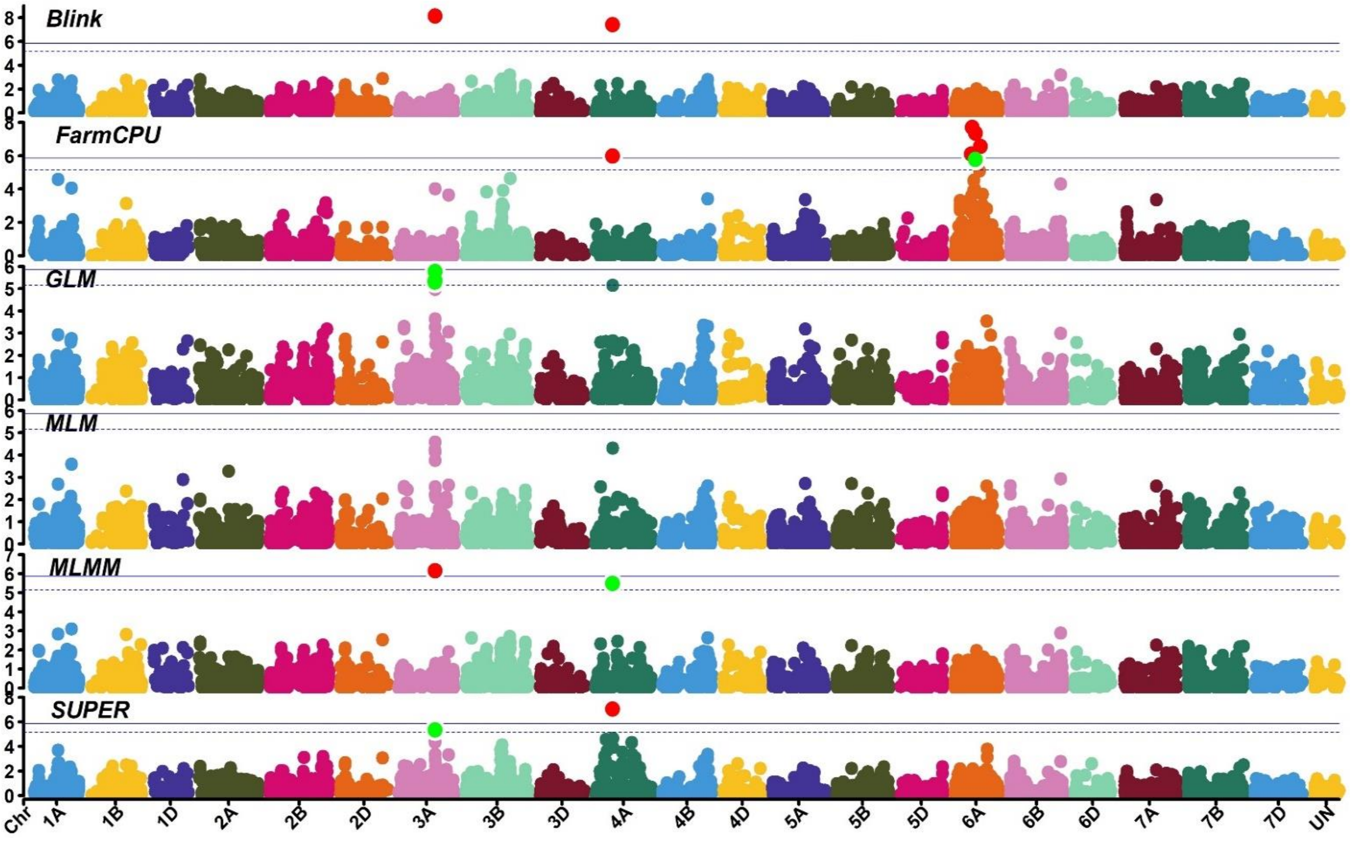

Jash20a

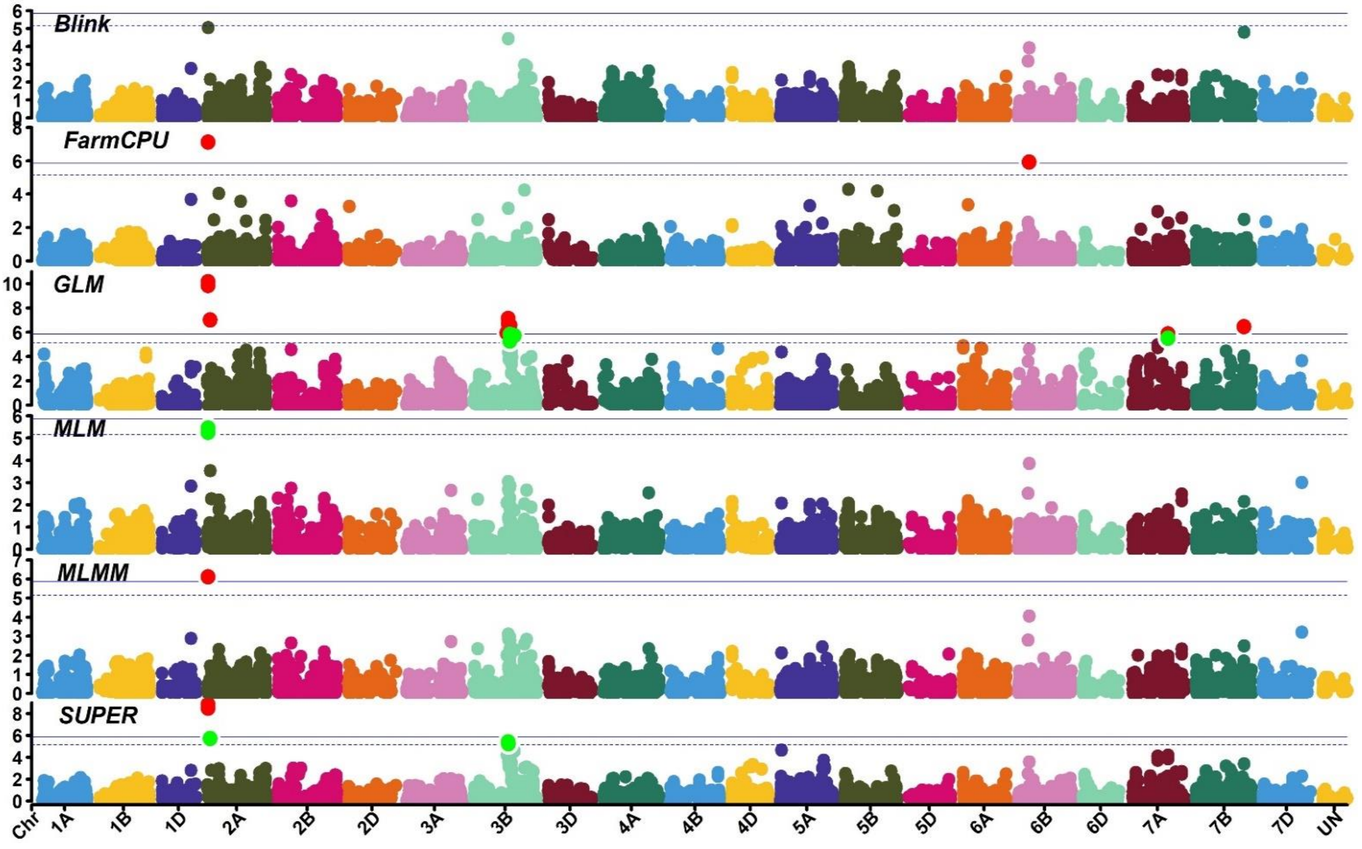

Jash20b

c) Jashore, Bangladesh 2021

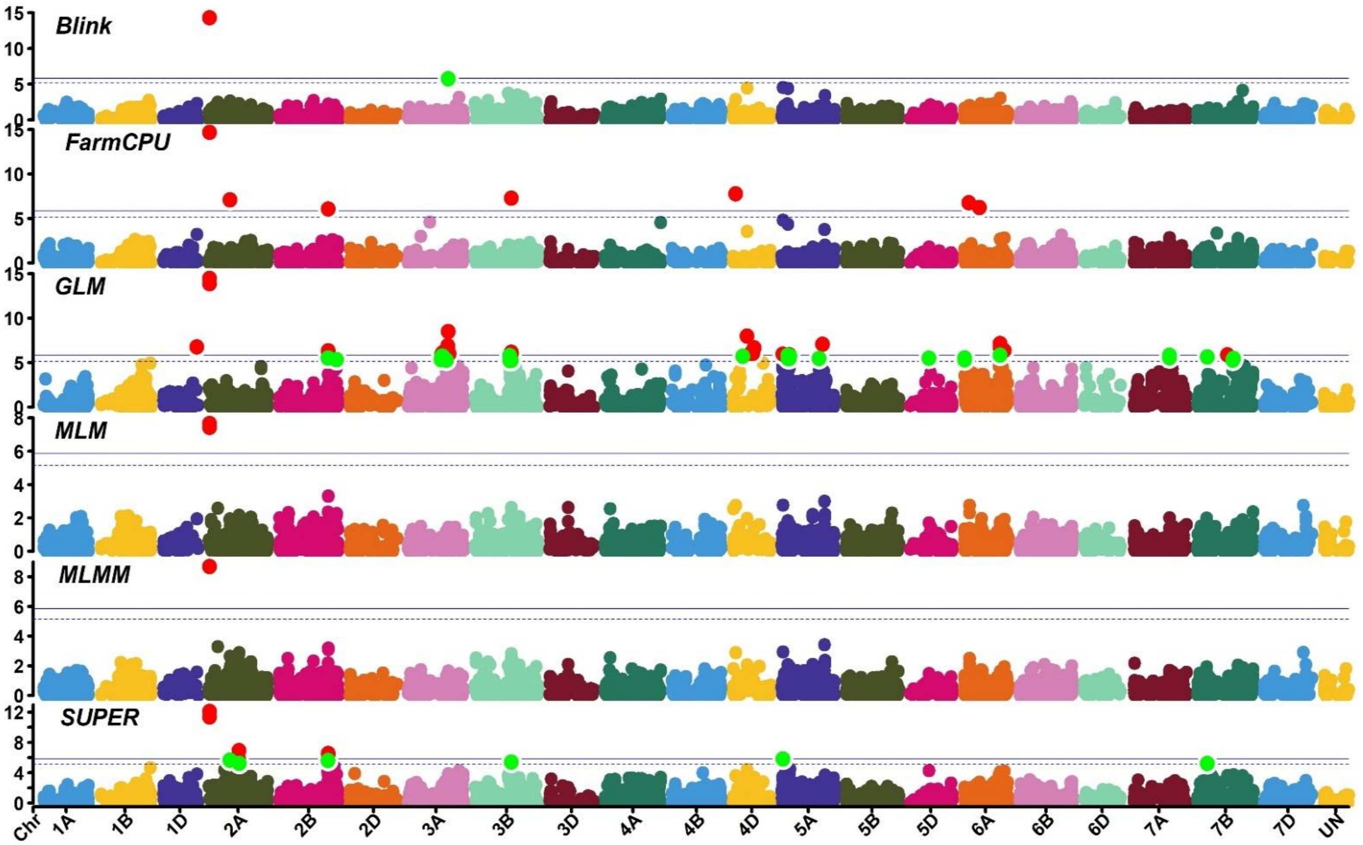

Jash21a

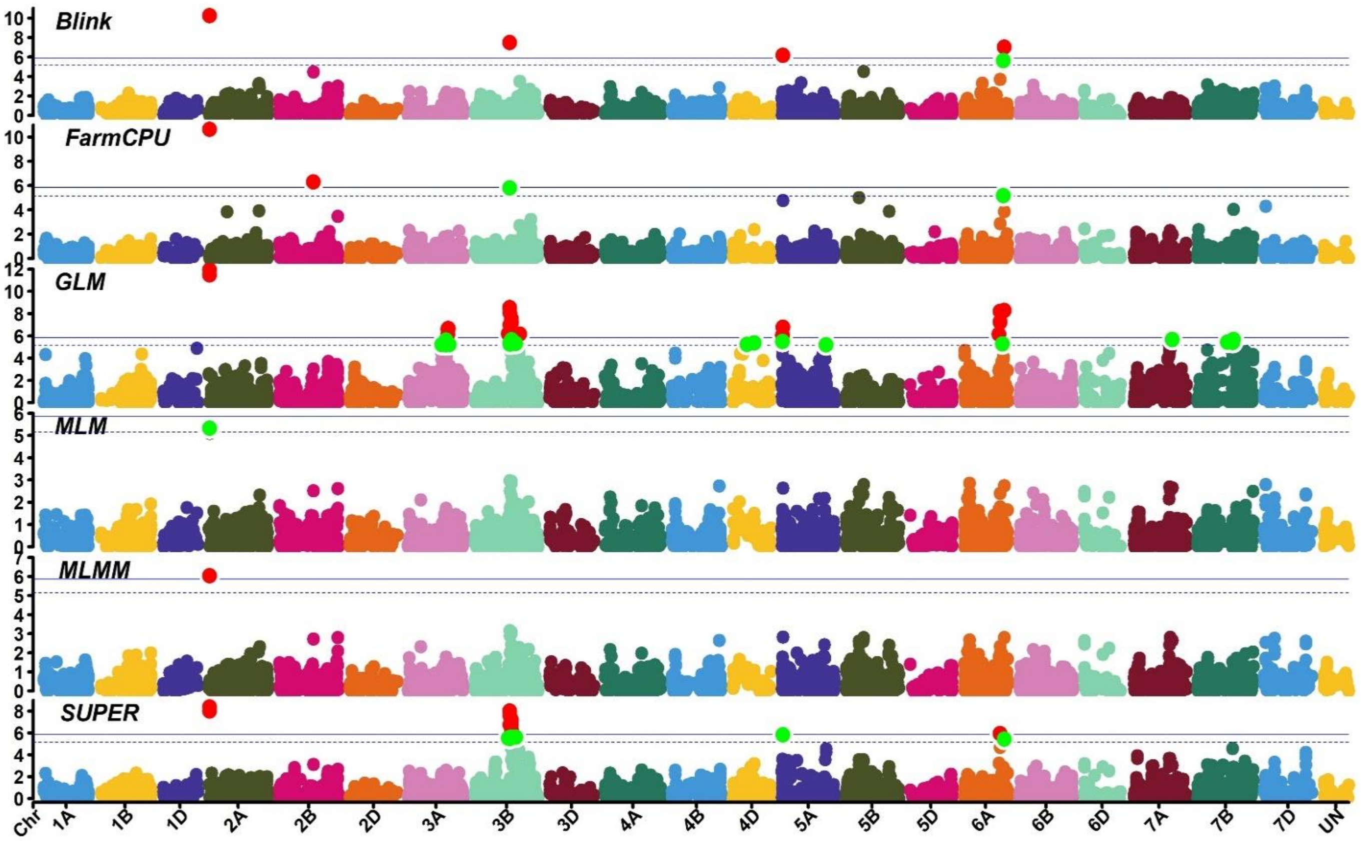

Jash21b

d) Okinawa, Bolivia, 2020

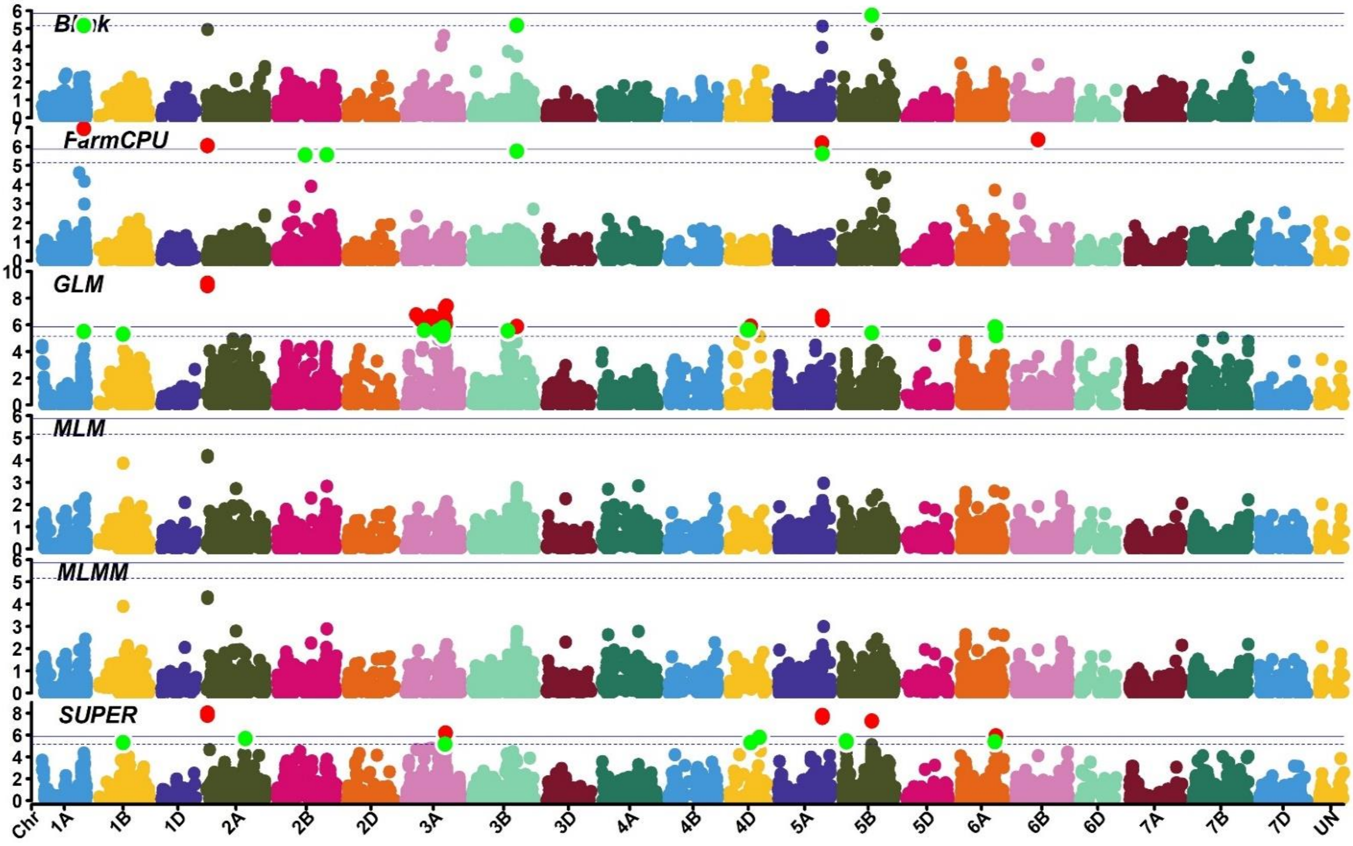

Okin20a

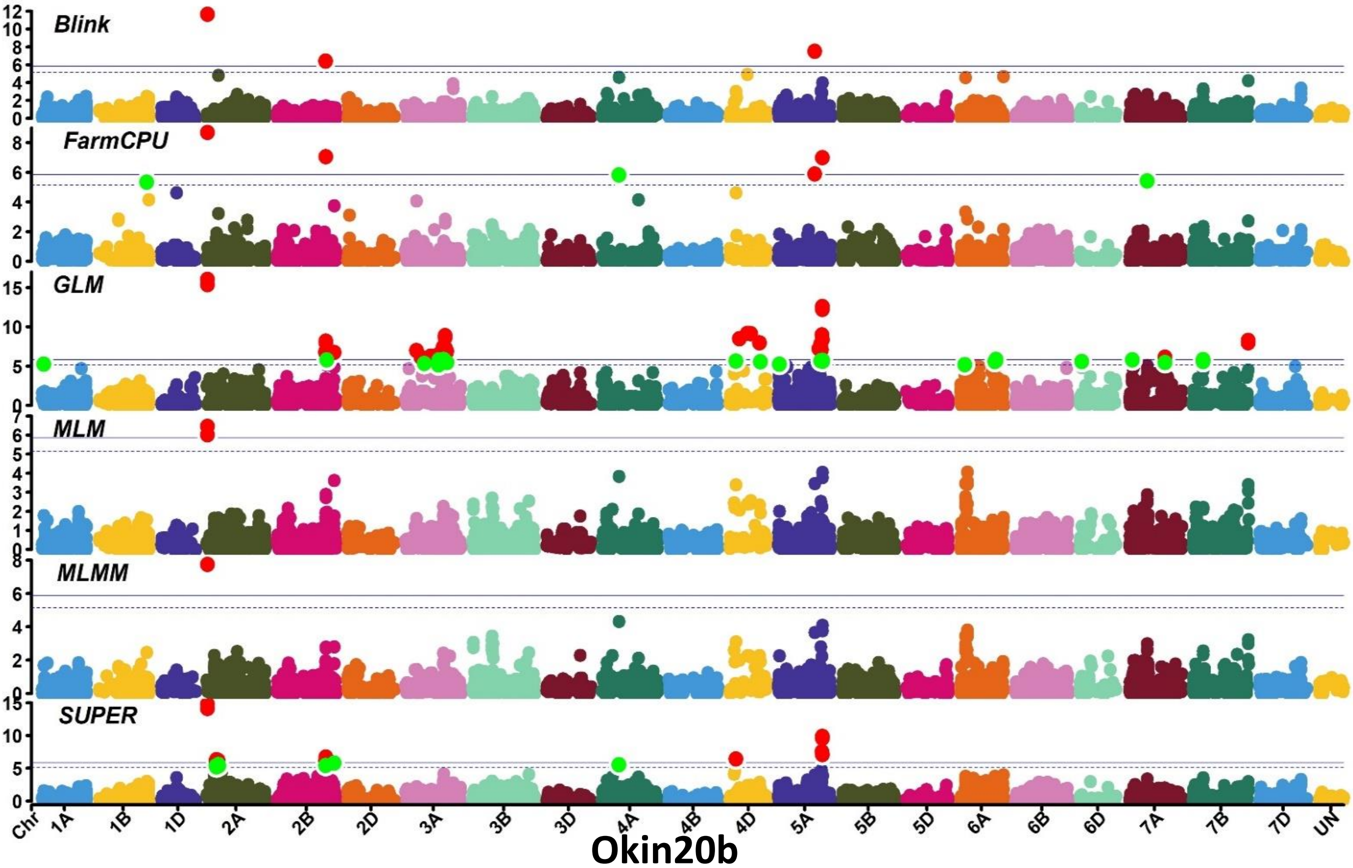

Okin20b

e) Okinawa, Bolivia, 2021

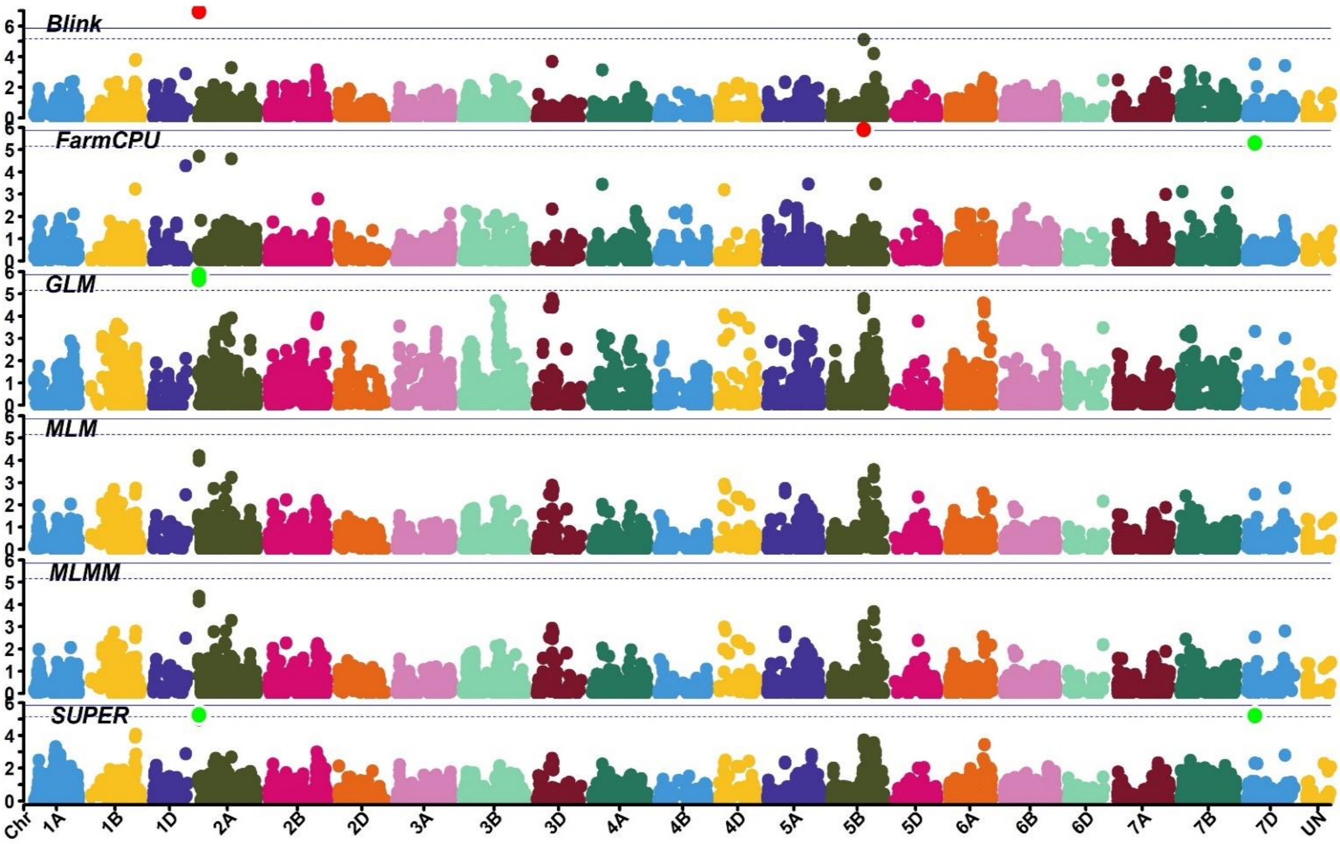

Okin21a

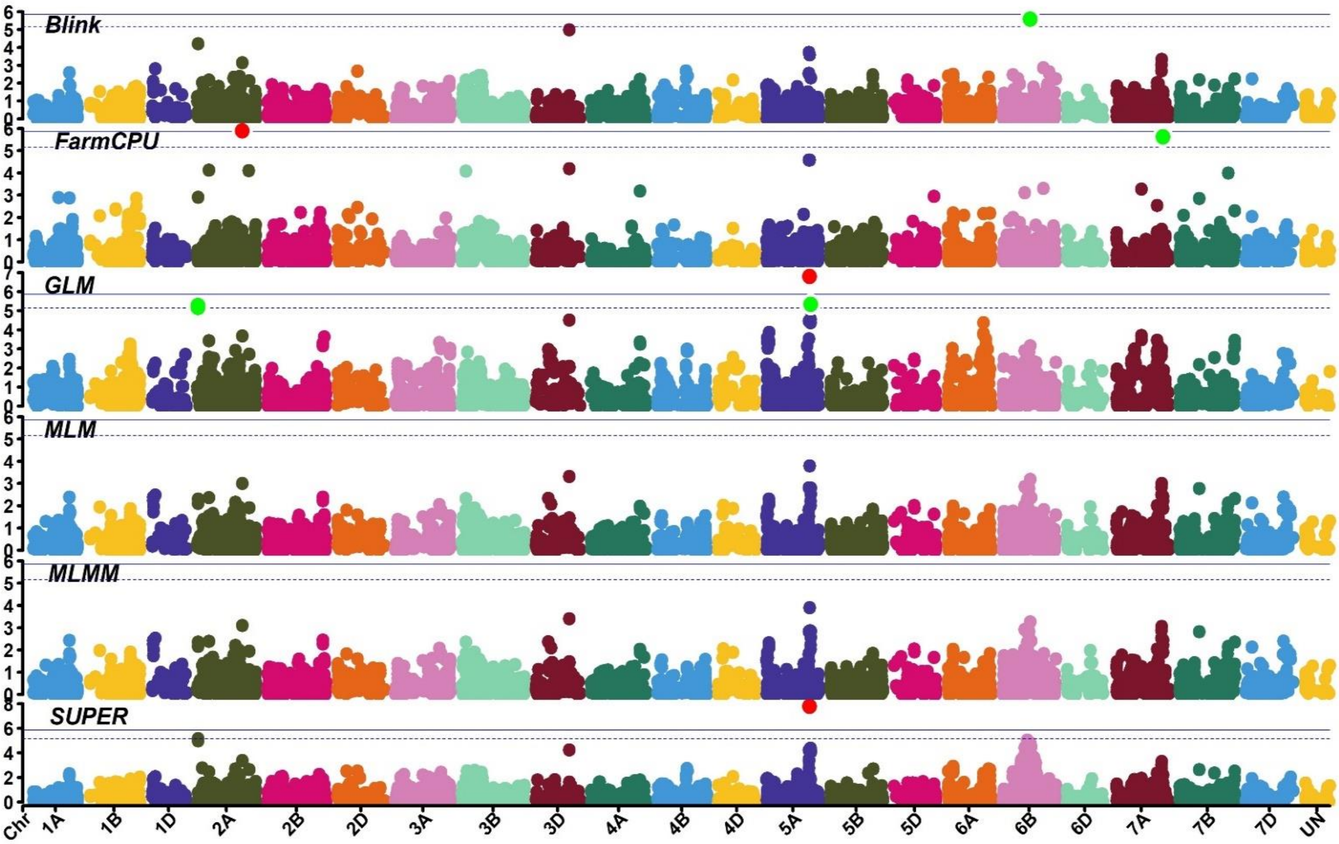

Okin21b

f) Quirusillas, Bolivia, 2020

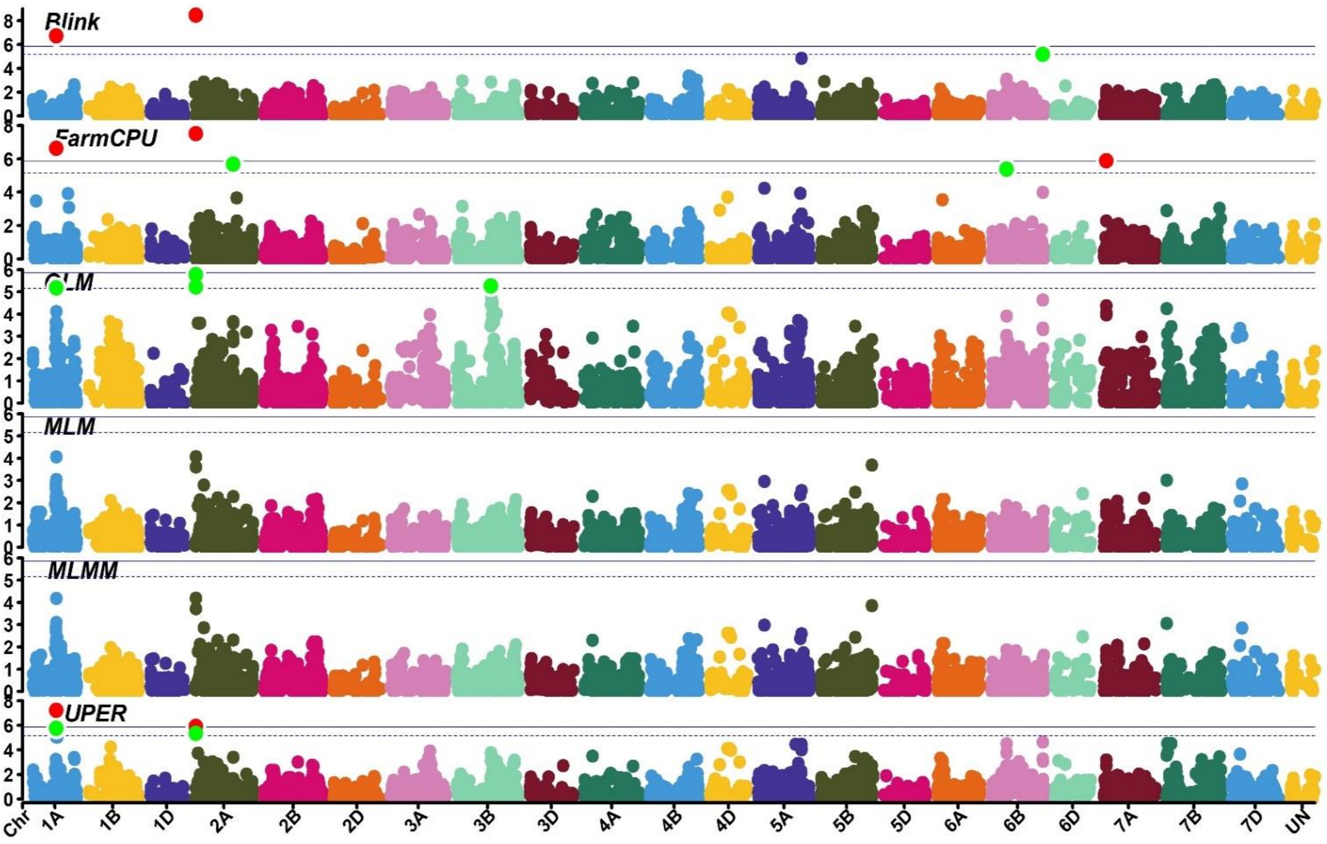

Quir20a

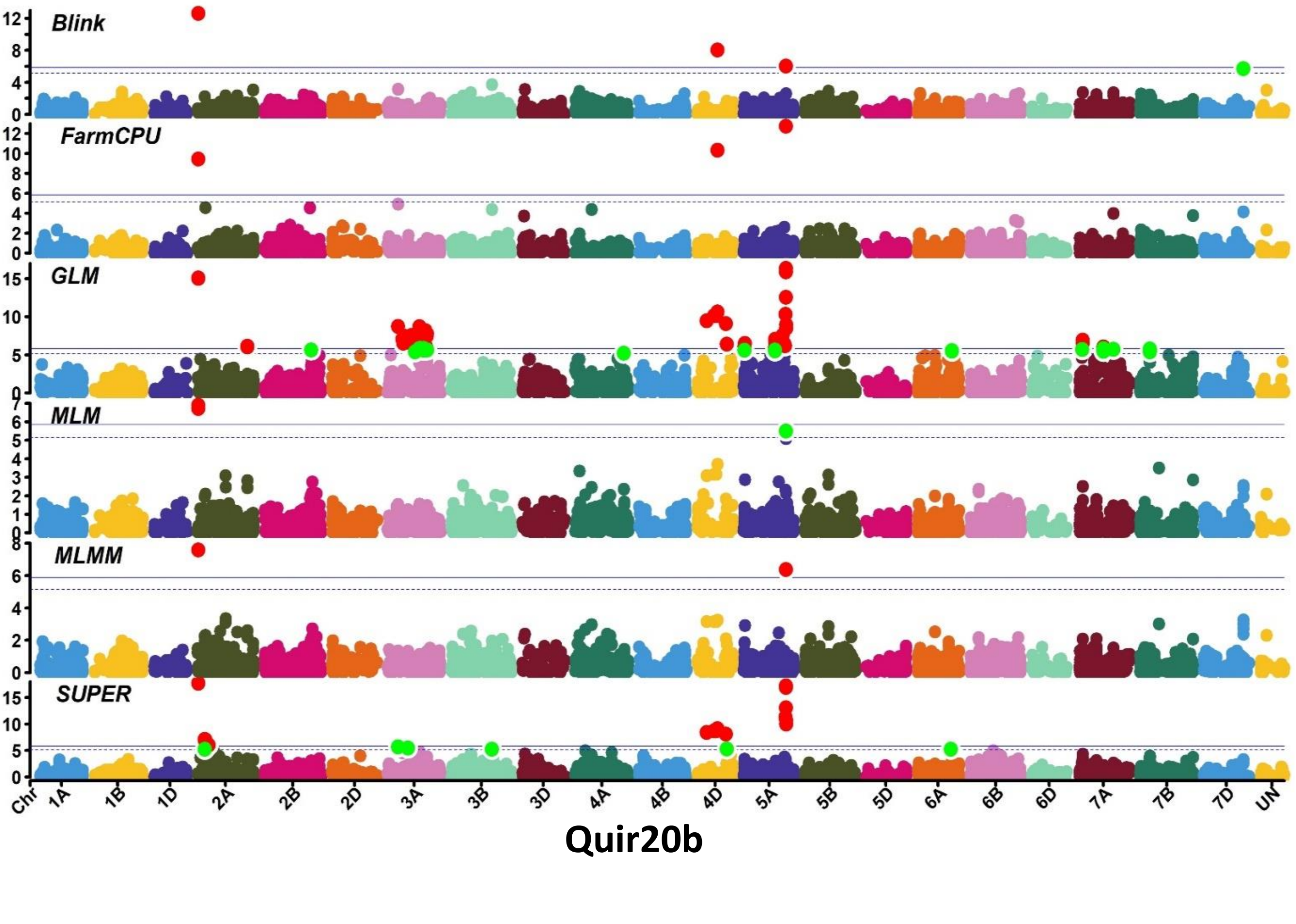

Quir20b

g) Quirusillas, Bolivia, 2021

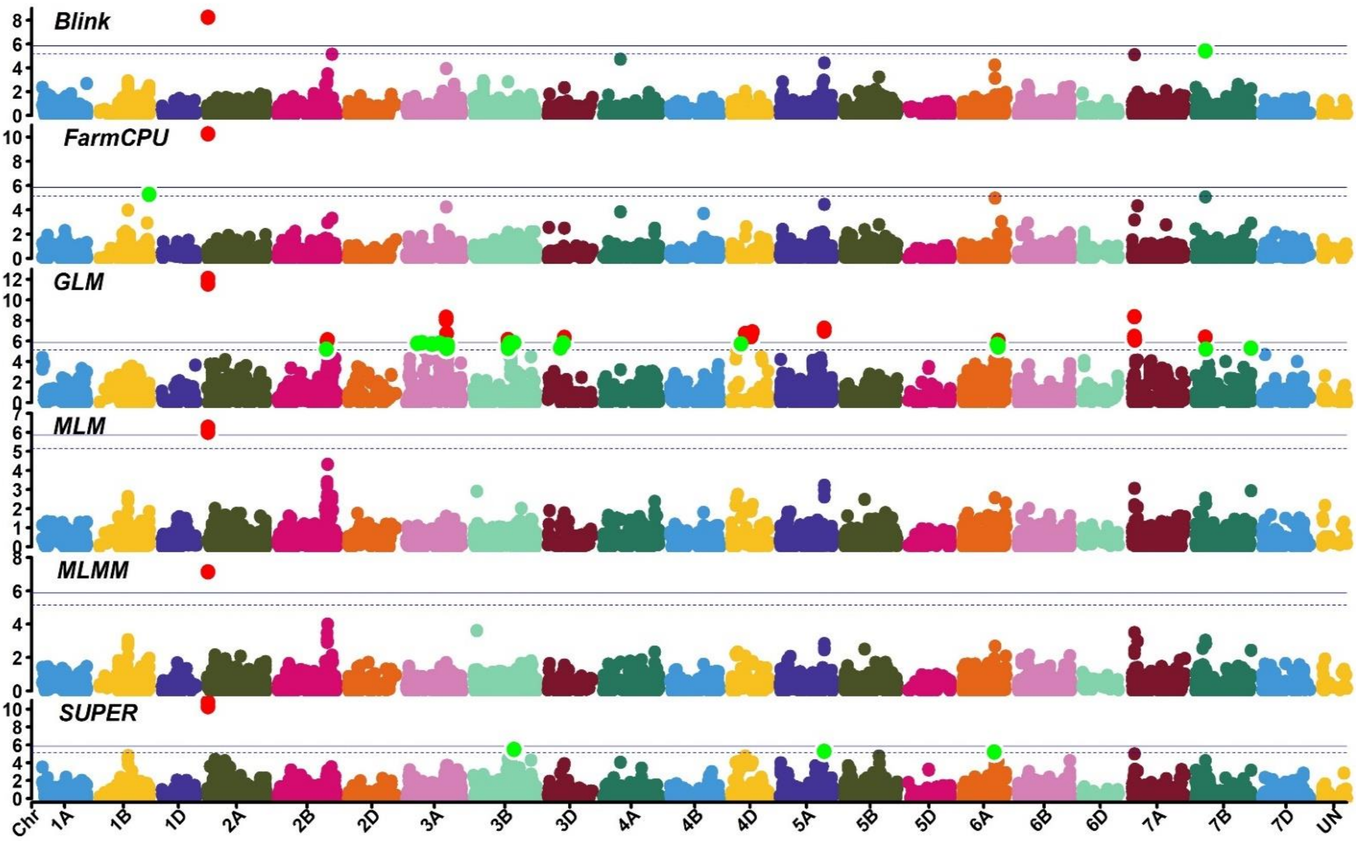

Quir21a

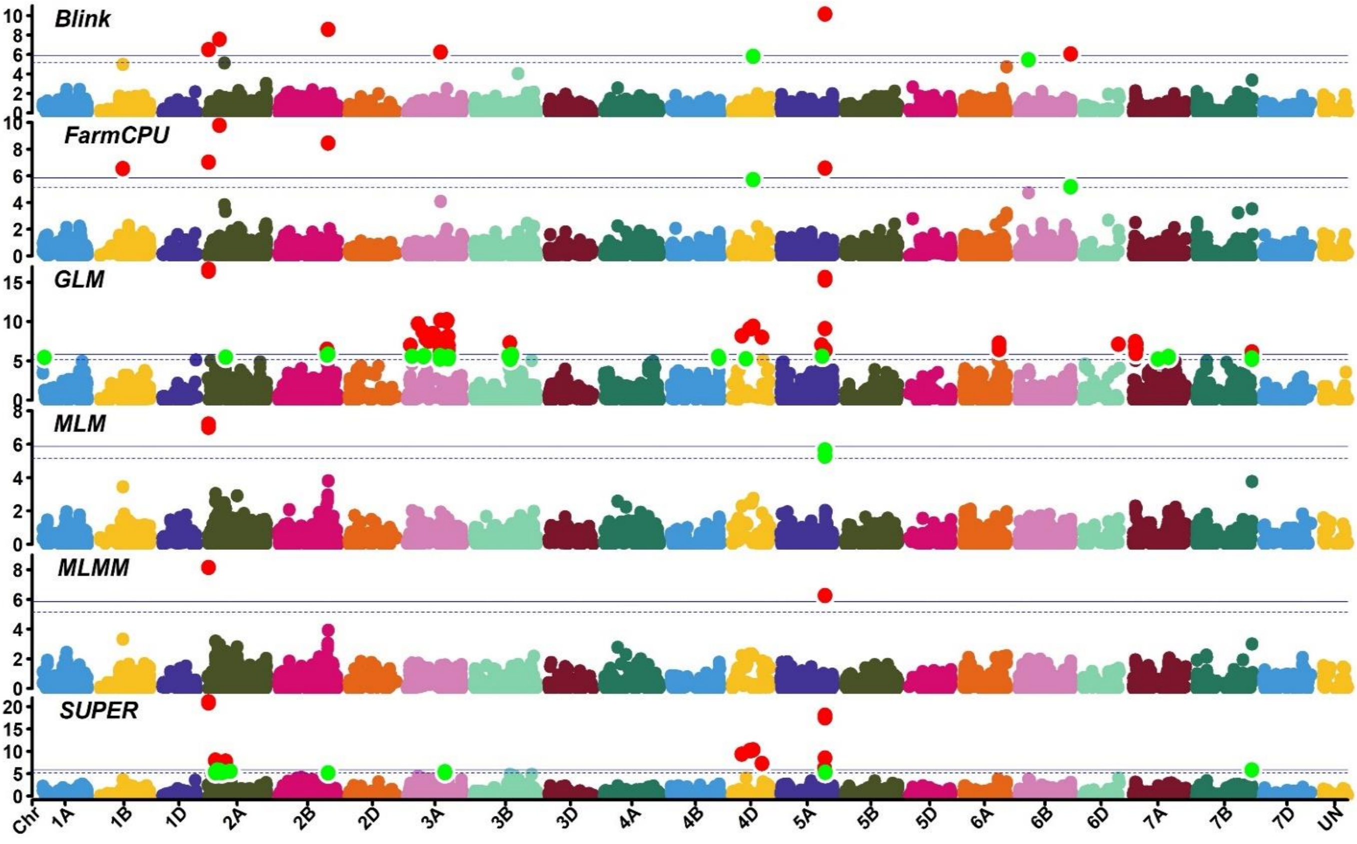

Quir21b
