# Supplementary material for: Field screening and genetic mapping of wheat blast resistance for a panel of common wheat from Bangladesh
Source: PLoS One. 2026 Jun 11;21(6):e0349201. doi: 10.1371/journal.pone.0349201 (PMC13258015; doi:10.1371/journal.pone.0349201)

S3 Fig. Variation in Linkage Disequilibrium across wheat chromosomes in the diverse bread wheat germplasm panel, visualized by r² and D' values. (*Red: high LD, Light Blue: low LD, White: no LD*)

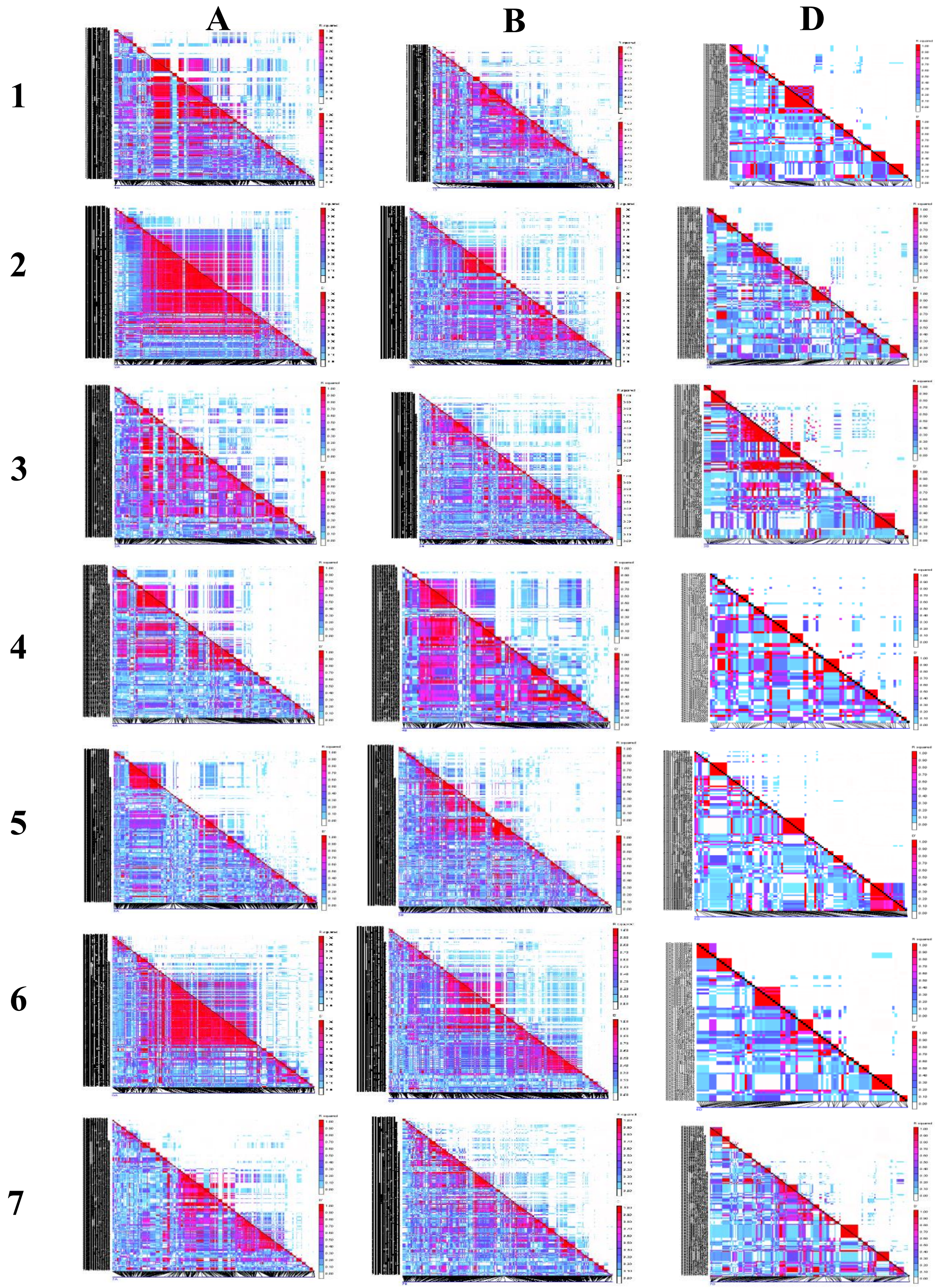

Supplement: S3 Fig — (PDF) [file pone.0349201.s003.pdf]
