# Supplementary material for: Field screening and genetic mapping of wheat blast resistance for a panel of common wheat from Bangladesh
Source: PLoS One. 2026 Jun 11;21(6):e0349201. doi: 10.1371/journal.pone.0349201 (PMC13258015; doi:10.1371/journal.pone.0349201)

S4 Fig. Genome-wide LD decay showing the decline of mean pairwise linkage disequilibrium (r^2) with increasing physical distance (Kb) and the estimated LD-decay distance (144 Kb).

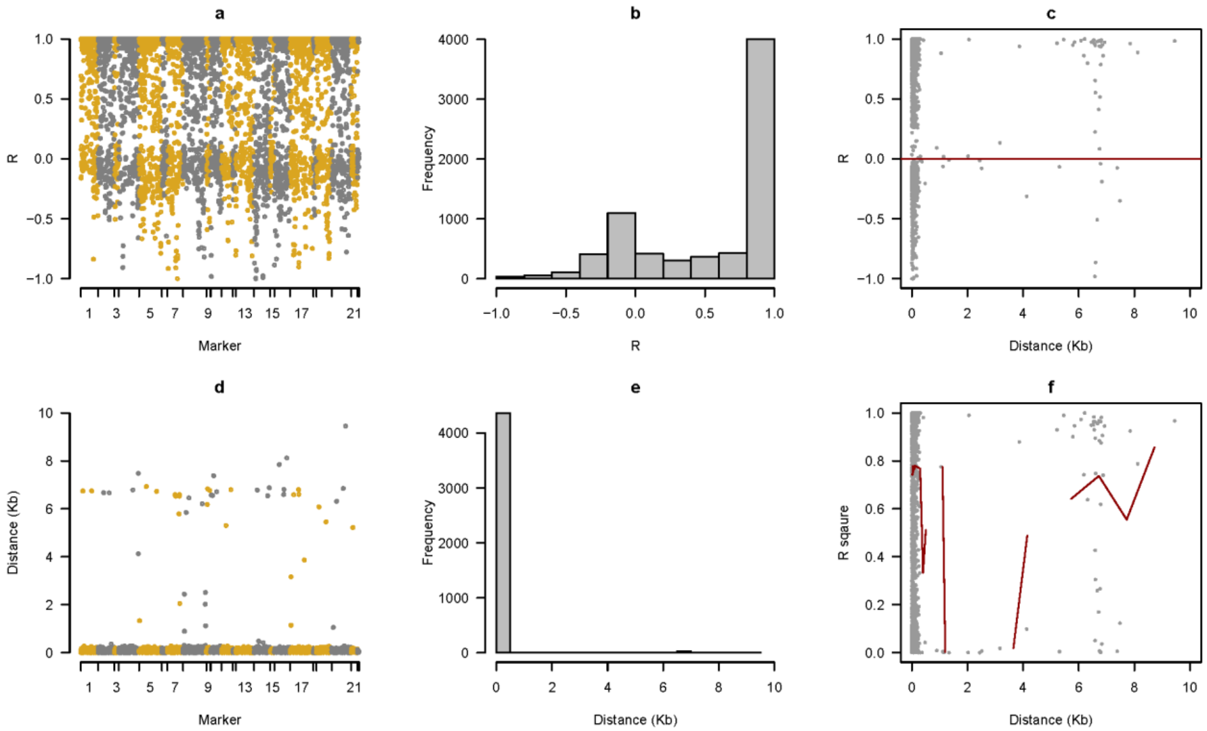

Supplement: S4 Fig — (PDF) [file pone.0349201.s004.pdf]
