# Supplementary material for: Field screening and genetic mapping of wheat blast resistance for a panel of common wheat from Bangladesh
Source: PLoS One. 2026 Jun 11;21(6):e0349201. doi: 10.1371/journal.pone.0349201 (PMC13258015; doi:10.1371/journal.pone.0349201)
Supplement: S1 Table — (PDF) [file pone.0349201.s005.pdf]

Table S1. Genotypes showing immune, resistant, or moderately resistant reactions across locations, along with their genetic composition for 2NS, Rmg1, Rmg6, and Rmg8. Genotype calls: "+" = homozygous presence, "–" = homozygous absence, "±" = heterozygous, "!" = no call or missing data.

SL	Entry	GID	CROSS/PEDIGREE<br>Selection history	Origin	2NS	Rwt3 (Rmg6)	Rwt4-B1 (Rmg1)	Rwt4-D1 (Rmg1)	Rmg8 (KM200)
**Immune Reaction (I)**									
1	28	5594927	NAC/TH.AC//3*PVN/3/MIRLO/BUC/4/2*PASTOR/5/KACHU/6/<br>KACHU	WBSN-4	+	–	–	+	–
2	34	5594661	WAXWING/4/SNI/TRAP#1/3/KAUZ*2/TRAP//KAUZ/5/TECUE #1	WBSN-10	+	–	!	+	–
3	35	5663692	WBLL1*2/CHAPIO/6/CNDO/R143//ENTE/MEXI75/3/AE.SQ/4/2*OCI/5<br>/2* CIRCUS/7/WBLL1*2/BRAMBLING	WBSN-11	+	–	–	+	–
4	67	7176604	KAUZ//ALTAR84/AOS/3/MILAN/KAUZ/4/SAUAL/5/SERI.1B//KAUZ/<br>HEVO/3/AMAD*2/4/KIRITATI<br>CMSS10Y00370S-099Y-099M-099NJ-099NJ-15WGY-0B	WBSN-43	+	–	–	+	–
5	77	7173680	WAXWING*2/KRONSTADF2004/3/TRCH/SRTU//KACHU/4/SAUAL/Y<br>ANAC//SAUAL<br>CMSS10B00811T-099TOPY-099M-099NJ-099NJ-15WGY-0B	WBSN-53	+	–	+	+	–
6	82	7175856	HW2045/3/WAXWING/SRTU//WAXWING/KIRITATI/4/KINGBIRD<br>#1//INQALAB 91*2/TUKURU<br>CMSS10B01183T-099TOPY-099M-099NJ-099NJ-18WGY-0B	WBSN-58	+	–	–	+	–
7	86	7178692	PASTOR//HXL7573/2*BAU/3/SOKOLL/WBLL1/4/SUNCO/2*PASTOR/<br>/EXC ALIBUR/5/W15.92/4/PASTOR//HXL7573/2*BAU/3/WBLL1<br>CMSA10M00253T-050Y-099ZTM-099NJ-099NJ-28WGY-0B	WBSN-62	+	–	–	+	–
8	140	7463264	BORLAUG #100 (BWMRI Gom 3)	WBSN-117	+	–	–	+	–
9	414	7047309	TUKURU//BAV92/RAYON/6/NG8201/KAUZ/4/SHA7//PRL/VEE#6/3/F<br>ASA_N/5/MILAN/KAUZ/7/SERI.1B//KAUZ/HEVO/3/AMAD*2/4/KIRI<br>TATI/8/AT TILA*2/PBW65*2//W485/HD29<br>CMSS10B00988T-099TOPY-099M-0SY-35M-0WGY	8th HLBSN-13	+	–	–	+	–

SL	Entry	GID	CROSS/PEDIGREE<br>Selection history	Origin	2NS	Rwt3<br>(Rmg6)	Rwt4-B1<br>(Rmg1)	Rwt4-D1<br>(Rmg1)	Rmg8<br>(KM200)
10	415	7175924	TRCH/SRTU//KACHU/3/CIRNO C 2008/4/BOKOTA<br>CMSS10B01218T-099TOPY-099M-099NJ-099NJ-11WGY-0B	8th HLBSN-16	+	−	−	+	−
11	418	7178871	WAXWING/KRONSTAD F2004//WHEAR/SOKOLL/3/WAXBI<br>CMSS10B00806T-099TOPY-099M-099NJ-099NJ-5RGY-0B	8th HLBSN-28	+	+	+	+	−
12	421	7177736	PRL/2*PASTOR/4/CHOIX/STAR/3/HE1/3*CNO79//2*SERI/8/NG8201/<br>KAUZ/4/SHA7//PRL/VEE#6/3/FASAN/5/MILAN/KAUZ/6/ACHYUTA/7<br>/PBW343 *2/KUKUNA/9/SUP152	8th HLBSN-32	+	+	−	+	−
13	446	7632196	BORL14*2//KFA/2*KACHU<br>CMSS12Y00770T-099TOPM-099Y-099M-0SY-51M-0WGY	12thSTEMRR<br>SN-6061	+	−	−	+	−
**Resistance Reaction(R)**									
1	15	6938713	PASTOR//HXL7573/2*BAU/3/ SOKOLL/WBLL1/4/...<br>CMSA09M00245T-050Y-050ZTM-0NJ-099NJ-11WGY-0B		+	+	−	+	−
2	33	5516069	ATTILA*2/PBW65*2//KACHU	WBSN-9	+	+	−	+	−
3	36	5594257	SAUAL #1/KACHU	WBSN-12	+	−	−	+	−
4	38	6342813	ATTILA/3*BCN//BAV92/3/TILHI/4/SUP152/5/SUP152<br>CMSS07Y00741T-099TOPM-099Y-099M-099Y-2M-0WGY	WBSN-14	+	−	−	+	−
5	53	6934561	HEILO//MILAN/MUNIA/8/2*NG8201/KAUZ/4/SHA7//PRL/VEE#6/3/F<br>ASAN/5/MILAN/KAUZ/6/ACHYUTA/7/PBW343*2/KUKUNA<br>CMSS10Y01240T-099TOPM-099Y-099M-28WGY-0B	WBSN-29	+	−	−	+	−
6	54	6935633	KACHU/SAUAL//KINGBIRD #1<br>CMSS09B00117S-099ZTM-099NJ-099NJ-4WGY-0B	WBSN-30	+	+	+	+	−
7	55	6689579	KENYA SUNBIRD/KACHU<br>CMSS08Y00235S-099Y-099M-099NJ-099NJ-9WGY-0B	WBSN-31	+	−	−	+	−
8	56	6934300	PFAU/MILAN/5/CHEN/AEGILOPS SQUARROSA (TAUS)//BCN<br>/3/VEE#7/BOW/4/PASTOR/6/PRL/SARA//TSI/VEE#5/3/TILHI/4/ATTIL<br>A/2*PASTOR/7/PBW343*2/KUKUNA//PBW343*2/KUKUNA<br>CMSS10Y01040T-099TOPM-099Y-099M-18WGY-0B	WBSN-32	+	+	−	+	−

SL	Entry	GID	CROSS/PEDIGREE<br>Selection history	Origin	2NS	Rwt3<br>(Rmg6)	Rwt4-B1<br>(Rmg1)	Rwt4-D1<br>(Rmg1)	Rmg8<br>(KM200)
9	68	7177302	TRCH/3/ROLF07/YANAC//TACUPETO<br>F2001/BRAMBLING/4/PRL/2*PASTOR<br>CMSS10Y00946T-099TOPM-099Y-099M-099NJ-099NJ-12WGY-0B	WBSN-44	+	+	−	+	−
10	78	7179806	KAUZ//ALTAR84/AOS/3/MILAN/KAUZ/4/SAUAL/5/PBW343*2/KUK<br>UNA//PARUS/3/PBW343*2/KUKUNA/6/KACHU/SAUAL<br>CMSS10B01028T-099TOPY-099M-099NJ-099NJ-14WGY-0B	WBSN-54	+	−	−	+	−
11	79	7174577	ATTILA/3*BCN//BAV92/3/TILHI/5/BAV92/3/PRL/SARA//TSI/VEE#5/4/<br>CR OC1/AE.SQUARROSA(224)//2*OPATA*2/6/TRCH/SRTU//KACHU<br>CMSS10B01048T-099TOPY-099M-099NJ-099NJ-11WGY-0B	WBSN-55	+	−	+	+	−
12	83	7175911	WBLL1*2/4/SNI/TRAP#1/3/KAUZ*2/TRAP//KAUZ/5/KACHU/6/CIRN<br>O C 2008/7/TACUPETO F2001/BRAMBLING*2//KACHU<br>CMSS10B01212T-099TOPY-099M-099NJ-099NJ-6WGY-0B	WBSN-59	+	−	−	+	−
13	84	7178596	W15.92/4/PASTOR//HXL7573/2*BAU/3/WBLL1/6/MTRWA92.161/PRI<br>NIA/5/SERI*3//RL6010/4*YR/3/PASTOR/4/BAV92<br>CMSA10M00616S-099ZTM-099NJ-099NJ-1WGY-0B	WBSN-60	+	−	−	+	−
14	88	7178891	KACHU/SAUAL/4/VARIS/MISR2,EGY/3/FRET2/KUKUNA//FRET2/5/<br>KACHU/SAUAL<br>CMSS10B01033T-099TOPY-099M-099NJ-099NJ-11RGY-0B	WBSN-64	+	−	−	+	−
15	92	7178889	KACHU/SAUAL/4/VARIS/MISR2,EGY/3/FRET2/KUKUNA//FRET2/5/<br>KACHU/SAUAL<br>CMSS10B01033T-099TOPY-099M-099NJ-099NJ-9RGY-0B	WBSN-70	+	−	−	+	−
16	94	6968692	KACHU/SAUAL/4/VARIS/MISR2,EGY/3/FRET2/KUKUNA//FRET2/5/<br>KACHU/SAUALY<br>CMSS10B01033T-099TOPY-099M-0SY-21M-0RG	WBSN-72	+	−	−	+	−
17	111	5516503	KACHU#1/4/CROC_1/AE.SQUARROSA(205)//KAUZ/3/SASIA/5/KAC<br>HU	WBSN-88	+	−	−	+	−
18	131	6932358	WHEAR/KUKUNA/3/C80.1/3*BATAVIA//2*WBLL1/8/VEE#8//JUP/BJ<br>Y/3/F3.71/TRM/4/BCN/5/KAUZ/6/MILAN/KAUZ/7/SKAUZ/PARUS//P<br>ARUS/9/K ACHU<br>CMSS09Y00764T-099TOPM-099Y-099M-099Y-1WGY-0B	WBSN-107	+	+	−	+	−
19	132	6934550	PFAU/MILAN/5/CHEN/AEGILOPS SQUARROSA<br>(TAUS)//BCN/3/VEE#7/BOW/4/PASTOR/8/2*SHA7//PRL/VEE#6/3/FAS<br>AN/4/HAAS8446/2*FASAN/5/CBRD/KAUZ/6/MILAN/AMSEL/7/FRET<br>2*2/KUK UNA<br>CMSS10Y01238T-099TOPM-099Y-099M-5WGY-0B	WBSN-108	+	+	−	+	−

SL	Entry	GID	CROSS/PEDIGREE<br>Selection history	Origin	2NS	Rwt3 (Rmg6)	Rwt4-B1 (Rmg1)	Rwt4-D1 (Rmg1)	Rmg8 (KM200)
20	134	5534344	PFAU/MILAN/5/CHEN/AEGILOPS SQUARROSA (TAUS)//BCN/3/VEE#7/BOW/4/PASTOR<br>CMSS02Y00613S-59Y-0M-099Y-5M-0WGY-0B	WBSN-110	+	+	−	+	−
21	136	7179794	KAUZ//ALTAR84/AOS/3/MILAN/KAUZ/4/SAUAL/5/SERI.1B//KAUZ/HEVO/3/AMAD*2/4/KIRITATI/6/KACHU/SAUAL<br>CMSS10B01027T-099TOPY-099M-099NJ-099NJ-9WGY-0B	WBSN-112	+	−	−	+	−
22	139	7398412	KFA/2*KACHU/5/UP2338*2/SHAMA/3/MILAN/KAUZ//CHIL/CHUM18/4/ UP2338*2/SHAMA/6/KFA/2*KACHU<br>CMSS11B00955T-099TOPY-099M-099NJ-099NJ-11WGY-0B	WBSN-116	+	−	−	+	−
23	141	7400585	BABAX/LR42//BABAX/3/ER2000/8/BOW/VEE/5/ND/VG9144//KAL/BB/3/ YACO/4/CHIL/6/CASKOR/3/CROC_1/AE.SQUARROSA (224)//OPATA/7/PASTOR//MILAN/KAUZ/3/BAV92<br>CMSA11Y00313S-099Y-099M-099NJ-099NJ-14WGY-0B	WBSN-118	+	−	−	+	−
24	210	296226	MILAN/SHA7<br>CM97550-0M-2Y-030H-3Y-3Y-0Y-1M-010Y-0FUS-015PR-0B	WBSN-185	+	−	+	+	−
25	411	7180142	BL 1724*2/3/T.DICOCCON PI272533/AE.SQUARROSA (458)//CMH81A.1261/VEE#10/4/2*UP2338*2/KKTS*2//YANA<br>CMSS11Y01293T-099TOPM-099Y-099M-5WGY-0B	8th HLBSN-3	+	−	−	+	!
26	412	7174077	PBW343*2/KUKUNA/5/KAUZ//ALTAR84/AOS/3/PASTOR/4/TILHI/6/PBW343/7/TUKURU//BAV92/RAYON/6/NG8201/KAUZ/4/SHA7//PRL/VEE#6/3/FASAN/5/MILAN/KAUZ/8/ATTILA*2//CHIL/BUC*2/3/KUKUNA<br>CMSS10B00961T-099TOPY-099M-099NJ-099NJ-15WGY-0B	8th HLBSN-7	+	+	−	+	−
27	416	7173723	CAL/NH//H567.71/3/SERI/4/CAL/NH//H567.71/5/2*KAUZ/6/WH576/7/WH542/8/WAXWING/9/ATTILA*2/PBW65//PIHA/3/ATTILA/2*PASTOR/1 0/UP2338*2/KKTS*2//YANAC<br>CMSS10B00824T-099TOPY-099M-099NJ-099NJ-19WGY-0B	8th HLBSN-19	+	−	−	+	−
28	419	7176817	PBW343*2/KUKUNA//PIHA/3/PBW343/7/TUKURU//BAV92/RAYON/6/NG8201/KAUZ/4/SHA7//PRL/VEE#6/3/FASAN/5/MILAN/KAUZ<br>CMSS10Y00503S-099Y-099M-099NJ-099NJ-1WGY-0B	8th HLBSN-30	+	−	−	+	!
29	422	7180156	T.SPELTAPI348599//2*PBW343*2/KUKUNA/3/WBLL1*2/KURUKU//HEILO<br>CMSS11Y00566S-099Y-099M-1RGY-0B	8th HLBSN-33	+	+	−	+	−
30	433	7310617	ND643/2*TRCH//MUTUS/3/SUP152/4/SUP152*2/TECUE #1<br>CMSS11B00486S-099M-0SY-27M-0WGY	9th HLBSN-20	+	−	−	+	−

SL	Entry	GID	CROSS/PEDIGREE<br>Selection history	Origin	2NS	Rwt3 (Rmg6)	Rwt4-B1 (Rmg1)	Rwt4-D1 (Rmg1)	Rmg8 (KM200)
31	442	7461841	MUTUS*2/KINGBIRD #1/3/KSW/SAUAL//SAUAL<br>CMSS11B00505S-099M-099NJ-099NJ-27WGY-0M	12thSTEMRR<br>SN-6009	+	−	−	+	−
32	445	7628792	SUP152/BAJ #1//KFA/2*KACHU<br>CMSS12Y00244S-099Y-099M-0SY-24M-0WGY	12thSTEMRR<br>SN-6032	+	−	−	+	−
33	447	7630056	KUTZ*2//KFA/2*KACHU<br>CMSS12Y00814T-099TOPM-099Y-099M-0SY-20M-0WGY	12thSTEMRR<br>SN-6065	+	−	−	+	−
34	449	7627645	ATTILA/3*BCN//BAV92/3/TILHI/4/SUP152/5/SUP152/6/KFA/2*KACH U/7/ATTILA/3*BCN//BAV92/3/PASTOR/4/TACUPETO F2001*2/BRAMBLING/5/PAURAQ	12thSTEMRR<br>SN-6101	+	−	−	+	−
**Moderately Resistance Reaction (MR)**									
1	3	6683088	SWSR22T.B./KACHU//2* KACHU<br>CMSS08Y01088T-099M-099Y-099M-099NJ-14WGY-0B		+	−	−	±	−
2	4	6565838	TIMBA/ELVIRA/3/BERKUT//PBW343*2/KUKUNA<br>CMSS08B00133S-099M-099Y-12M-0WGY		+	+	−	+	−
3	7	6730327	ATTILA/3*BCN/3/CROC_1/ AE. SQUARROSA (224)//...<br>CMSS09Y00860T-099TOPM-099Y-099ZTM-099NJ-099NJ...		+	−	−	+	−
4	71	7177669	YAYE/4/WAXWING/3/PFAU/WEAVER//BRAMBLING/5/KACHU/SAU AL<br>CMSS10Y01183T-099TOPM-099Y-099M-099NJ-099NJ-8WGY-0B	WBSN-47	+	+	−	+	−
5	93	7178839	WBLL1/KUKUNA//TACUPETOF2001/6/PVN//CAR422/ANA/5/BOW/C ROW//BUC/PVN/3/YR/4/TRAP#1/7/CNO79//PF70354/MUS/3/PASTOR/ 4/B AV92*2/5/FH6-1-7<br>CMSS10B00547S-099M-099NJ-099NJ-6RGY-0B	WBSN-71	+	+	+	+	−
6	186	7025946	HD 2967	WBSN-162	+	+	−	−	−
7	408	7178485	GLADIUS/5/2*W15.92/4/PASTOR//HXL7573/2*BAU/3/WBLL1<br>CMSA10M00177T-050Y-099ZTM-099NJ-099NJ-3WGY-0B	WBSN-384	+	−	−	+	−
8	420	8916011	BARI Triticale 1	S. MULTI	+	−	−	!	−

SL	Entry	GID	**CROSS/PEDIGREE**<br>**Selection history**	Origin	2NS	Rwt3 (Rmg6)	Rwt4-B1 (Rmg1)	Rwt4-D1 (Rmg1)	Rmg8 (KM200)
9	423	7170965	BOKOTA/3/UP2338*2/KKTS*2//YANAC<br>CMSS10B00295S-099M-099NJ-099NJ-20WGY-0B	8th HLBSN-40	+	–	–	+	–
10	427	7176732	47.FRET2*2/KUKUNA//PVN/3/FRET2*2/SHAMA/4/TRCH/SRTU//KACHU<br>CMSS10Y00446S-099Y-099M-099NJ-099NJ-28WGY-0B	8th HLBSN-47	+	±	–	+	–
11	428	7399831	UP2338*2/SHAMA/3/MILAN/KAUZ//CHIL/CHUM18/4/UP2338*2/SHAMA*2/5/PBW343*2/KUKUNA*2//FRTL/PIFED<br>CMSS11Y00993T-099TOPM-099Y-099M-099NJ-099NJ-4WGY-0B	9th HLBSN-6	+	±	–	+	–
12	429	7400087	SAUAL/3/SW89.3064//CMH82.17/SERI/4/SAUAL/5/PBW343*2/KUKUNA* 2//FRTL/PIFED/6/SAUAL/KRONSTAD F2004<br>CMSS11Y01091T-099TOPM-099Y-099M-099NJ-099NJ-16WGY-0B	9th HLBSN-8	+	+	–	+	–
13	432	7310275	BORL14//KFA/2*KACHU<br>CMSS11B00167S-099M-0SY-2M-0WGY	9th HLBSN-16	+	–	–	+	–
14	434	7400724	MUNAL#1/7/CNO79//PF70354/MUS/3/PASTOR/4/BAV92/5/FRET2/KUKUNA//FRET2/6/MILAN/KAUZ//PRINIA/3/BAV92<br>CMSA11Y00449S-099Y-099M-099NJ-099NJ-13WGY-0B	9th HLBSN-32	+	–	–	+	–
15	435	7400738	WHEAR/SOKOLL/8/BOW/VEE/5/ND/VG9144//KAL/BB/3/YACO/4/CHIL/6/CASKOR/3/CROC_1/AE.SQUARROSA(224)//OPATA/7/PASTOR//MILAN/ KAUZ/3/BAV92<br>CMSA11Y00467S-099Y-099M-099NJ-099NJ-12WGY-0B	9th HLBSN-33	+	–	–	+	–
16	441	7461788	MUTUS*2/KINGBIRD #1/3/KSW/SAUAL//SAUAL<br>CMSS11B00495S-099M-099NJ-099NJ-11WGY-0M	12thSTEMRR SN-6007	+	+	–	+	–
17	443	7631628	BORL14//KFA/2*KACHU<br>CMSS12Y00069S-099Y-099M-0SY-1M-0WGY	12thSTEMRR SN-6019	+	–	–	+	–
18	444	7628787	SUP152/BAJ #1//KFA/2*KACHU<br>CMSS12Y00244S-099Y-099M-0SY-14M-0WGY	12thSTEMRR SN-6031	+	–	–	+	–

"+" = Homozygous presence of the gene (positive call); "–" = Homozygous absence of the gene (negative call); "±" = Heterozygous genotype; "!" = No call or missing genotype data.
