# Supplementary material for: Field screening and genetic mapping of wheat blast resistance for a panel of common wheat from Bangladesh
Source: PLoS One. 2026 Jun 11;21(6):e0349201. doi: 10.1371/journal.pone.0349201 (PMC13258015; doi:10.1371/journal.pone.0349201)
Supplement: S1 File — (PDF) [file pone.0349201.s006.pdf]

File S1: GWAS Methods in GAPIT to Identify SNPs for Wheat Blast Resistance:

A Genome-Wide Association Study (GWAS) is like a big detective project where we look at the "recipes" or genetic instructions of many different wheat germplasms. We compare these instructions with how each plant actually performs in the field to resists disease or other causal effects.

By finding common patterns in the genetic instructions of plants that share a specific trait (like disease resistance), we can identify which parts of the genetic instruction are important for that trait. This helps plant breeders develop new wheat varieties that have these beneficial traits, leading to healthier and more productive crops.

In our study we use GAPIT (J. Wang & Zhang, 2021), an R package, which offers various statistical methods for conducting GWAS analysis. The following methods we used in our analysis:

- **General Linear Model (GLM):** This is the simplest approach, directly testing the association between a phenotype and each marker individually.

  The model equation is:

  $y = \beta_0 + \beta_1 S_1 + \varepsilon$

  where: y is the phenotype, β_0 is the intercept, β_1 is the effect size of marker S_1, S_1 is the genotype score for marker 1 (coded as 0, 1, or 2 for the number of minor alleles) and ε is the random error term. However, it can be susceptible to confounding factors like population structure(Myers & Montgomery, 1997).

- **Mixed Linear Model (MLM):** This method addresses the limitations of GLM by incorporating additional factors as random effects. One common approach is to include a kinship matrix (K) that captures the genetic relatedness between individuals.

The model equation is: $y = \beta_0 + \beta_1 S_1 + Zu + \varepsilon$. where, all terms from GLM are included (y, β_0, β_1, S_1, ε), Z is the design matrix relating individuals to random effects, u is a vector of random polygenic effects following a multivariate normal distribution with mean 0 and variance structure defined by the kinship matrix (K)(Zhang et al., 2010). This model helps control for false positives arising from population structure and cryptic relatedness. However, MLM can lead to underestimating the effect of markers due to confounding between K and the markers themselves.

- **Multiple Loci Mixed Model (MLMM):** This method refines the MLM approach by iteratively including previously identified associated markers (S_j, where $j \neq 1$) as fixed effects in the model. The base model equation from MLM is modified to incorporate additional β coefficients and genotype scores (S_j) for each included marker (Segura et al., 2012). This allows the model to account for the effects of these markers while simultaneously testing for the association of the focal marker (S_1).

  Here's a general representation of the model with two additional markers (S_2 and S_3) included as covariates: $y = \beta_0 + \beta_1 S_1 + \beta_2 S_2 + \beta_3 S_3 + Zu + \varepsilon$. However, the kinship matrix (K) remains unchanged, potentially leading to residual confounding.

- **Settlement of Mixed Linear Models Under Progressively Exclusive Relationship (SUPER):** This method addresses the confounding issue in MLM by creating a refined kinship matrix (K) that excludes the influence of markers in strong linkage disequilibrium (LD) with the tested marker (S_1). The standard MLM model equation ($y = \beta_0 + \beta_1 S_1 + Zu + \varepsilon$) is still used, but the key difference lies in the construction of the Z matrix (Q. Wang et al., 2014). In SUPER, the kinship matrix (K) used in Z is modified to account for LD between markers. Markers that are highly correlated with the tested marker (S_1) are down-weighted or even excluded from the kinship calculation. This

reduces the influence of these markers on the random polygenic effects (u) and helps to isolate the specific effect of S_1 on the phenotype (y).

- **Fixed and random model Circulating Probability Unification (FarmCPU):** This method takes a fundamentally different approach compared to MLM and MLMM by forgoing the use of a kinship matrix (K) altogether (Liu et al., 2016). FarmCPU instead relies on a fixed-effect model framework to identify markers associated with the phenotype. The model equation itself is not directly represented due to the underlying statistical complexity. However, the core idea of FarmCPU involves using a likelihood-based approach to iteratively evaluate the association between each marker and the phenotype while accounting for the effects of other markers already included in the model. This iterative process helps to identify markers with genuine associations while reducing the influence of confounding factors.
- **Bayesian-information and Linkage-disequilibrium Iteratively Nested Keyway (BLINK):** This method leverages linkage disequilibrium (LD) information to improve statistical power(Huang et al., 2018). It iteratively removes markers in strong LD with the most significantly associated marker, effectively focusing on independent signals. Unlike FarmCPU, BLINK uses a fixed-effect model with Bayesian Information Criterion (BIC) to approximate the computationally expensive likelihood calculations in FarmCPU. Additionally, BLINK incorporates principal components and associated markers as covariates to further reduce false positives and negatives.

References:

Huang, M., Liu, X., Zhou, Y., Summers, R. M., Zhang, Z., & Hall, J. (2018). BLINK: A package for the next level of genome-wide association studies with both individuals and markers in the millions. *GigaScience*, *8*(2), 1–12.

https://doi.org/10.1093/gigascience/giy154

Liu, X., Huang, M., Fan, B., Buckler, E. S., & Zhang, Z. (2016). Iterative Usage of Fixed and Random Effect Models for Powerful and Efficient Genome-Wide Association Studies. *PLOS Genetics*, *12*(2), e1005767. https://doi.org/10.1371/journal.pgen.1005767

Myers, R. H., & Montgomery, D. C. (1997). A tutorial on generalized linear models. *Journal of Quality Technology*, *29*(3), 274–291. https://doi.org/10.1080/00224065.1997.11979769

Segura, V., Vilhjálmsson, B. J., Platt, A., Korte, A., Seren, Ü., Long, Q., & Nordborg, M. (2012). An efficient multi-locus mixed-model approach for genome-wide association studies in structured populations. *Nature Genetics*, *44*(7), 825–830. https://doi.org/10.1038/ng.2314

Wang, J., & Zhang, Z. (2021). GAPIT Version 3: Boosting Power and Accuracy for Genomic Association and Prediction. *Genomics, Proteomics and Bioinformatics*, *19*(4), 629–640. https://doi.org/10.1016/j.gpb.2021.08.005

Wang, Q., Tian, F., Pan, Y., Buckler, E. S., & Zhang, Z. (2014). A SUPER powerful method for genome wide association study. *PLoS ONE*, *9*(9). https://doi.org/10.1371/journal.pone.0107684

Zhang, Z., Ersoz1, E., Lai, C.-Q. Q., Todhunter, R. J., Tiwari, H. K., Gore, M. A., Bradbury, P. J., Yu, J., Arnett, D. K., Ordovas, J. M., Buckler, E. S., Ersoz, E., Lai, C.-Q. Q., Todhunter, R. J., Tiwari, H. K., Gore, M. A., Bradbury, P. J., Yu, J., Arnett, D. K., … Buckler, E. S. (2010). Mixed linear model approach adapted for genome-wide association studies. *Nature Genetics*, *42*(4), 335–360. https://doi.org/10.1038/ng.546
