# Supplementary material for: Field screening and genetic mapping of wheat blast resistance for a panel of common wheat from Bangladesh
Source: PLoS One. 2026 Jun 11;21(6):e0349201. doi: 10.1371/journal.pone.0349201 (PMC13258015; doi:10.1371/journal.pone.0349201)
Supplement: Attachment — Submitted filename: PONE-D-25-52203.pdf [file pone.0349201.s007.pdf]

PLOS ONE

Field screening and genetic mapping of wheat blast resistance for a panel of common wheat from Bangladesh

--Manuscript Draft--

**Manuscript Number:**	PONE-D-25-52203
**Article Type:**	Research Article
**Full Title:**	Field screening and genetic mapping of wheat blast resistance for a panel of common wheat from Bangladesh
**Short Title:**	Wheat Blast Resistance
**Corresponding Author:**	Pawan K. Singh<br>International Maize and Wheat Improvement Center (CIMMYT)<br>Texcoco, Mexico DF MEXICO
**Keywords:**	Wheat Blast, GWAS, Linkage Disequilibrium, SNP, QTL
**Abstract:**	Wheat blast, caused by Magnaporthe oryzae pathotype Triticum (MoT), is a rapidly emerging disease threatening wheat production in South Asia and beyond. To identify novel sources of resistance and dissect the underlying genetic architecture, a genome-wide association study (GWAS) was conducted using 432 diverse Bangladeshi wheat genotypes phenotyped across 14 multi-environment trials. Disease severity varied widely, with most genotypes exhibiting high susceptibility under favourable environments, although a few showed consistent resistance across locations. Genotyping of the wheat panel using ddRAD-seq revealed moderate population structure and diverse resistance allele distribution. GWAS using six different models identified a total of 1,121 significant SNPs distributed across multiple chromosomes. The 2NS translocation and known resistance genes (Rmg8, Rwt3, Rwt4-1B, Rwt4-1D) were unevenly distributed across the panel. Based on linkage disequilibrium (LD), the significant markers were grouped into 45 quantitative trait loci (QTL) associated with wheat blast resistance, including the 2NS/2AS translocation. These QTLs either coincide with known blast resistance loci or represent potentially novel ones. These findings enhance the understanding of wheat blast resistance and provide valuable markers and candidate genotypes for resistance breeding aimed to mitigate the threat of wheat blast under changing climates.
**Order of Authors:**	Md. Farhad, PhD
	Xinyao He, PhD
	Hirokazu Handa, PhD
	Mohammed Rezaul Kabir, PhD
	Krishna Kanta Roy, MSc
	Felix Marza, PhD
	Masahiro Kishi, PhD
	Soichiro Asuke, PhD
	Pawan K. Singh
**Additional Information:**	
**Question**	**Response**
**Financial Disclosure**<br><br>Enter a financial disclosure statement that describes the sources of funding for the work included in this submission. Review the submission guidelines for detailed requirements. View published research	Yes

articles from *PLOS ONE* for specific examples.<br><br>This statement is required for submission and **will appear in the published article** if the submission is accepted. Please make sure it is accurate.<br><br>**Funded studies**<br>Enter a statement with the following details:<br>• Initials of the authors who received each award<br>• Grant numbers awarded to each author<br>• The full name of each funder<br>• URL of each funder website<br>• Did the sponsors or funders play any role in the study design, data collection and analysis, decision to publish, or preparation of the manuscript?<br><br>Did you receive funding for this work?	
Please add funding details.<br>as follow-up to "**Financial Disclosure**<br><br>Enter a financial disclosure statement that describes the sources of funding for the work included in this submission. Review the submission guidelines for detailed requirements. View published research articles from *PLOS ONE* for specific examples.<br><br>This statement is required for submission and **will appear in the published article** if the submission is accepted. Please make sure it is accurate.	Accelerating Genetic Gain (AGG) in Maize and Wheat Project Grant INV-003439 funded by the Bill and Melinda Gates Foundation (BMGF), the Foreign and Commonwealth Development Office (FCDO)

Powered by Editorial Manager® and ProduXion Manager® from Aries Systems Corporation

**Funded studies**<br>Enter a statement with the following details:<br>• Initials of the authors who received each award<br>• Grant numbers awarded to each author<br>• The full name of each funder<br>• URL of each funder website<br>• Did the sponsors or funders play any role in the study design, data collection and analysis, decision to publish, or preparation of the manuscript?<br><br>Did you receive funding for this work?"	
Please select the country of your main research funder (please select carefully as in some cases this is used in fee calculation).<br>as follow-up to "**Financial Disclosure**<br><br>Enter a financial disclosure statement that describes the sources of funding for the work included in this submission. Review the submission guidelines for detailed requirements. View published research articles from *PLOS ONE* for specific examples.<br><br>This statement is required for submission and **will appear in the published article** if the submission is accepted. Please make sure it is accurate.<br><br>**Funded studies**<br>Enter a statement with the following details:<br>• Initials of the authors who received each award<br>• Grant numbers awarded to each author<br>• The full name of each funder<br>• URL of each funder website<br>• Did the sponsors or funders play any role in the study design, data collection and analysis, decision to publish, or preparation of the manuscript?<br><br>Did you receive funding for this work?"	UNITED STATES - US
**Competing Interests**	No competing interests

Use the instructions below to enter a competing interest statement for this submission. On behalf of all authors, disclose any competing interests that could be perceived to bias this work—acknowledging all financial support and any other relevant financial or non-financial competing interests.<br><br>This statement is required for submission and **will appear in the published article** if the submission is accepted. Please make sure it is accurate and that any funding sources listed in your Funding Information later in the submission form are also declared in your Financial Disclosure statement.<br><br>View published research articles from *PLOS ONE* for specific examples.<br><br>**NO authors have competing interests**<br><br>Enter: *The authors have declared that no competing interests exist.*<br><br>**Authors with competing interests**<br><br>Enter competing interest details beginning with this statement:<br><br>*I have read the journal's policy and the authors of this manuscript have the following competing interests: [insert competing interests here]*<br><br>* typeset	
**Ethics Statement**<br><br>Enter an ethics statement for this submission. This statement is required if the study involved:<br><br>• Human participants<br>• Human specimens or tissue<br>• Vertebrate animals or cephalopods<br>• Vertebrate embryos or tissues	NA

• Field research<br><br>Write "N/A" if the submission does not require an ethics statement.<br><br>General guidance is provided below. Consult the submission guidelines for detailed instructions. **Make sure that all information entered here is included in the Methods section of the manuscript.**	

**Format for specific study types**<br><br>**Human Subject Research (involving human participants and/or tissue)**<br>• Give the name of the institutional review board or ethics committee that approved the study<br>• Include the approval number and/or a statement indicating approval of this research<br>• Indicate the form of consent obtained (written/oral) or the reason that consent was not obtained (e.g. the data were analyzed anonymously)<br><br>**Animal Research (involving vertebrate animals, embryos or tissues)**<br>• Provide the name of the Institutional Animal Care and Use Committee (IACUC) or other relevant ethics board that reviewed the study protocol, and indicate whether they approved this research or granted a formal waiver of ethical approval<br>• Include an approval number if one was obtained<br>• If the study involved *non-human primates*, add *additional details* about animal welfare and steps taken to ameliorate suffering<br>• If anesthesia, euthanasia, or any kind of animal sacrifice is part of the study, include briefly which substances and/or methods were applied<br><br>**Field Research**<br><br>Include the following details if this study involves the collection of plant, animal, or other materials from a natural setting:<br>• Field permit number<br>• Name of the institution or relevant body that granted permission	
**Data Availability**<br><br>Authors are required to make all data underlying the findings described fully available, without restriction, and from the time of publication. PLOS allows rare exceptions to address legal and ethical concerns. See the PLOS Data Policy and FAQ for detailed information.	Yes - all data are fully available without restriction

A Data Availability Statement describing where the data can be found is required at submission. Your answers to this question constitute the Data Availability Statement and **will be published in the article**, if accepted.<br><br>**Important:** Stating 'data available on request from the author' is not sufficient. If your data are only available upon request, select 'No' for the first question and explain your exceptional situation in the text box.<br><br>Do the authors confirm that all data underlying the findings described in their manuscript are fully available without restriction?	
**Describe where the data may be found in full sentences. If you are copying our sample text, replace any instances of XXX with the appropriate details.**<br><br>• If the data are **held or will be held in a public repository**, include URLs, accession numbers or DOIs. If this information will only be available after acceptance, indicate this by ticking the box below. For example: *All XXX files are available from the XXX database (accession number(s) XXX, XXX.)*.<br>• If the data are all contained **within the manuscript and/or Supporting Information files**, enter the following: *All relevant data are within the manuscript and its Supporting Information files.*<br>• If neither of these applies but you are able to provide **details of access elsewhere**, with or without limitations, please do so. For example:<br><br>*Data cannot be shared publicly because of [XXX]. Data are available from the XXX Institutional Data Access / Ethics Committee (contact via XXX) for researchers who meet the criteria for access to confidential data.*<br><br>*The data underlying the results presented in the study are available from (include the name of the third party*	The datasets generated for this study are available on request from the corresponding and first author.

and contact information or URL).<br>• This text is appropriate if the data are owned by a third party and authors do not have permission to share the data.<br><br>* typeset	
Additional data availability information:	Tick here if the URLs/accession numbers/DOIs will be available only after acceptance of the manuscript for publication so that we can ensure their inclusion before publication.

Powered by Editorial Manager® and ProduXion Manager® from Aries Systems Corporation

Manuscript
Click here to access/download;Manuscript;Final BGD432 WB Manuscript_Plos One_PK.docx

Original Research Article

Field screening and genetic mapping of wheat blast resistance for a panel of common wheat from Bangladesh

Md. Farhad[1], Xinyao He[2], Hirokazu Handa[3], Muhammad Rezaul Kabir[1], Krishna Kanta Roy[1], Felix Marza[4], Masahiro Kishi[5], Soichiro Asuke[6], Pawan K. Singh[2]*

[1] Bangladesh Wheat and Maize Research Institute (BWMRI), Nashipur, Dinajpur, Bangladesh

[2] International Maize and Wheat Improvement Center (CIMMYT), Mexico DF, Mexico

[3] Graduate School of Life and Environmental Sciences, Kyoto Prefectural University, Kyoto, Japan

[4] Instituto Nacional de Innovación Agropecuaria y Forestal (INIAF), La Paz, Bolivia

[5] Japan International Research Center for Agricultural Sciences, Tsukuba, Japan

[6] Graduate School of Agricultural Science, Kobe University Kobe, Japan

[*]*Corresponding author's email:* pk.singh@cgiar.org

Abstract

Wheat blast, caused by *Magnaporthe oryzae* pathotype *Triticum* (MoT), is a rapidly emerging disease threatening wheat production in South Asia and beyond. To identify novel sources of resistance and dissect the underlying genetic architecture, a genome-wide association study (GWAS) was conducted using 432 diverse Bangladeshi wheat genotypes phenotyped across 14 multi-environment trials. Disease severity varied widely, with most genotypes exhibiting high susceptibility under favourable environments, although a few showed consistent resistance across locations. Genotyping of the wheat panel using ddRAD-seq revealed moderate population structure and diverse resistance allele distribution. GWAS using six different models identified a total of 1,121 significant SNPs distributed across multiple chromosomes. The 2NS translocation and known resistance genes (*Rmg8*, *Rwt3*, *Rwt4-1B*, *Rwt4-1D*) were unevenly distributed across the panel. Based on linkage disequilibrium (LD), the significant markers were grouped into 45 quantitative trait loci (QTL) associated with wheat blast resistance, including the 2NS/2AS translocation. These QTLs either coincide with known blast resistance loci or represent potentially novel ones. These findings enhance the understanding of wheat blast resistance and provide valuable markers and candidate genotypes for resistance breeding aimed to mitigate the threat of wheat blast under changing climates.

Keywords

Wheat Blast, GWAS, Linkage Disequilibrium, SNP, QTL

Introduction

Wheat blast (WB) is caused by *Magnaporthe oryzae* pathotype *Triticum* (MoT) and shows symptoms on all the aerial parts of the wheat plant; but it is the bleached spikes that serve as the most conspicuous symptom and cause the most severe yield damages up to 100% (1). This is a relatively new disease, which had been confined within South America since its first report in 1985 in Brazil until its outbreak in Bangladesh in 2016 (2). In 2018, WB was reported for the first time in Zambia, announcing its incidence in a new continent, Africa (3). Such inter-continental spread of WB is generally ascribed

to its seed-borne nature and the intense international wheat trading (4,5), highlighting the importance of quarantine measures in preventing the introduction of MoT. Prediction models have indicated that tropical and sub-tropical regions are at moderate to high risk, like parts of India, Ethiopia, China, Pakistan, USA, Australia etc., as recently reviewed by a study (5). It is noteworthy, however, that additional regions might become vulnerable to WB under projected changes in agricultural practices and climate change scenarios.

The disease develops very fast, and it takes only a few days to completely damage the spikes after the appearance of first symptoms; therefore, WB can only be prevented but not cured. So far, no immunity against the disease has been identified in common wheat, nor in its relatives; nevertheless, varietal resistance remains an indispensable component in the WB management systems. Ever since the disease was identified in South America, large-scale germplasm screening activities were conducted to seek for resistant sources; however, resistant genotypes reported in early studies often turned to be susceptible, due to the evolution of MoT isolates that overcame the host resistance (4). At present, the most extensively utilized resistance source is the 2NS chromosomal segment, introgressed from *Aegilops ventricosa* as a AS·2NS translocation through the French line VPM1(6), replacing the distal region of wheat chromosome 2AS. This alien segment confers a strong protective effect, reducing wheat blast severity by 50.4–80.5% across a panel of 418 genotypes (7). However, new isolates that are virulent to 2NS have evolved and are spreading in South America (4,8), severely undermining the resistance conferred by 2NS. It is therefore imperative to identify new resistant sources to alleviate the strong selection pressure brought by the 2NS translocation.

Resistance against WB could be both qualitative and quantitative, but the former has only been observed at the seedling stage. A few major resistance genes have been identified at the seedling stage, i.e., *Rmg2*, *Rmg3*, *Rmg7*, *Rmg8*, *RmgGR119*, *Rmg10*, and *Rmg11*, of which the first three have been overcome by new MoT isolates, *Rmg8* and *RmgGR119* demonstrated effective resistance in greenhouse but need to be validated in large scale field trials (9), and the last two exhibited better resistance at the seedling stage than at the heading stage (10). Great efforts have been recently conducted to identify QTL associated with field WB resistance, using both bi-parental populations and genome-wide

association study (GWAS) panels. In most of such studies, 2NS was the only consistently significant locus with major effects, whereas the non-2NS loci were of minor effects and inconsistent (11–20). In contrast, a previous study (21) analysed two bi-parental populations derived from non-2NS sources (Anahuac 75 and BRS 179) and detected five QTL at the seedling stage and four QTL at the adult-plant stage. However, the experiments were conducted under controlled conditions, thus the QTL need to be further validated in field experiments. Recently, a major QTL for WB resistance, designated *Qwb.cim-7D*, was mapped to the long arm of chromosome 7D. This QTL, derived from an *Ae. tauschii* line KU-2097, explained 7.7–50.6% of the phenotypic variation in different field experiments.(22).

Bangladesh is a developing country with a rapid increase in wheat consumption in the last decade; but its wheat production lagged far behind, as reflected in yearly consumption of about 7 million metric tons (MMT) vs. yearly production of around 1.2 MMT (23). The 2016 WB outbreak (24) brought big yield reduction in the affected regions, and the government had to suspend wheat cultivation in those regions and promote alternative crops like boro rice, maize, gram etc., that are not affected by WB (5). The release of WB resistant cultivars BARI Gom 33, BWMRI Gom 3 and BWMRI Gom 5, which carry the 2NS/2AS translocation, combined with government support for increased wheat production, led to the resumption of wheat cultivation in the WB epidemic regions (5,25). Nevertheless, wheat production in Bangladesh and its neighbouring countries is still under serious threat of WB, considering the fast-evolving MoT pathogen that is eroding the 2NS resistance in South America (4,8). Effective fungicides have been identified for field application against WB in the country (26), though, they are often not satisfactory and may lead to fungal resistance when used in high dosages or frequencies (27). Timely sowing is critical to avoid the high WB disease pressure that often happens in late wheat season (11). Nevertheless, varietal resistance remains the most farmer-friendly management method, which does not require additional financial and technical inputs, and is compatible with other disease management strategies.

The aims of the current study were to 1) screen a panel of wheat genotypes from Bangladesh for WB resistance in field experiments, and 2) identify molecular markers and chromosome regions or QTL associated with WB resistance using GWAS and LD decay analysis.

Material and methods

Plant materials

A diverse panel of 432 common wheat (*Triticum aestivum* L.) genotypes with spring growth habit was used in this study. The panel included a representative collection of germplasm cultivated or evaluated in Bangladesh, including a wide range of agro-morphologically and genetically diverse accessions. These are made up of officially released varieties, advanced breeding lines under national and international trials, and their respective parental lines. A significant portion of the panel included breeding lines developed by the International Maize and Wheat Improvement Centre (CIMMYT), which have been widely utilized in South Asia for their high yield potential and resistance to biotic and abiotic stresses. The panel also incorporated elite cultivars and breeding lines sourced from India and China, selected based on their regional importance, agronomic adaptation, and relevance to wheat blast resistance breeding efforts.

Field experiments

The field experiments were conducted across three locations: Quirusillas (Quir), and Okinawa (Okin) in Bolivia, and Jashore (Jash) in Bangladesh. The cropping season spans from December to April in both Quirusillas and Jashore, and from May to September in Okinawa. Field trials were conducted during the 2019–20 and 2020–21 cropping seasons in Quirusillas, the 2020 and 2021 seasons in Okinawa, and the 2018–19, 2019–20, and 2020–21 seasons in Jashore. Each trial included two sowing dates approximately 10 days apart, resulting in a total of 14 experiments. The experiments are designated using a naming style that includes the location, year of disease evaluation, and sowing time (with ‘a’ for the first sowing and ‘b’ for the second). For example, Okin21b refers to the second sowing in the 2021 trial conducted at Okinawa.

The plants were sown in 1m double rows separated by 20 cm spacing in all three locations, and no replication was arranged within each sowing. To ensure a favourable micro-environment for wheat blast development, mist-irrigation systems were installed at each site and operated daily from 8:00 AM to 7:00 PM, providing 10 minutes of misting per hour during the disease development period. Field

inoculations were conducted twice in each experiment using a backpack sprayer—once at anthesis and again two days later. A mixture of locally collected MoT isolates with high virulence was used as inoculum, involving isolates QUI1505, QUI1601, QUI1612, OKI1503 and OKI1704 in Quirusillas and Okinawa, and BHO17001, MEH17003, GOP17001.2, RAJ17001, CHU16001.3 and JES16001 in Jashore. Spore production was conducted on oatmeal agar medium following the protocol by (11), and the inoculum solution was adjusted to a concentration of 80,000 spores/mL for field application. Local checks used in the experiments were Urubo (resistant check) and Atlax (susceptible check) in Bolivia and BARI Gom 33 (resistant check) and BARI Gom 26 (susceptible check) in Bangladesh.

For WB evaluation, 10 spikes per plot were marked with coloured scotch tape at anthesis and evaluated at 21 days after anthesis, for which the total and infected number of spikelets were counted to derive incidence (the proportion of spikes with blast infection) and severity (the proportion of spikelet's damaged in infected spike). WB index was calculated with the formula WB index = incidence × severity and was used in all subsequent analysis. Field phenotyping data were curated to keep robust data set for the study. The phenotypic data of the WB index (%), capturing the spectrum of wheat blast responses under diverse conditions, were then used as input for subsequent genome-wide association studies (GWAS) to identify genetic loci associated with resistance and downstream QTL mapping. Within-environment analysis of variance (ANOVA) was performed using the Metan v1.19.0 package in R. In each location-season combination, different planting times were considered as replications to account for temporal variation in disease pressure and environmental conditions.

Genotyping

All the 432 accessions in this study were genotyped using the double digest restriction-site associated DNA sequencing (ddRAD-seq) method. Genomic DNA was digested with *Bgl*II and *Eco*RI, ligated with Y-shaped adaptors, amplified by PCR with KAPA HiFi HS ReadyMix (Kapabio Systems, UK), and then size-selected with the E-Gel size select (Life technologies, CA, USA). Approximately 450 bp library fragments were retrieved. Further details of the library preparation method were described in a previous study (28). Sequencing was performed with paired-end 151bp mode of HiSeqX (Illumina, CA, USA). After quality filtering and cleaning of sequence reads performed by Cutadapt ver.

3.1 (29), we mapped them to the wheat reference genome sequence, IWGSC RefSeq v2.1 (30) by BWA ver. 0.7.17 (31). Sequence variants were called by Haplotype Caller in GATK ver. 4.1.9.0 (32). We picked up only SNPs and then filtered those for missing data less than 25%, which resulted in 82,948 SNPs. Furthermore, we used BEAGLE v3.3.2 (33) to impute the data based on the available allele frequencies obtained after specifying the haplotype phase for all individuals. After removal of duplicate genotypes, we picked up the markers for minor allele frequency greater than 5%, resulting in 7,211 markers for 432 accessions.

To identify the 2NS/2AS translocation, four sequence-tagged site (STS) markers previously reported for this region were employed: Ventriup-LN2 (34), WGGB156 and WGGB159 (35), and cslVrgal3 (36). For the presence of the *Rmg8* gene (37), we used the functional marker for *Rmg8*, KM171 (38). Genotyping for *Rwt3*, *Rwt4-1B* and *Rwt4-1D* followed the previously used protocols (39).

Population structure and phylogenetic tree annotation

The population structure of the wheat genotypes was assessed using SNPs derived from filtered ddRAD-seq data. A highly annotated phylogenetic tree was generated in TASSEL 5.2 (40) with visualization in Archaeopteryx, and a Neighbor-Joining tree was constructed to examine genetic diversity and potential for GWAS marker discovery. To elucidate the distribution patterns of known wheat blast resistance loci, the NJ tree was annotated with five concentric tracks, each representing genotypic calls for a specific resistance source: the 2NS translocation, *Rwt3* (*Rmg6*), *Rwt4-B1* (*Rmg1*), *Rwt4-D1* (*Rmg1*), and *KM200* (*Rmg8*) using online tool Evolview Version-3 (41,42). Genotypic calls were categorized as homozygous positive (1, blue), heterozygous (0.5, green), homozygous negative (–1, red), or missing/no call (0, light green).

Genome-wide marker-trait associations

GWAS was performed by ‘R’ package GAPIT 3.4.0 (43–45) using six different models viz GLM -General Linear Model (46), MLM -Mixed Linear Model (47,48), MLMM -Multiple Loci Mixed Model (49), SUPER -Settlement of MLM Under Progressively Exclusive Relationship (50), FarmCPU -Fixed and Random Model Circulating Probability Unification (51) and Blink -Bayesian Information and Linkage-Disequilibrium Iteratively Nested Keyway (52). Statistical explanation of each Model is

explained in S1 Doc. Manhattan plots with the $-\log_{10}$ *p*-values of the markers were generated using the 'R' package CMplot v4.5.1 (53). The standard Bonferroni corrections at α levels of 0.05 were used as the significance of the markers for all models in the study. The common MTA in at least two packages or two environments were picked up for QTL analysis.

QTL construction based on LD decay

To delineate QTLs associated with wheat blast resistance, linkage disequilibrium (LD) analysis was performed across all 21 wheat chromosomes using TASSEL v5.2 (40). After genome-wide association analysis (GWAS), SNP markers significantly associated with wheat blast resistance were identified by applying a stringent Bonferroni correction to control for multiple testing, ensuring robust marker-trait associations. To construct QTL intervals from these significant SNPs, we investigated the local LD landscape surrounding each associated marker. Pairwise LD was estimated between the significant SNPs and their flanking markers using two metrics: r^2, which measures allelic correlation, and D′, a normalized measure of recombination between loci. LD heatmaps were generated for each chromosome to visualize the extent and structure of LD blocks by using TASSEL v5.2.

QTL regions were defined by identifying contiguous blocks of high LD surrounding each lead SNP, where LD thresholds of $r^2 \geq 0.70$ and/or $D' \geq 0.85$ were used to delineate QTL boundaries. These regions were interpreted as representing genomic segments harbouring either causal variants or closely linked loci contributing to wheat blast resistance. To visualize the chromosomal distribution of QTLs, a phenogram was constructed using the Phenogram Web Tool developed by the University of Pennsylvania, which enables integration of QTL positions onto wheat genome ideograms(54). Each QTL was assigned a unique identifier (e.g., *Qwb.cim-X.Y*, where *Qwb.cim* = QTL for wheat blast identified by CIMMYT, X denotes the chromosome and Y the serial order of QTLs identified on that chromosome).

For comparative mapping, the physical positions of known wheat blast resistance genes, i.e., *Rmg1*, *Rmg6*, *Rmg8* and the 2NS/2AS translocation segment, were retrieved from published literature and placed onto the same map. The physical distance between QTLs and these known genes was calculated in Megabases (Mb) using their reference genome positions of IWGSC RefSeq v2.1, (30,55)

allowing evaluation of potential co-localization, tight linkage, or independence. This integrative LD-based QTL mapping approach facilitates the identification of both overlapping and novel resistance loci and provides a genomic framework for downstream applications in fine-mapping, marker-assisted selection (MAS), and functional validation for wheat blast resistance.

RESULTS

Phenotypic evaluation of wheat blast resistance across multi-environment trials

The wheat genotypes were phenotypically evaluated for wheat blast resistance under artificial inoculation across 14 distinct environments, representing seven growing seasons in three locations in Bangladesh and Bolivia. The maximum severity scored for each genotype across all environments was used to assess its overall resistance performance. Substantial phenotypic variation was observed among the genotypes, reflecting a broad spectrum of resistance responses (Fig 1). Only 3% of the entries exhibited complete resistance across all environments, while an additional 8% showed resistant

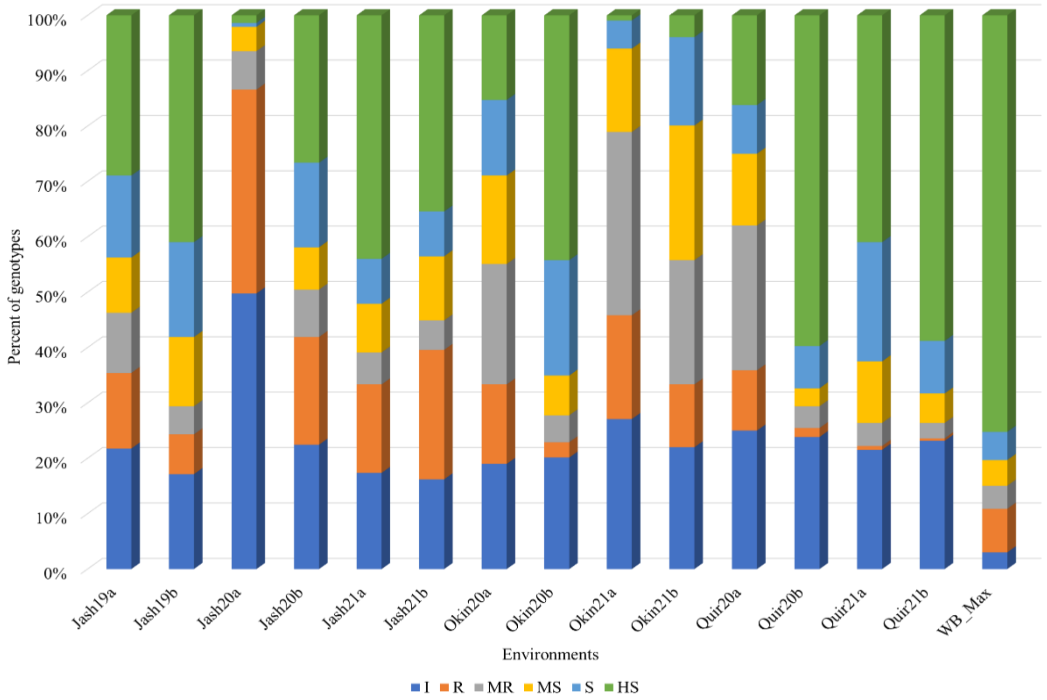

Fig 1: Distribution of wheat blast (WB) severity based on WB index for 432 diverse wheat genotypes evaluated under artificial inoculation across 14 environments in Bangladesh and Bolivia. Reaction categories: I = immune (no symptoms), R = resistant (WB index 1–10%), MR = moderately resistant (11–25%), MS = moderately susceptible (26–40%), S = susceptible (41–60%), and HS = highly susceptible (61–100%)

reactions and 4% were classified as moderately resistant (S1 Table). In contrast, 5% displayed moderately susceptible to susceptible responses, and a striking 75% of the genotypes recorded highly severe infection (WB Index >60%) in at least one environment.

Environmental effects significantly influenced disease expression. Certain environments like Jash20a, Okin21a, and Okin21b exhibited relatively lower disease pressure, with most genotypes showing mild to moderate infection levels. In contrast, severe infection was observed in Jash19a, Jash20a, Jash21b (Bangladesh) and Okin20b, Quir20b, Quir21a, and Quir21b (Bolivia), suggesting that these environments were highly conducive to disease development. The contrast results among environments, including differences between first and second planting windows, suggest potential interactions between sowing time and environmental conditions favouring MoT infection. Within-environment analysis of variance revealed significant effects of both genotype and planting time on wheat blast (WB) index across all environments (Table 1). Notably, the second planting times in Bangladesh and, in a few cases, the first planting times in Bolivia were generally associated with higher WB indices, suggesting increased disease pressure under conditions favourable for blast development. These results highlight the influence of both genetic and temporal factors in shaping wheat blast response under artificial inoculation across diverse environments.

Table 1: Within-environment analysis of variance for WB Index

Environments	Average WB Index	Df (Genotype)	Df (Sowing Time)	Error Df	F-value (genotype)	F-value (sowing time)
Jashore 19	44.9	449	1	449	3.23***	62.2***
Jashore 20	22	449	1	449	1.68***	396***
Jashore 21	46.7	447	1	442	7.92***	41.7***
Okinawa 20	38	449	1	448	3.21***	229***
Okinawa 21	19.4	448	1	441	1.83***	70.5***
Quirusillas 20	43	449	1	449	2.93***	368***
Quirusillas 21	54.2	447	1	446	8.49***	87.7***

The visualization of field data in Fig 2 and S1 Fig further supports these findings. The boxplot revealed a median position within the fourth quartile, indicating that the majority of genotypes experienced high WB index (%) in at least one environment. The histogram, density, and cumulative

distribution plots illustrated a skewed distribution toward high blast scores, reinforcing the dominance of susceptible responses. These trends collectively point to strong genotype-by-environment (G×E) interactions, wherein resistance expression varied considerably across testing conditions.

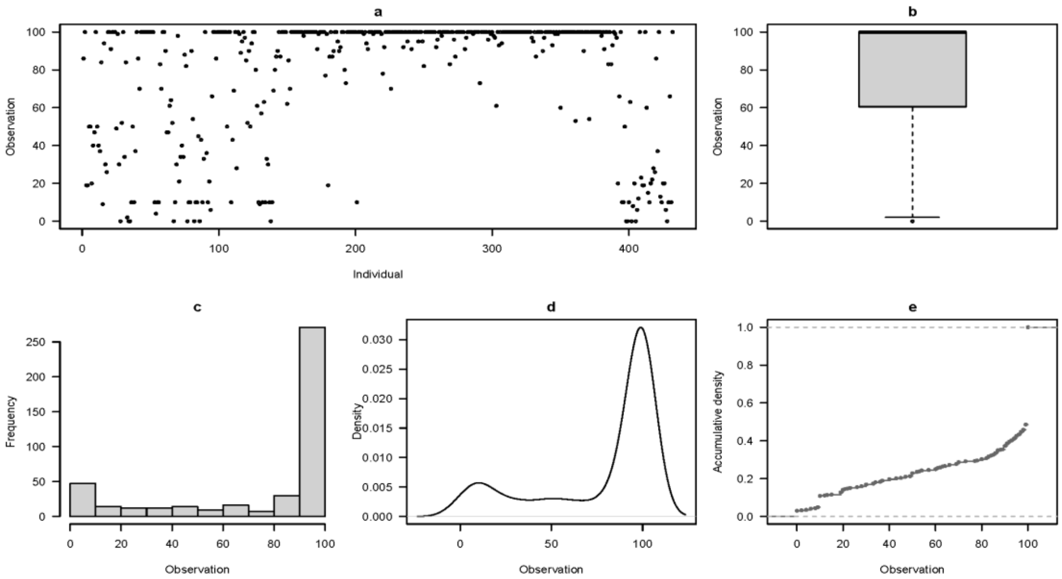

Fig 2. Field performance of the diverse wheat genotypes for wheat blast resistance across experiments. The figure summarizes maximum wheat blast scores pooled from all environments and includes the following visualizations: (a) scatter plot showing individual genotype scores; (b) box plot indicating that most genotypes fall within the upper quartile, suggesting high susceptibility in at least one environment; (c) histogram displaying the distribution of scores; (d) density plot illustrating the distribution pattern; and (e) cumulative density plot highlighting the prevalence of higher wheat blast scores. The wide range of values reflects substantial variability in resistance among the tested genotypes (S1 Fig illustrated for additional details of 14 environments, representing seven growing seasons in three locations).

Despite the overall prevalence of susceptibility, a small subset of genotypes demonstrated consistent resistance across diverse environments, indicating their potential as sources of stable, broad-spectrum resistance. These genotypes warrant further genetic characterization and validation under both natural and artificial epidemic conditions.

Genome-Wide SNP distribution and population structure analysis

The high-quality SNPs were distributed across the 21 chromosomes of the wheat genome, with the B genome exhibiting the highest marker saturation, followed by the A genome. The D genome displayed comparatively lower marker density, which is consistent with its narrower genetic diversity and lower polymorphism rate in hexaploid wheat. The highest number of markers were present in the B-genome (47.48%) followed by A-genome (40.76%) and the D-genome (10.89%). The heatmap

visualization illustrates SNP density across genomic intervals, indicating adequate marker coverage for robust genome-wide association analysis (Fig 3a).

Principal Component Analysis (PCA) was conducted to explore the underlying genetic structure of the 432 genotypes. The scree PCA plot (Fig 3b) showed that the first three principal components explained 6.39%, 5.01%, and 4.22% of the total variance, respectively. The eigenvalue pattern indicates that the majority of genetic variation is captured within the top components, supporting their use in population structure correction in GWAS models. The 3D PCA plot (Fig 3c) revealed a moderately structured population, with genotypes showing continuous but distinguishable clustering. This reflects the presence of subpopulation structure and possible ancestral divergence among the genotypes. A good example is the small cluster clearly separated from the main group, being composed of synthetic wheat derivatives (Fig 3c). Such stratification necessitates correction during association analysis to control for spurious associations.

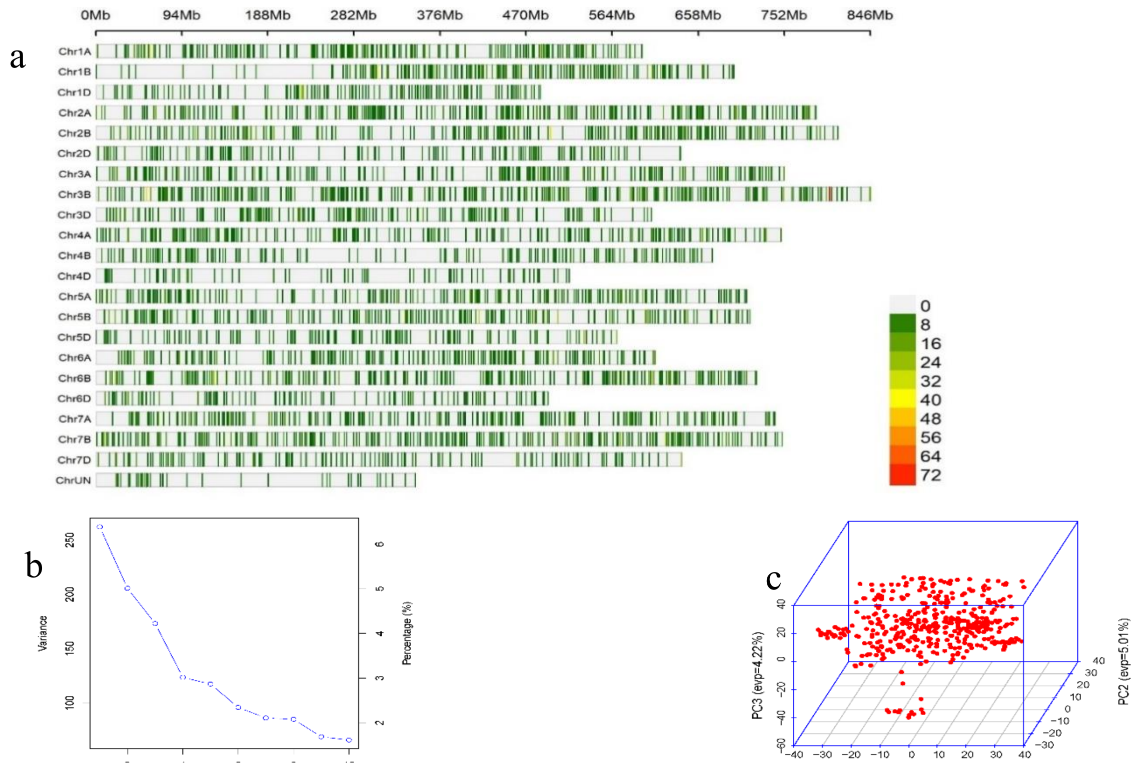

Fig 3: Genomic characteristics and principal component analysis (PCA) based on ddRAD-seq-derived SNP markers across 432 wheat genotypes. (a) Genome-wide marker density plot showing the distribution of SNPs across the wheat chromosomes; (b) Scree plot of eigenvalues indicating the proportion of total genetic variance explained by each principal component, used to determine the number of informative components; and (c) 3D PCA plot illustrating the genetic diversity and clustering patterns among the wheat genotypes based on the top three principal components.

Genotyping of the panel with markers for cloned *Rmg* genes

Genotyping results with the diagnostic markers indicated high frequencies of the non-MoT resistance genes, i.e., 64% of the genotypes have *Rmg6* (*Rwt3*), 52% have *Rwt4-1B* (allelic to *Rmg1*), and 95% have *Rmg1* (*Rwt4-1D*). In total, 29% of the genotypes have all three genes, whereas one genotype had none. As for the MoT resistance gene *Rmg8*, only one genotype was diagnosed to be positive (Fig 4). As expected, these non-MoT resistance genes showed no association with WB resistance. This was evident in genotypes carrying all three non-2NS resistance genes, which still exhibited moderate to high disease severity when the 2NS translocation was absent (Table S1).

Annotation of 2NS and *Rmg* loci into phylogenetic structure

The Neighbor-Joining (NJ) tree constructed from genome-wide SNP data revealed a well-defined population structure among the evaluated wheat genotypes (Fig. 4). Integration of molecular marker data with the NJ tree showed non-random distribution patterns of resistance loci. The 2NS translocation was predominantly associated with a distinct and cohesive cluster of genotypes, suggesting a shared genetic background or selective retention of this segment. Similarly, *Rwt3 (Rmg6)* and both homeologous copies of *Rwt4—Rwt4-1B* and *Rwt4-1D* (*Rmg1*)—were enriched within specific sub-clades, indicating a structured deployment of these loci across the germplasm. A total of 182 genotypes carried both *Rwt4-1B* and *Rwt4-1D*. Notably, their co-occurrence across several phylogenetic branches supports the hypothesis that these loci have been co-maintained, potentially due to their location on homeologous chromosomes and/or co-selection during breeding, consistent with expected homeologous chromosome patterns in wheat. In contrast, the *Rmg8* gene was detected in only a single genotype, indicating its extremely low frequency within the current panel. Overall, the annotated NJ tree highlights a non-random, structured distribution of wheat blast resistance genes, reflecting clear genetic stratification among the genotypes.

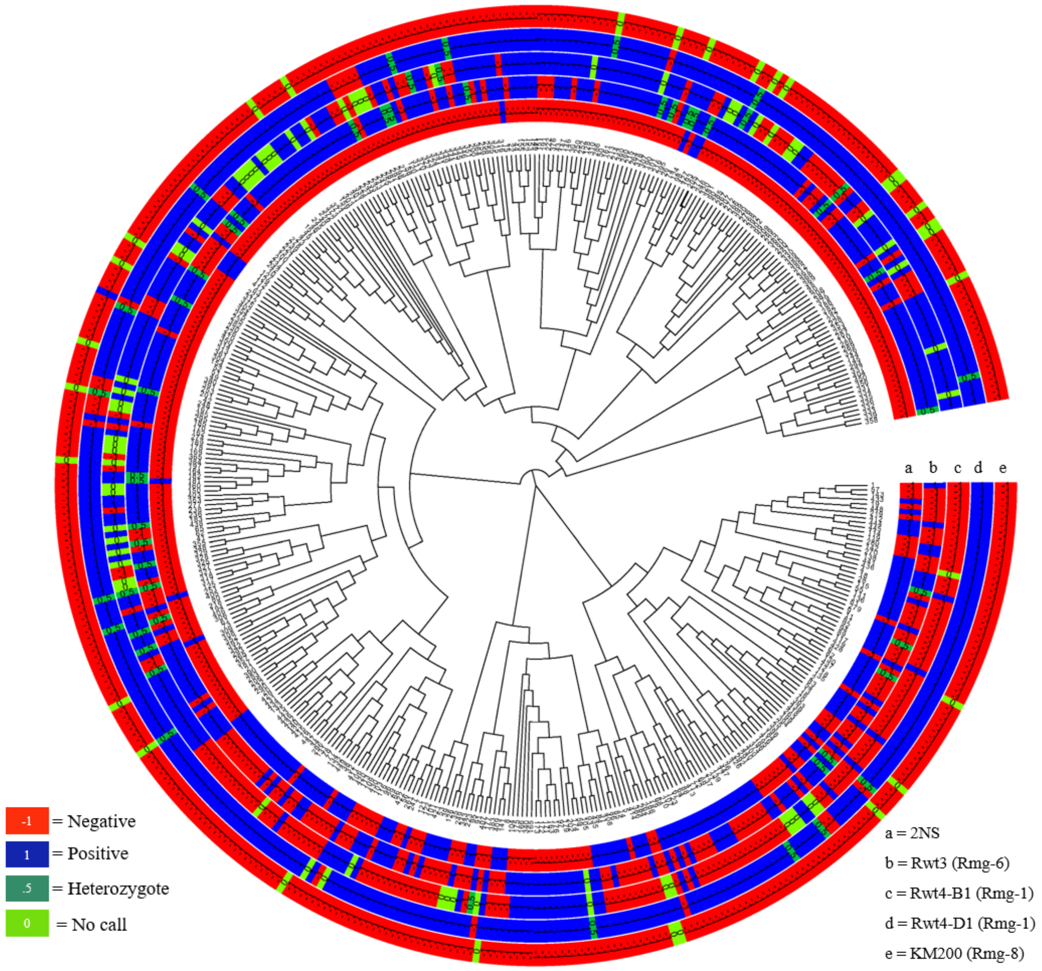

Fig 4: Neighbour-Joining tree based on genetic distances among the 432 bread wheat genotypes, showing the distribution of four *Rwt* (*Rmg*) genes and the 2NS translocation.

Linkage Disequilibrium (LD) decay analysis

Genome-wide LD analysis revealed genome-specific patterns across the 21 wheat chromosomes (S3 Fig). Consistent with previous findings, chromosomes of the D genome exhibited larger and more continuous LD blocks, reflecting lower historical recombination rates and reduced marker coverage. In contrast, the A and B genomes displayed a more fragmented LD landscape, indicative of more frequent historical recombination events and faster LD decay. These LD patterns align with the known evolutionary dynamics of hexaploid wheat and provide a genomic context for interpreting association signals.

Genome-wide association study for wheat blast resistance

Six different GWAS models—MLM, MLMM, SUPER, BLINK, FarmCPU, and GLM—were employed using GAPIT package in R to ensure robust and comprehensive detection of associated markers. Across the models, a total of 1,121 significant SNPs were identified after applying Bonferroni correction (alpha < -0.05), distributed across multiple chromosomes. These associations suggest the presence of diverse resistance loci effective under varying environmental conditions. Notably, strong associations were observed on chromosomes 2A, 3A, 3B, 5A, and 6B, highlighting these regions as potential hotspots for WB resistance. Within the D genome, chromosome 4D showed the highest number of MTAs, with 76 out of 80 significant SNPs in the genome, indicating that WB resistance may have been introgressed through natural wide crosses involving 4D, and that this chromosome could be a key target for future breeding.

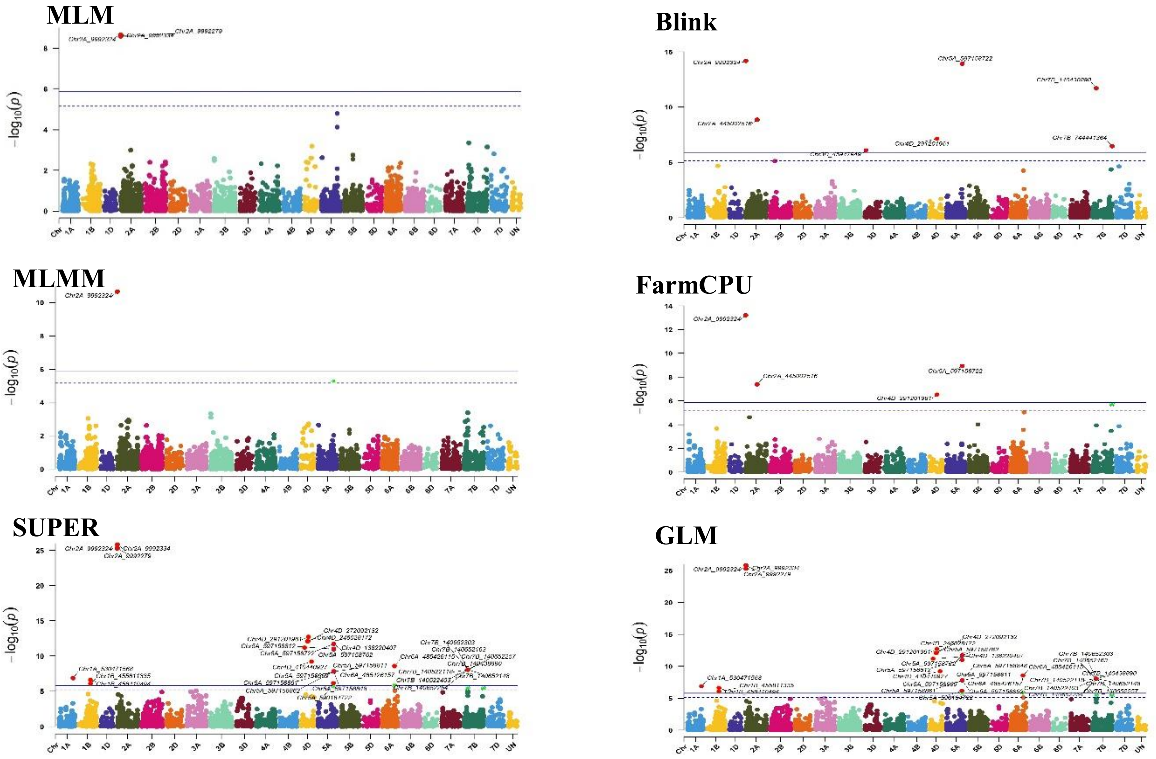

Fig 5: Manhattan Plots Showing GWAS Results Using Six Different Models. The plots were drawn based on the pooled highest wheat blast (WB) scores across 14 environments. Individual Manhattan plots for each environment are presented in S2 Fig.

Among the tested models, MLM, MLMM, BLINK, and FarmCPU demonstrated higher detection power and resolution, consistent with their previously reported effectiveness in analysing complex traits with moderate to high heritability. The Manhattan plots in Fig 5 represent associations

based on the highest WB index values observed for each genotype across all environments. Additional Manhattan plots for individual environments are presented in Fig. S2. These SNP markers identified form GWAS was then used for QTL identification.

QTL identification

A total of 45 QTL were identified across the wheat genome, each supported by statistically robust associations derived from Bonferroni-adjusted thresholds and validated through significant

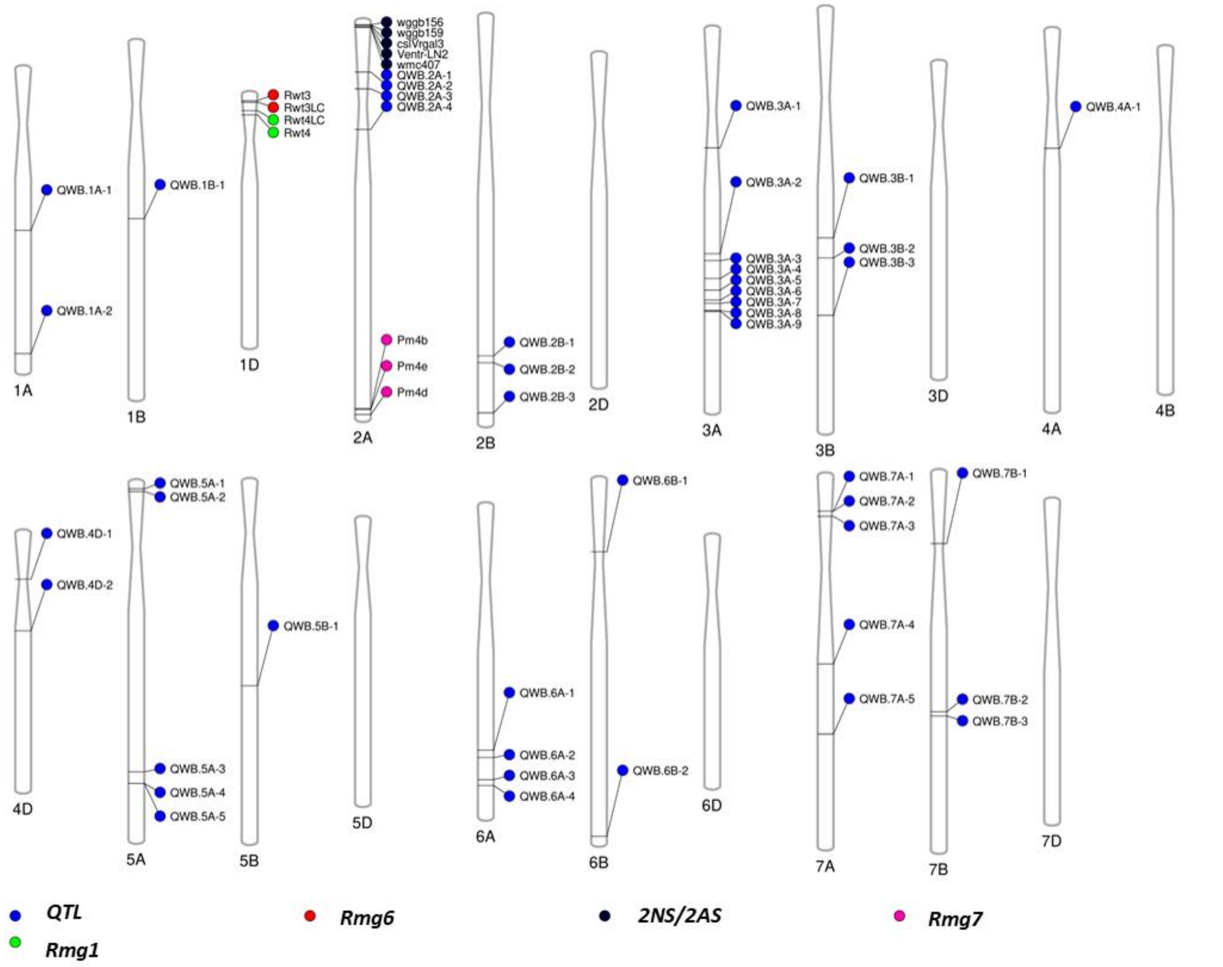

Fig 6: Genome wide QTL distribution for wheat blast resistance identified from the 14 different field experiments using six different models. Integrated wheat blast resistance QTL map constructed using a phenogram-based visualization tool. A total of 45 QTLs associated with wheat blast resistance are displayed in blue dots across the wheat genome. Regions enriched with QTLs are observed particularly on chromosomes 2A, 3A, 3B, 5A, 5B, 6A, 6B, 7A, and 7B. The map also shows the position of several *Rmg* (*Rwt*) genes and the 2NS/2AS translocation.

linkage disequilibrium (LD) structures, reflecting both the strength and reliability of these signals (S2 Table). To enhance the understanding of the genetic architecture underpinning wheat blast resistance, a comprehensive QTL map was developed using a phenogram-based visualization approach (54). This

integrated map delineates both genotyped *Rmg* (*Rwt*) genes and QTLs identified in the current study. The QTLs were predominantly distributed across chromosomes 2A, 3A, 3B, 5A, 5B, 6A, 6B, 7A, and 7B (Fig 6). Among these, chromosome 2A emerged as particularly significant due to the presence of four QTLs (*Qwb.cim-2A.01* to *Qwb.cim-2A.04*), with the first corresponds to the well-characterized 2NS/2AS translocation segment. The last three QTL on this chromosome exhibited long distance up to 88 Mb from the 2NS translocation, suggesting independent resistance factors. No QTL was detected in the *Rmg7* region on chromosome 2AL, which is closely linked to the powdery mildew resistance genes *Pm4b*, *Pm4e*, and *Pm4d* (38). The absence of any significant QTLs on chromosome 1D confirmed that *Rmg1* and *Rmg6*, being non-MoT resistance genes located on 1D, did not contribute to WB resistance.

The remaining 41 *QWB* loci, which are located in genomic regions without known *Rmg* genes, may represent new or previously uncharacterized candidate regions for blast resistance. While these regions do not overlap with reported *Rmg* loci, further comparison with previously mapped QTL is needed to determine whether they coincide with earlier resistance signals or are truly novel. These loci are distributed across 3A, 3B, 5A, 5B, 6A, 6B, 7A, and 7B, with particularly high QTL density on 3A, 5A, and 7A.

Discussion

Phenotypic evaluation of wheat blast resistance in this panel of wheat genotypes across multi-environment trials revealed that a small proportion (15%) of genotypes were consistently resistant (I to MR reactions), whereas the majority (75%) exhibited a wheat blast index greater than 50% in at least one environment. This skewed distribution highlights the scarcity of stable resistance in current germplasm and underscores the persistent threat of MoT under conducive conditions (1,24,56). Only a small fraction of genotypes (3%) showed consistent resistance across all test environments, marking them as potential sources of durable resistance.

Strong genotype-by-environment (G×E) interactions were evident, with considerable variation in disease expression across sites and seasons. Environments such as Jash19a, Jash20a, and Quir21a

were favourable for disease development, while others, like Okin21a and Jash20a, showed lower disease pressure. These findings support previous reports indicating that wheat blast severity is highly influenced by local microclimatic factors, particularly temperature and humidity during heading and grain filling stages (1,7,57,58). The observed difference in disease incidence between early and late sowing windows may also reflect environmental shifts impacting pathogen virulence and host susceptibility.

The ddRAD-seq genotyping provided high-density SNP coverage across the wheat genome, with a higher marker density in the B and A genomes than in the D genome, aligning with established patterns of genomic diversity in hexaploid wheat (59). Principal Component Analysis (PCA) revealed a moderately structured population, which necessitated the inclusion of population structure covariates in GWAS to mitigate spurious associations (60). The NJ tree further illustrated this structure, revealing distinct clustering patterns corresponding to the presence of known resistance loci. The integration of gene-specific markers revealed that the 2NS/2AS translocation and cloned resistance genes (*Rwt3*, *Rwt4-B1*, and *Rwt4-D1*), were unevenly distributed. Notably, 36% of genotypes lacked *Rwt3*, and nearly half did not carry *Rwt4-B1*, despite their documented contribution to non-host resistance against multiple *Magnaporthe* pathotypes (61).

The identification of 45 QTLs across multiple chromosomes through GWAS and LD mapping significantly expands the known genetic architecture of wheat blast resistance. The concentration of four QTLs on chromosome 2A reinforces its pivotal role in resistance expression (5,19,56,62). No QTL was identified on chromosome 1D near the known resistance genes *Rmg1* and *Rmg6*, because they are non-MoT resistance genes and are not expected to show significant effects under field experiments inoculated with MoT isolates. On the contrary, *Rmg8* on chromosome 2B is a well-known MoT resistance gene that has shown good resistance effects in greenhouse experiments (63). However, its effects under field conditions need to be further validated. No QTL was identified on the chromosomal region harbouring this gene, which was due to the extremely low frequency in this panel (only one genotype carried this gene) that made it impossible to evaluate its effects in the experiments. More specifically designed experiments are thus needed to evaluate the effects of *Rmg8* on WB resistance in

field experiments. The clustering of QTLs in the genome may indicate genomic hotspots for resistance, potentially reflecting loci with pleiotropic effects, multiple tightly linked resistance genes, or regions involved in quantitative disease response mechanisms such as reactive oxygen species (ROS) regulation, pathogen-associated molecular pattern (PAMP) signalling, or systemic acquired resistance (SAR).

It is noteworthy that there are many lines lacking one or two of the tested non-host resistance genes, and there is one line lacking all three genes. Lacking *Rwt3* means those wheat genotypes are naturally susceptible to the *Magnaporthe oryzae* pathotype *Lolium* (MoL), in addition to their susceptibility to *Triticum* pathotype (MoT), imposing additional risks to farmers growing such varieties (61). Lacking both *Rwt3* and *Rwt4* will make the wheat genotypes susceptible to *Avena* pathotype (MoA), in addition to MoT and MoL, and becoming more vulnerable to *Eleusine* pathotype (MoE), a pathotype known to be prevalent in India that causes the blast disease in finger millet(2). Another implication of the results is that the genotypes without *Rwt4-D1* (*Rmg1*) may have difficulties in utilizing *Rmg8* for improving WB resistance, because the former is known to be a prerequisite for the latter to be functional under the scenario that MoT acquires the virulence factor *PWT4* (64). Therefore, it is beneficial for breeders to introduce both *Rmg8* and *Rmg1* (if not already present in the recipient genotype) to prevent the situation of resistance breakdown of *Rmg8* in future. Collectively, this high-resolution QTL map offers a robust framework for marker-assisted selection (MAS) in wheat blast resistance breeding. It not only validates known sources of resistance but also provides novel targets for fine mapping, gene cloning, and functional characterization, which are crucial for designing durable, broad-spectrum resistance in elite wheat cultivars. The integration of these *Qwb.cim* loci into breeding pipelines holds promise for enhancing resilience against MoT under diverse agro-ecological settings.

Together, these findings offer a comprehensive framework for deploying diverse resistance loci in breeding programs. The integration of phenotypic data, population genomics, LD landscape, and QTL information presents opportunities to design targeted breeding strategies for durable resistance. Future efforts should prioritize the pyramiding of complementary resistance loci, particularly those

exhibiting stable performance across environments, to enhance resilience against wheat blast under variable climatic conditions.

Concluding remarks and recommendations

The present study provides a comprehensive genome-wide insight into the QTL associated with wheat blast resistance in a diverse panel of Bangladeshi wheat genotypes. A total of 45 *Qwb.cim* loci were identified across multiple chromosomes, with several novel loci detected outside the well-characterized 2NS translocation region. These findings reflect the complex and polygenic nature of resistance to wheat blast in Bangladeshi germplasm and underscore the presence of alternative resistance sources beyond the widely utilized 2NS locus. The identification of QTLs on chromosomes 1B, 2B, 4A, 5A, 6A, and 7A—particularly those consistently associated with resistance across multiple environments—provides valuable targets for marker-assisted selection (MAS). These loci represent a foundation for future efforts in fine mapping, functional validation, and candidate gene discovery. Integrating these findings into breeding pipelines will be critical to reduce reliance on 2NS-based resistance, which may be prone to pathogen evolution and potential breakdown over time.

Future work should prioritize validation of these loci using biparental populations and near-isogenic lines to ensure their stability and effectiveness. Fine mapping of major QTL regions is recommended to identify tightly linked markers and candidate genes for cloning. Pyramiding of non-2NS QTLs with existing resistance genes, including the 2NS segment, could enhance the durability and spectrum of resistance in future cultivars. Additionally, the integration of genomic selection approaches using the identified loci could significantly accelerate breeding for wheat blast resistance.

Given the transboundary threat of MoT, collaborative regional efforts involving phenotyping in diverse hot spot environments, sharing of germplasm, and joint validation of resistance loci are essential. The outcomes of this study provide a foundation for developing improved wheat varieties with durable resistance to wheat blast, ensuring yield stability and food security in Bangladesh and other wheat-growing regions of South Asia.

Author contributions

Md. Farhad: Data curation; investigation; methodology; visualization; writing original draft; writing review and editing.

Xinyao He: Investigation; methodology; resources; writing original draft, review and editing.

Hirokazu Handa: Data curation; investigation; methodology; visualization; writing original draft; writing review and editing.

Md. Rezaul Kabir: Conceptualization; investigation; methodology; data collection, project administration; resources; supervision; writing review and editing

Krishna K. Roy: Investigation; methodology; data collection; resources; supervision; writing review and editing

Felix Marza: Investigation; methodology; data collection; resources; supervision; writing review and editing

Masahiro Kishi: Investigation; methodology; data collection; resources; supervision; writing review and editing

Soichiro Asuke: Conceptualization; Investigation; methodology; resources; supervision; writing review and editing

Pawan K. Singh: Conceptualization; funding acquisition; investigation; methodology; project administration; resources; supervision; validation; writing review and editing.

Acknowledgments

Financial supports from the Australian Centre for International Agricultural Research, Australia, One CGIAR Initiatives ABI and PHI, the Ministry of Agriculture, Forestry and Fisheries of Japan, The Krishi Gobeshona Foundation-Bangladesh, and Accelerating Genetic Gain (AGG) in Maize and Wheat Project Grant INV-003439 funded by the Bill and Melinda Gates Foundation (BMGF), the Foreign and Commonwealth Development Office (FCDO) are gratefully acknowledged.

Conflict of interest statement

The authors declare no conflicts of interest.

Data availability statement

The datasets generated for this study are available on request from the corresponding and first author.

References:

1. Cruz CD, Valent B. Wheat blast disease: danger on the move. Trop Plant Pathol. 2017;42:210–22.
2. Valent B, Singh PK, He X, Farman M, Tosa Y, Braun HJ. CHAPTER 13: Blast diseases: Evolution and challenges of a staple food crop fungal pathogen. Emerg Plant Dis Glob Food Secur [Internet]. 2020 Jan [cited 2025 May 1];267–92. Available from: /doi/pdf/10.1094/9780890546383.013
3. Tembo B, Mulenga RM, Sichilima S, M'siska KK, Mwale M, Chikoti PC, et al. Detection and characterization of fungus (*Magnaporthe oryzae* pathotype *Triticum*) causing wheat blast disease on rain-fed grown wheat (*Triticum aestivum* L.) in Zambia. PLoS One. 2020;15(9):e0238724.
4. Ceresini PC, Castroagudín VL, Rodrigues FÁ, Rios JA, Eduardo Aucique-Pérez C, Moreira SI, et al. Wheat blast: past, present, and future. Annu Rev Phytopathol. 2018;56(1):427–56.
5. Singh PK, Gahtyari NC, Roy C, Roy KK, He X, Tembo B, et al. Wheat blast: A disease spreading by intercontinental jumps and its management strategies. Front Plant Sci [Internet]. 2021 Jul 23;12:710707. Available from: https://www.frontiersin.org/articles/10.3389/fpls.2021.710707/full
6. Bariana HS, McIntosh RA. Cytogenetic studies in wheat. XV. Location of rust resistance genes in VPM1 and their genetic linkage with other disease resistance genes in chromosome 2A. Genome. 1993;36(3):476–82.
7. Cruz CD, Peterson GL, Bockus WW, Kankanala P, Dubcovsky J, Jordan KW, et al. The 2NS translocation from *Aegilops ventricosa* confers resistance to the *Triticum* pathotype of *Magnaporthe oryzae*. Crop Sci. 2016 May 1;56(3):990–1000.
8. Cruppe G, Cruz CD, Peterson G, Pedley K, Asif M, Fritz A, et al. Novel sources of wheat head blast resistance in modern breeding lines and wheat wild relatives. Plant Dis. 2020;104:35–43.

9. He X, Gupta V, Bainsla NK, Chawade A, Singh PK. Breeding for wheat blast resistance. Wheat Blast. 2020;163–74. P. Kashyap, S. Kumar and G.P. Singh (Eds.). Pages 163-174. CRC Press, Boca Raton, FL, USA

10. Islam MT, Nago C, Yoshioka M, Vy TTP, Tosa Y, Asuke S. Identification of *Rmg11* in tetraploid wheat as a new blast resistance gene with tolerance to high temperature. Phytopathology®. 2024;114(8):1878–83.

11. He X, Kabir MR, Roy KK, Anwar MB, Xu K, Marza F, et al. QTL mapping for field resistance to wheat blast in the Caninde# 1/Alondra population. Theor Appl Genet. 2020;133:2673–83.

12. Ferreira JR, Torres GAM, Consoli L, Camilotti GA, Scagliusi SMM, Nhani Jr A, et al. Quantitative trait loci conferring blast resistance in hexaploid wheat at adult plant stage. Plant Pathol. 2021;70(1):100–9.

13. Juliana P, He X, Kabir MR, Roy KK, Anwar MB, Marza F, et al. Genome-wide association mapping for wheat blast resistance in CIMMYT's international screening nurseries evaluated in Bolivia and Bangladesh. Sci Rep. 2020;10(1):15972.

14. Cruppe G, Silva P, Lemes da Silva C, Peterson G, Pedley KF, Cruz CD, et al. Genome-wide association reveals limited benefits of pyramiding the 1B and 1D loci with the $2N^{v}S$ translocation for wheat blast control. Crop Sci. 2021;61(2):1089–103.

15. He X, Juliana P, Kabir MR, Roy KK, Islam R, Marza F, et al. Screening and mapping for head blast resistance in a panel of CIMMYT and South Asian bread wheat germplasm. Front Genet. 2021;12:679162.

16. Roy C, Juliana P, Kabir MR, Roy KK, Gahtyari NC, Marza F, et al. New genotypes and genomic regions for resistance to wheat blast in south Asian germplasm. Plants. 2021;10(12):2693.

17. He X, Kabir MR, Roy KK, Marza F, Chawade A, Duveiller E, et al. Genetic dissection for head blast resistance in wheat using two mapping populations. Heredity (Edinb).

2022;128(6):402–10.

18. Phuke RM, He X, Juliana P, Kabir MR, Roy KK, Marza F, et al. Identification of genomic regions and sources for wheat blast resistance through GWAS in Indian wheat genotypes. Genes (Basel). 2022;13(4):596.

19. Wu L, He X, Kabir MR, Roy KK, Anwar MB, Marza F, et al. Genetic sources and loci for wheat head blast resistance identified by genome-wide association analysis. Crop J. 2022;10(3):793–801.

20. Cruppe G, Lemes da Silva C, Lollato RP, Fritz AK, Kuhnem P, D. Cruz C, et al. QTL pyramiding provides marginal improvement in 2NvS-based wheat blast resistance. Plant Dis. 2023;107(8):2407–16.

21. Goddard R, Steed A, Chinoy C, Ferreira JR, Scheeren PL, MacIel JLN, et al. Dissecting the genetic basis of wheat blast resistance in the Brazilian wheat cultivar BR 18-Terena. BMC Plant Biol. 2020;20(1):1–15.

22. He X, Li C, Kishii M, Asuke S, Kabir MR, Roy KK, et al. A novel quantitative trait locus on chromosome 7D derived from *Aegilops tauschii* confers moderate field resistance to wheat blast. Phytopathology® [Internet]. 2025 Mar 17;115(6):659–65. Available from: https://doi.org/10.1094/PHYTO-01-25-0018-R

23. DAE. Annual Report 2023-2024 [Internet]. Department of Agricultural Extension, MoA, Dhaka, Bangadesh; 2024. Available from: www.dae.gov.bd

24. Malaker PK, Barma NCD, Tiwari TP, Collis WJ, Duveiller E, Singh PK, et al. First Report of Wheat Blast Caused by *Magnaporthe oryzae* Pathotype *triticum* in Bangladesh. https://doi.org/101094/PDIS-05-16-0666-PDN [Internet]. 2016 Aug 22 [cited 2022 Sep 18];100(11):2330–2330. Available from: https://apsjournals.apsnet.org/doi/10.1094/PDIS-05-16-0666-PDN

25. BWMRI. Annual Research Report 2023-24 [Internet]. Bangladesh Wheat and Maize Research Institute, Dinajpur, Bangladesh; 2025. Available from: www.bwmri.gov.bd

26. Barma NCD, Hossain A, Hakim MA, Mottaleb KA, Alam MA, Reza MMA, et al. Progress and challenges of wheat production in the era of climate change: a Bangladesh perspective. Wheat Prod Chang Environ Responses, Adapt Toler. 2019;615–79.

27. Castroagudín VL, Ceresini PC, de Oliveira SC, Reges JTA, Maciel JLN, Bonato AL V, et al. Resistance to QoI fungicides is widespread in Brazilian populations of the wheat blast pathogen Magnaporthe oryzae. Phytopathology. 2015;105(3):284–94.

28. Sakaguchi S, Sugino T, Tsumura Y, Ito M, Crisp MD, Bowman DMJS, et al. High-throughput linkage mapping of Australian white cypress pine (*Callitris glaucophylla*) and map transferability to related species. Tree Genet Genomes. 2015;11:1–12.

29. Martin M. Cutadapt removes adapter sequences from high-throughput sequencing reads. EMBnet J. 2011;17(1):10–2.

30. Zhu T, Wang L, Rimbert H, Rodriguez JC, Deal KR, De Oliveira R, et al. Optical maps refine the bread wheat *Triticum aestivum* cv. Chinese Spring genome assembly. Plant J [Internet]. 2021 [cited 2021 Jun 9]; Available from: https://onlinelibrary.wiley.com/doi/full/10.1111/tpj.15289

31. Li H, Durbin R. Fast and accurate short read alignment with Burrows–Wheeler transform. bioinformatics. 2009;25(14):1754–60.

32. Van der Auwera GA, O'Connor BD. Genomics in the cloud: using Docker, GATK, and WDL in Terra. O'Reilly Media; 2020. 496 p.

33. Browning BL, Browning SR. A unified approach to genotype imputation and haplotype-phase inference for large data sets of trios and unrelated individuals. Am J Hum Genet. 2009;84(2):210–23.

34. Helguera M, Khan IA, Kolmer J, Lijavetzky D, Zhong-Qi L, Dubcovsky J. PCR assays for the Lr37-Yr17-Sr38 cluster of rust resistance genes and their use to develop isogenic hard red spring wheat lines. Crop Sci. 2003;43(5):1839–47.

35. Wang Y, Zhang H, Xie J, Guo B, Chen Y, Zhang H, et al. Mapping stripe rust resistance genes by BSR-Seq: YrMM58 and YrHY1 on chromosome 2AS in Chinese wheat lines Mengmai 58 and Huaiyang 1 are Yr17. 2018;

36. Seah S, Bariana H, Jahier J, Sivasithamparam K, Lagudah ES. The introgressed segment carrying rust resistance genes *Yr17*, *Lr37* and *Sr38* in wheat can be assayed by a cloned disease resistance gene-like sequence. Theor Appl Genet. 2001;102:600–5.

37. Anh VL, Anh NT, Tagle AG, Vy TTP, Inoue Y, Takumi S, et al. *Rmg8*, a new gene for resistance to *Triticum* isolates of *Pyricularia oryzae* in hexaploid wheat. Phytopathology. 2015;105(12):1568–72.

38. Asuke S, Morita K, Shimizu M, Abe F, Terauchi R, Nago C, et al. Evolution of wheat blast resistance gene *Rmg8* accompanied by differentiation of variants recognizing the powdery mildew fungus. Nat Plants. 2024;10(6):971–83.

39. Arora S, Steed A, Goddard R, Gaurav K, O'Hara T, Schoen A, et al. A wheat kinase and immune receptor form host-specificity barriers against the blast fungus. Nat Plants. 2023;9(3):385–92.

40. Bradbury PJ, Zhang Z, Kroon DE, Casstevens TM, Ramdoss Y, Buckler ES. TASSEL: Software for association mapping of complex traits in diverse samples. Bioinformatics. 2007;23(19):2633–5.

41. Subramanian B, Gao S, Lercher MJ, Hu S, Chen WH. Evolview v3: a webserver for visualization, annotation, and management of phylogenetic trees. Nucleic Acids Res [Internet]. 2019 Jul 2;47(W1):W270–5. Available from: https://doi.org/10.1093/nar/gkz357

42. He Z, Zhang H, Gao S, Lercher MJ, Chen WH, Hu S. Evolview v2: an online visualization and management tool for customized and annotated phylogenetic trees. Nucleic Acids Res [Internet]. 2016 Jul 8;44(W1):W236–41. Available from: https://doi.org/10.1093/nar/gkw370

43. Tang Y, Liu X, Wang J, Li M, Wang Q, Tian F, et al. GAPIT Version 2: An enhanced integrated tool for genomic association and prediction. Plant Genome.

2016;9(2):plantgenome2015.11.0120.

44. Wang J, Zhang Z. GAPIT Version 3: Boosting Power and Accuracy for Genomic Association and Prediction. Genomics, Proteomics Bioinforma [Internet]. 2021 [cited 2024 Jul 25];19(4):629–40. Available from: https://doi.org/10.1016/j.gpb.2021.08.005

45. Lipka AE, Tian F, Wang Q, Peiffer J, Li M, Bradbury PJ, et al. GAPIT: Genome association and prediction integrated tool. Bioinformatics. 2012;28(18):2397–9.

46. Myers RH, Montgomery DC. A tutorial on generalized linear models. J Qual Technol. 1997;29(3):274–91.

47. Zhang Z, Ersoz1 E, Lai CQQ, Todhunter RJ, Tiwari HK, Gore MA, et al. Mixed linear model approach adapted for genome-wide association studies. Nat Genet [Internet]. 2010;42(4):335–60. Available from: http://dx.doi.org/10.1038/ng.546

48. Yu J, Pressoir G, Briggs WH, Bi IV, Yamasaki M, Doebley JF, et al. A unified mixed-model method for association mapping that accounts for multiple levels of relatedness. Nat Genet. 2006;38(2):203–8.

49. Segura V, Vilhjálmsson BJ, Platt A, Korte A, Seren Ü, Long Q, et al. An efficient multi-locus mixed-model approach for genome-wide association studies in structured populations. Nat Genet [Internet]. 2012;44(7):825–30. Available from: http://dx.doi.org/10.1038/ng.2314

50. Wang Q, Tian F, Pan Y, Buckler ES, Zhang Z. A SUPER powerful method for genome wide association study. PLoS One. 2014;9(9).

51. Liu X, Huang M, Fan B, Buckler ES, Zhang Z. Iterative usage of fixed and random effect models for powerful and efficient genome-wide association studies. Listgarten J, editor. PLOS Genet [Internet]. 2016 Feb 1 [cited 2020 Jan 23];12(2):e1005767. Available from: http://dx.plos.org/10.1371/journal.pgen.1005767

52. Huang M, Liu X, Zhou Y, Summers RM, Zhang Z, Hall J. BLINK: A package for the next level of genome-wide association studies with both individuals and markers in the millions.

Gigascience [Internet]. 2018 [cited 2020 Feb 9];8(2):1–12. Available from: http://orcid.org/0000-0002-5784-9684

53. Yin L, Zhang H, Tang Z, Xu J, Yin D, Zhang Z, et al. rMVP: a memory-efficient, visualization-enhanced, and parallel-accelerated tool for genome-wide association study. Genomics Proteomics Bioinformatics. 2021;19(4):619–28.

54. The University of Pennsylvania. PhenoGram [Internet]. 2018 [cited 2025 Jan 1]. Available from: https://visualization.ritchielab.org/phenograms/plot

55. IWGSC. Shifting the limits in wheat research and breeding using a fully annotated reference genome. Science (80-). 2018;361:661.

56. Islam MT, Kim KH, Choi J. Wheat blast in Bangladesh: The current situation and future impacts. Plant Pathol J [Internet]. 2019 Feb 1 [cited 2025 May 1];35(1):1. Available from: https://pmc.ncbi.nlm.nih.gov/articles/PMC6385656/

57. Kohli MM, Mehta YR, Guzman E, de Viedma L, Cubilla LE. Pyricularia blast-a threat to wheat cultivation. Czech J Genet Plant Breed. 2011;47(SPEC. ISSUE 1):130–4.

58. Maciel JLN, Ceresini PC, Castroagudin VL, Zala M, Kema GHJ, McDonald BA. Population structure and pathotype diversity of the wheat blast pathogen *Magnaporthe oryzae* 25 years after its emergence in Brazil. Phytopathology. 2014;104(1):95–107.

59. Chao S, Zhang W, Akhunov E, Sherman J, Ma Y, Luo MC, et al. Analysis of gene-derived SNP marker polymorphism in US wheat (*Triticum aestivum* L.) cultivars. Mol Breed [Internet]. 2009 Jul 29 [cited 2025 May 1];23(1):23–33. Available from: https://link.springer.com/article/10.1007/s11032-008-9210-6

60. Price AL, Patterson NJ, Plenge RM, Weinblatt ME, Shadick NA, Reich D. Principal components analysis corrects for stratification in genome-wide association studies. Nat Genet. 2006;38(8):904–9.

61. Inoue Y, Vy TTP, Yoshida K, Asano H, Mitsuoka C, Asuke S, et al. Evolution of the wheat

blast fungus through functional losses in a host specificity determinant. Science (80-). 2017;357(6346):80–3.

62. Fukuoka S, Yamamoto SI, Mizobuchi R, Yamanouchi U, Ono K, Kitazawa N, et al. Multiple functional polymorphisms in a single disease resistance gene in rice enhance durable resistance to blast. Sci Rep. 2014;4(1):4550.

63. Yoshioka M, Shibata M, Morita K, Islam MT, Fujita M, Hatta K, et al. Breeding of a near-isogenic wheat line resistant to wheat blast at both seedling and heading stages through incorporation of *Rmg8*. Phytopathology®. 2024;114(8):1843–50.

64. Inoue Y, Vy TTP, Tani D, Tosa Y. Suppression of wheat blast resistance by an effector of *Pyricularia oryzae* is counteracted by a host specificity resistance gene in wheat. New Phytol. 2021;229(1):488–500.

Supporting information

Supplementary Document: GWAS Methods in GAPIT to Identify SNPs for Wheat Blast Resistance

Supplementary Figure 1: Visualization of field phenotype details for wheat blast score in 14 different environments of Bangladesh and Bolivia

Supplementary Figure 2: Manhattan plot for MTA with six different models

Supplementary Figure 3: Variation in Linkage Disequilibrium across wheat chromosomes in the diverse bread wheat germplasm panel, visualized by r^2 and D' values

Supplementary Table 1: List of genotypes showing immune, resistant, or moderately resistant reactions across locations, along with their genetic composition for 2NS, *Rmg1*, *Rmg6*, and *Rmg8*

Supplementary Table 2: Identified QTLs for wheat blast resistance on different wheat chromosomes.

Figure

Click here to access/download;Figure;Final Fig and Table WB Manuscripts.pptx

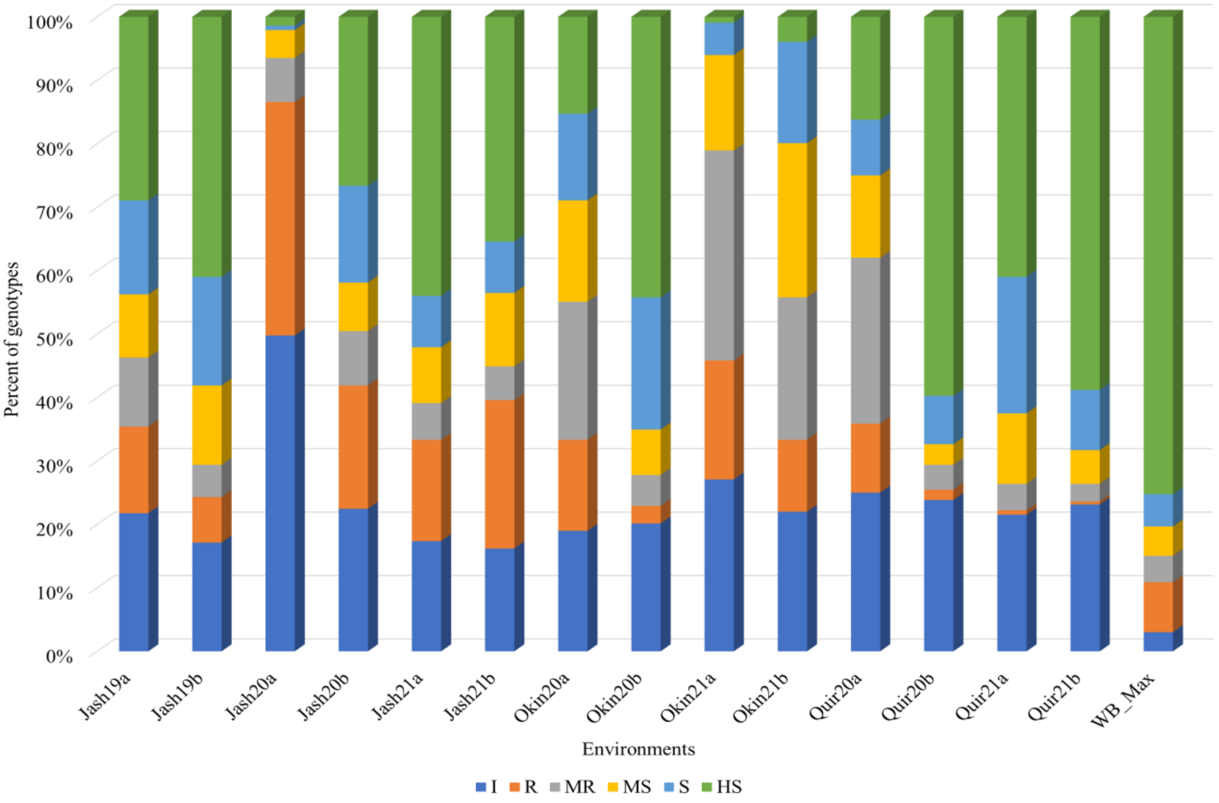

Fig 1: Distribution of wheat blast (WB) severity based on WB index for 432 diverse wheat genotypes evaluated under artificial inoculation across 14 environments in Bangladesh and Bolivia. Reaction categories: I = immune (no symptoms), R = resistant (WB index 1–10%), MR = moderately resistant (11–25%), MS = moderately susceptible (26–40%), S = susceptible (41–60%), and HS = highly susceptible (61–100%)

Table 1: Within-environment analysis of variance for WB Index

Environments	Average WB Index	Df (Genotype)	Df (Sowing Time)	Error Df	F-value (genotype)	F-value (sowing time)
Jashore 19	44.9	449	1	449	3.23***	62.2***
Jashore 20	22	449	1	449	1.68***	396***
Jashore 21	46.7	447	1	442	7.92***	41.7***
Okinawa 20	38	449	1	448	3.21***	229***
Okinawa 21	19.4	448	1	441	1.83***	70.5***
Quirusillas 20	43	449	1	449	2.93***	368***
Quirusillas 21	54.2	447	1	446	8.49***	87.7***

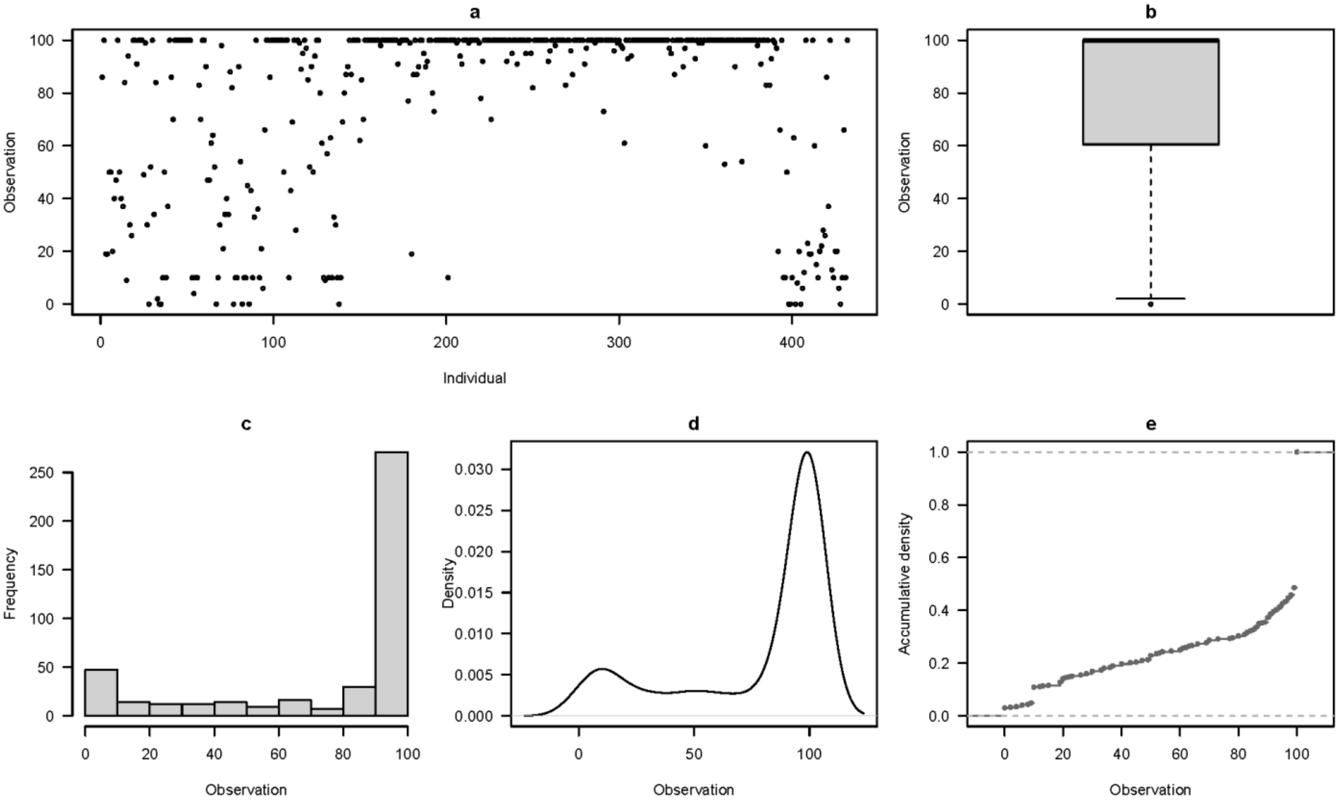

Fig 2. Field performance of the diverse wheat genotypes for wheat blast resistance across experiments. The figure summarizes maximum wheat blast scores pooled from all environments and includes the following visualizations: (a) scatter plot showing individual genotype scores; (b) box plot indicating that most genotypes fall within the upper quartile, suggesting high susceptibility in at least one environment; (c) histogram displaying the distribution of scores; (d) density plot illustrating the distribution pattern; and (e) cumulative density plot highlighting the prevalence of higher wheat blast scores. The wide range of values reflects substantial variability in resistance among the tested genotypes (S1 Fig illustrated for additional details of 14 environments, representing seven growing seasons in three locations).

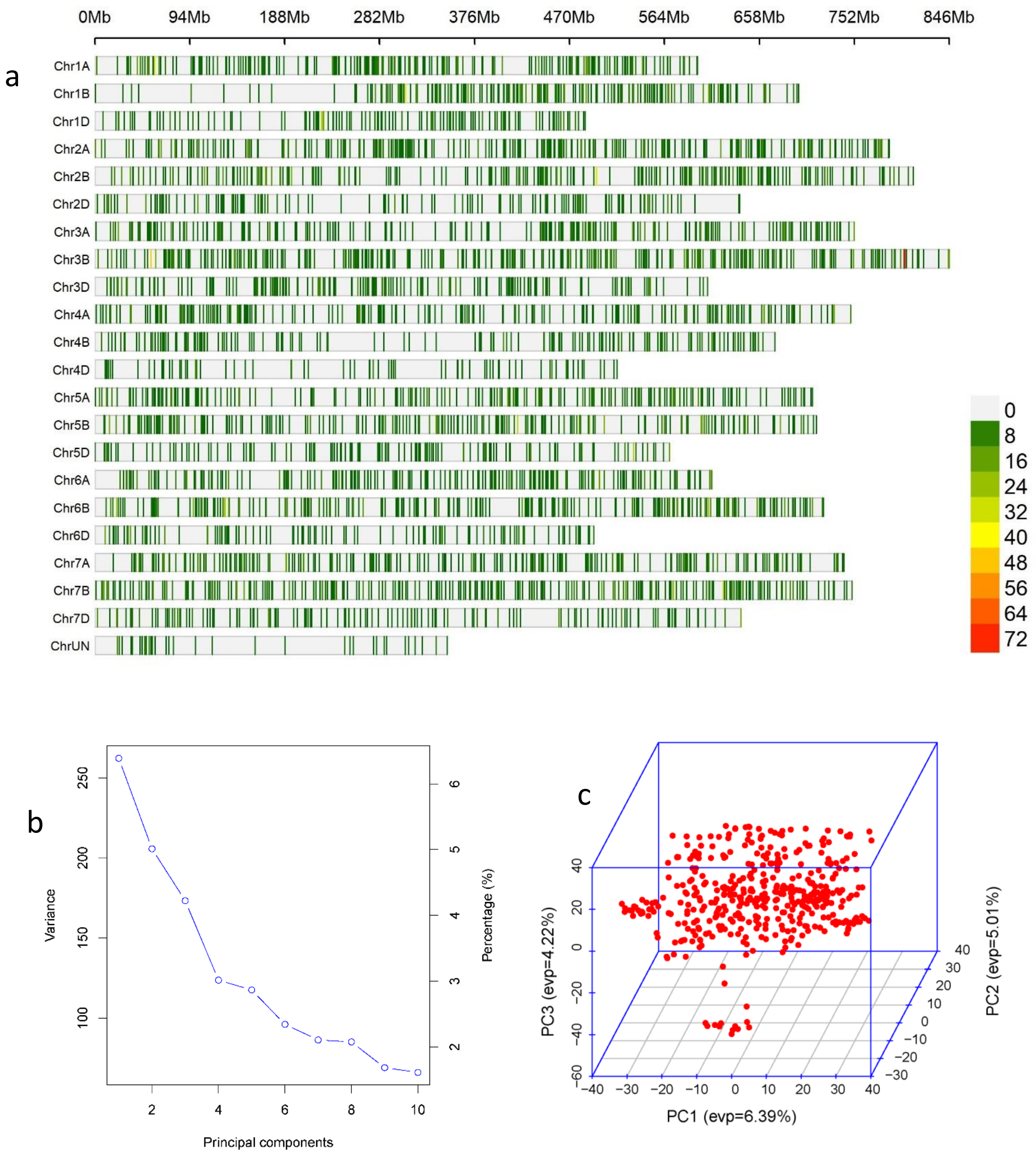

Fig 3: Genomic characteristics and principal component analysis (PCA) based on ddRAD-seq-derived SNP markers across 432 wheat genotypes. (a) Genome-wide marker density plot showing the distribution of SNPs across the wheat chromosomes; (b) Scree plot of eigenvalues indicating the proportion of total genetic variance explained by each principal component, used to determine the number of informative components; and (c) 3D PCA plot illustrating the genetic diversity and clustering patterns among the wheat genotypes based on the top three principal components.

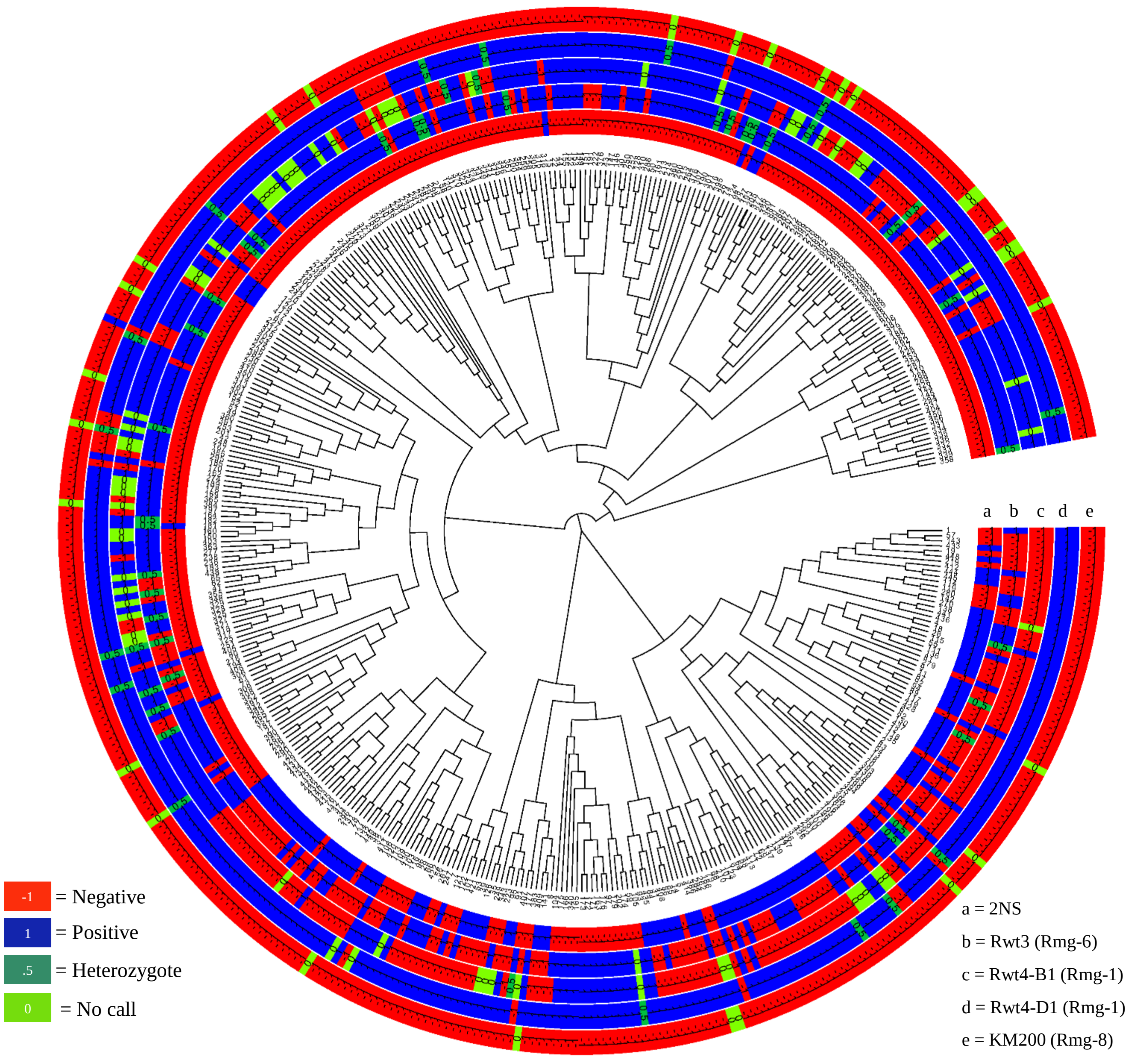

Fig 4: Neighbour-Joining tree based on genetic distances among the 432 bread wheat genotypes, showing the distribution of four *Rwt* (*Rmg*) genes and the 2NS translocation.

Fig 5: Manhattan Plots Showing GWAS Results Using Six Different Models. The plots were drawn based on the pooled highest wheat blast (WB) scores across 14 environments. Individual Manhattan plots for each environment are presented in S2 Fig.

Blink

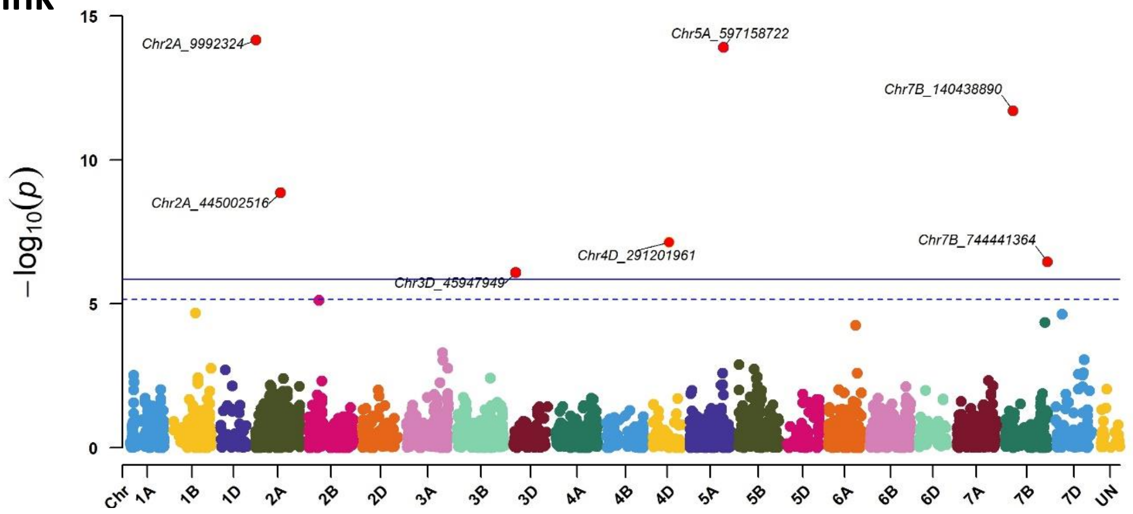

FarmCPU

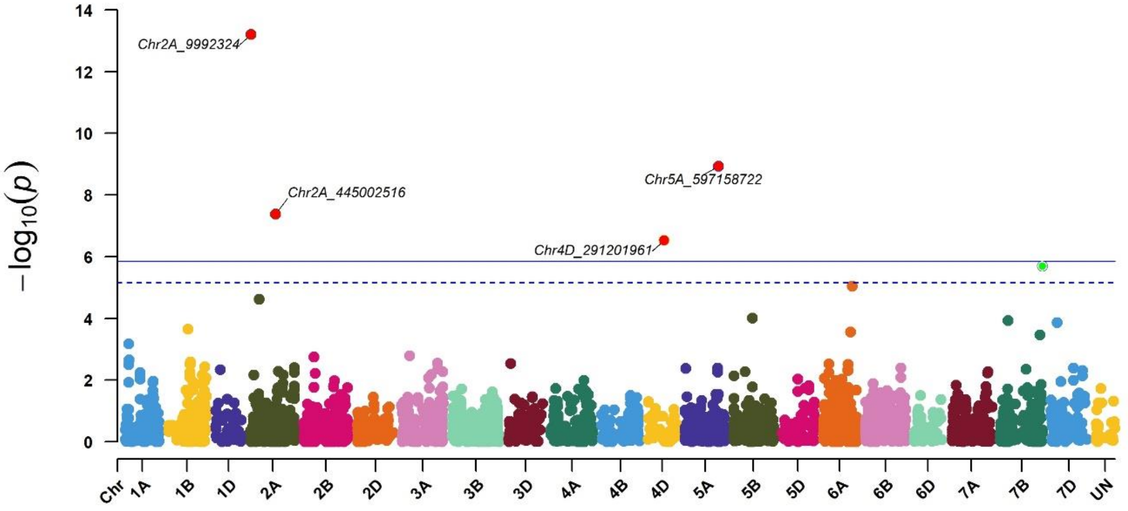

GLM

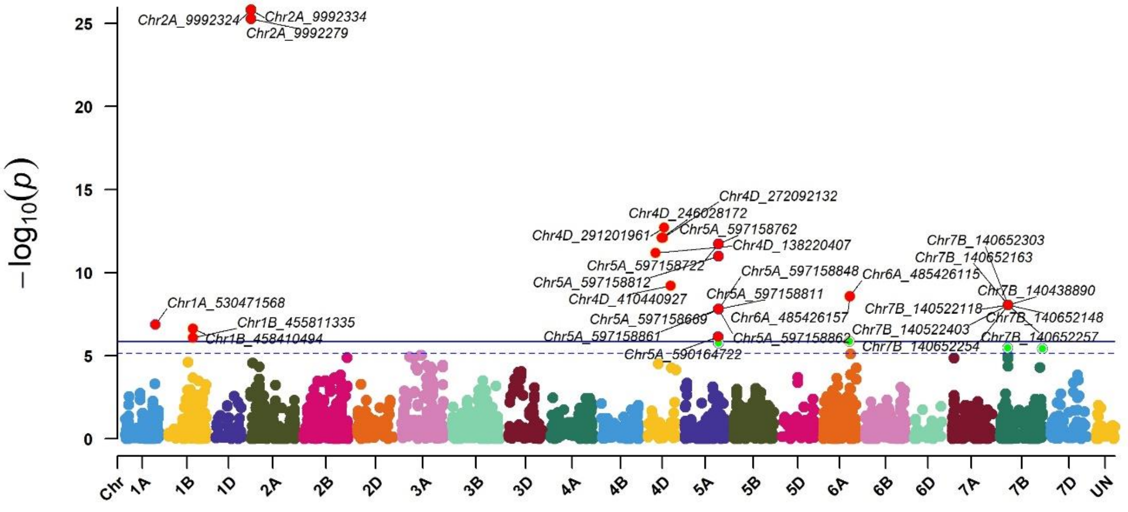

MLM

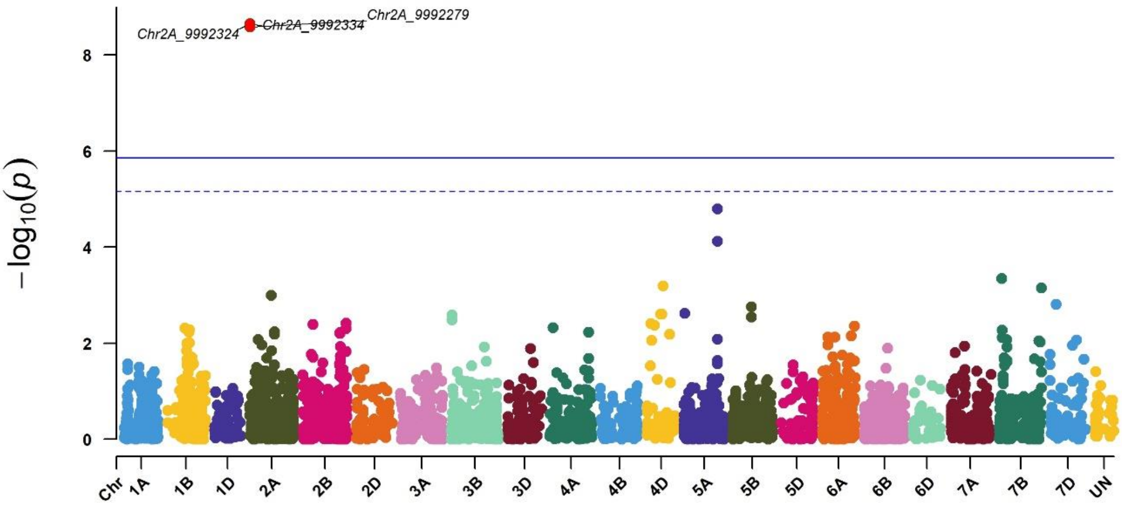

MLMM

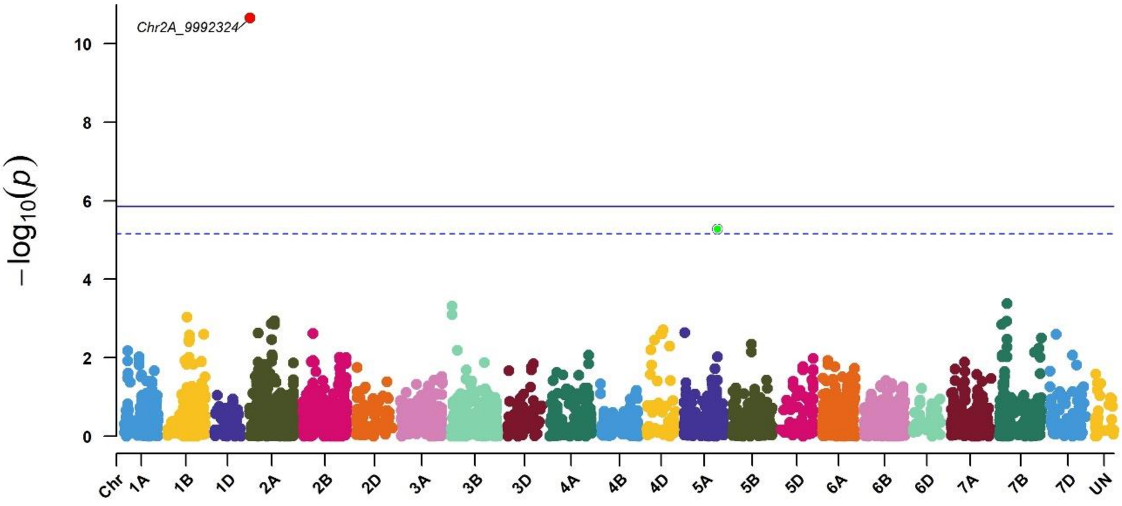

SUPER

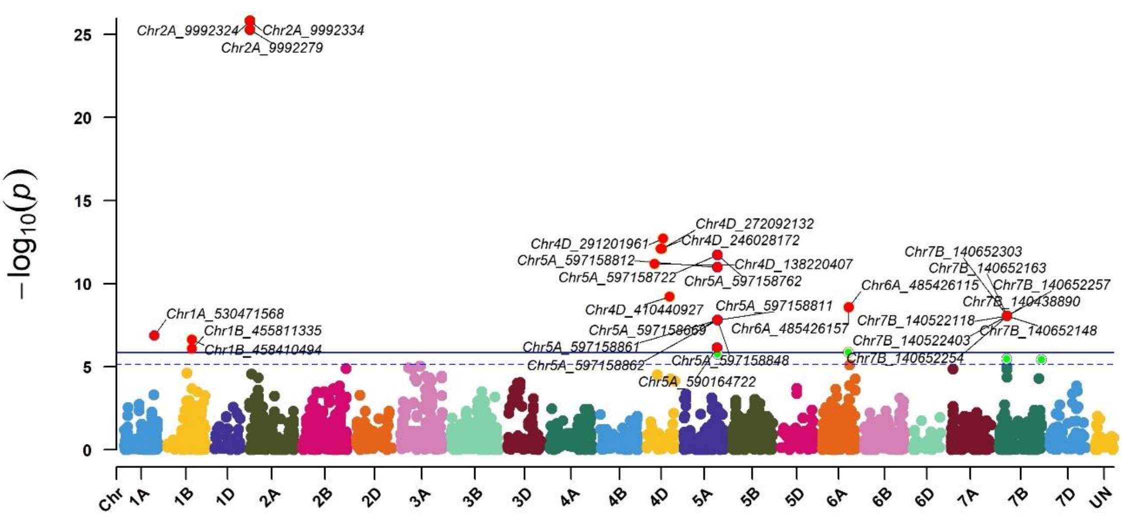

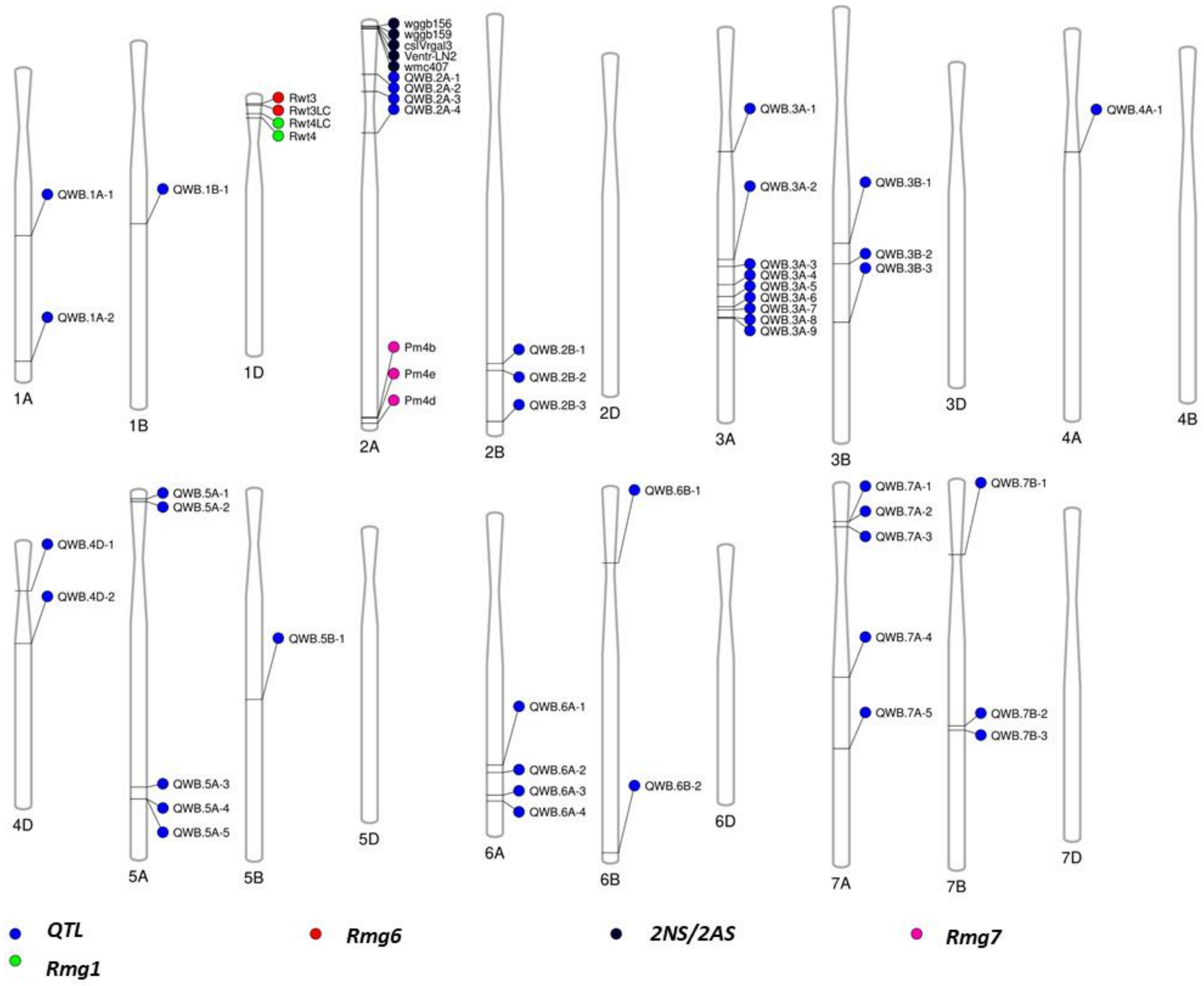

Fig 6: Genome wide QTL distribution for wheat blast resistance identified from the 14 different field experiments using six different models. Integrated wheat blast resistance QTL map constructed using a phenogram-based visualization tool. A total of 45 QTLs associated with wheat blast resistance are displayed in blue dots across the wheat genome. Regions enriched with QTLs are observed particularly on chromosomes 2A, 3A, 3B, 5A, 5B, 6A, 6B, 7A, and 7B. The map also shows the position of several *Rmg* (*Rwt*) genes and the 2NS/2AS translocation.

Click here to access/download
Supporting Information
Supplimentary Document.pdf

Click here to access/download
Supporting Information - Compressed/ZIP File Archive
Supplementary Tables.pdf

Click here to access/download
Supporting Information - Compressed/ZIP File Archive
Supplimentary Figures.pdf
