# Supplementary material for: Field screening and genetic mapping of wheat blast resistance for a panel of common wheat from Bangladesh
Source: PLoS One. 2026 Jun 11;21(6):e0349201. doi: 10.1371/journal.pone.0349201 (PMC13258015; doi:10.1371/journal.pone.0349201)
Supplement: Attachment — Submitted filename: Review_PLOSone_SEL.docx [file pone.0349201.s008.docx]

The study addresses an important topic which is Genetic dissection of wheat blast resistance in Bangladeshi germplasm; but falls short in methodological rigor, interpretation of GWAS results, and clarity regarding QTL definition and independence. The experimental design and statistical analyses do not support the strength of the claims made.

Major Concerns

1. Material description and population structure
   - The mention of “respective parental lines” is ambiguous. It is unclear whether these include hybrid materials or simply refer to the parental lines used to generate the advanced breeding lines and varieties included in the panel. The authors should clarify whether all or only a subset of parental lines were represented. Furthermore, the later reference to “synthetic lines” is inconsistent with the materials description, as no synthetic germplasm was mentioned in the Plant Materials section. This discrepancy should be addressed and the composition of the panel clearly defined.
   - The structure plot shows weak distinction among groups; authors claim clear separation, which is not evident.
   - The PCA cloud contradicts the claim of “distinct clusters,” and the description of synthetic wheat should be supported by pedigree data.
2. Experimental Design
   - The authors treated different planting dates as replications, and the presentation of the ANOVA results (Table 1) is insufficient to support any quantitative inference about genetic variance or heritability. Moreover, the manuscript does not include any variance partitioning or heritability estimates across environments, which are essential for evaluating the consistency of genotype effects and the robustness of the GWAS results. Without within- and across-environment variance component analysis, the statistical strength of the reported associations remains uncertain.
3. Statistical analysis
   - The statistical models used are missing
   - Given the lack of replication, genomic repeatability can be estimated to gauge consistency of marker effects within environments, how relevant each environment can be concluded in such away!
   - The models used for GWAS are mentioned, but the model specification (fixed vs random terms, kinship, structure correction) is missing. No validation or cross-model comparison is presented.
   - A hierarchical clustering of the 14 environments based on the pairwise Pearson correlation coefficients for the disease is missing.
4. Purpose and interpretation of environment-specific GWAS
   - Running GWAS within each environment is acceptable, but the rationale is unclear; is it to capture race-specific resistance, breakdown effects, or local adaptation?
   - The authors could have categorized loci as single-environment QTL (SEQ) versus multi-environment QTL (MEQ) to highlight consistency of associations.
5. QTL definition and independence
   - The criteria which you use for grouping SNP into QTL based on linkage disequilibirum need to be pointed. Especially for introgression they may not be optimal because it will depend on the frequency of a given introgression in a population whether MTAs will be grouped into a single QTL or several QTL.
   - The manuscript lists several QTL but does not clarify how many are independent versus overlapping. Several QTL may be artifacts of LD clustering.
   - The authors need to summarize the QTLs with the corresponding relevant information and then specify the leading marker of the region.
   - The LD thresholds (r² ≥ xx, D′ ≥ xx) are arbitrary; the choice and its implications, especially in regions with introgressions, need justification. The delineation of the QTL is not clear enough, SNPs located on the same chromosome and within which Mb distance were considered as representatives of the same QTL when their LD measured as r^2^ was greater to what threshold? which threshold has been used for SNPs located on different chromosomes if significant SNPs are mismapped (I hope the authors omitted those missmaped ones).
   - For introgressed segments, LD-based grouping may merge or split true QTL incorrectly depending on allele frequency and haplotype structure.
6. Missing PVE and dominance information
   - The proportion of variance explained (PVE) by each QTL is not described. It is unclear how environmental data were incorporated into PVE and degree-of-dominance calculations.
   - Substantial differences between environments suggest that per-environment effect sizes should have been visualized (e.g. heatmap of overlapping SNPs).
