# Supplementary material for: Field screening and genetic mapping of wheat blast resistance for a panel of common wheat from Bangladesh
Source: PLoS One. 2026 Jun 11;21(6):e0349201. doi: 10.1371/journal.pone.0349201 (PMC13258015; doi:10.1371/journal.pone.0349201)
Supplement: Attachment — Submitted filename: Response to Reviewers.docx [file pone.0349201.s009.docx]

Thank you for submitting your manuscript to PLOS ONE. After careful consideration, we feel that it has merit but does not fully meet PLOS ONE’s publication criteria as it currently stands. Therefore, we invite you to submit a revised version of the manuscript that addresses the points raised during the review process.

Please submit your revised manuscript by Dec 22 2025 11:59PM. If you will need more time than this to complete your revisions, please reply to this message or contact the journal office at [plosone@plos.org](mailto:plosone@plos.org). When you're ready to submit your revision, log on to <https://www.editorialmanager.com/pone/> and select the 'Submissions Needing Revision' folder to locate your manuscript file.

Please include the following items when submitting your revised manuscript:

- A rebuttal letter that responds to each point raised by the academic editor and reviewer(s). You should upload this letter as a separate file labeled 'Response to Reviewers'.
- A marked-up copy of your manuscript that highlights changes made to the original version. You should upload this as a separate file labeled 'Revised Manuscript with Track Changes'.
- An unmarked version of your revised paper without tracked changes. You should upload this as a separate file labeled 'Manuscript'.

If you would like to make changes to your financial disclosure, please include your updated statement in your cover letter. Guidelines for resubmitting your figure files are available below the reviewer comments at the end of this letter.

If applicable, we recommend that you deposit your laboratory protocols in [protocols.io](http://protocols.io/) to enhance the reproducibility of your results. Protocols.io assigns your protocol its own identifier (DOI) so that it can be cited independently in the future. For instructions see: [https://journals.plos.org/plosone/s/submission-guidelines#loc-laboratory-protocols](https://track.editorialmanager.com/CL0/https:%2F%2Fjournals.plos.org%2Fplosone%2Fs%2Fsubmission-guidelines%23loc-laboratory-protocols/1/010f019a5efa13c7-7b7a1957-aeaa-42b3-aa6b-170462347b07-000000/L0HCE7vxJZwUFDS2VxSfu68Fgf_oovX09Oca5o-rC_Y=236). Additionally, PLOS ONE offers an option for publishing peer-reviewed Lab Protocol articles, which describe protocols hosted on [protocols.io](http://protocols.io/). Read more information on sharing protocols at [https://plos.org/protocols?utm_medium=editorial-email&utm_source=authorletters&utm_campaign=protocols](https://track.editorialmanager.com/CL0/https:%2F%2Fplos.org%2Fprotocols%3Futm_medium=editorial-email%26utm_source=authorletters%26utm_campaign=protocols/1/010f019a5efa13c7-7b7a1957-aeaa-42b3-aa6b-170462347b07-000000/7-Wg8bhu3JIyAnXTJHV5cA_jBBw4Zst-5UNeD1fFxzo=236).

Author Response:

Thank you for offering us to publish protocol. We do not have novel laboratory protocol to publish for this manuscript.

We look forward to receiving your revised manuscript.

Kind regards,

Wuletaw Tadesse
Academic Editor
PLOS ONE

Journal Requirements:

When submitting your revision, we need you to address these additional requirements.

1. Please ensure that your manuscript meets PLOS ONE's style requirements, including those for file naming. The PLOS ONE style templates can be found at

<https://journals.plos.org/plosone/s/file?id=wjVg/PLOSOne_formatting_sample_main_body.pdf> and

[https://journals.plos.org/plosone/s/file?id=ba62/PLOSOne_formatting_sample_title_authors_affiliations.pdf](https://track.editorialmanager.com/CL0/https:%2F%2Fjournals.plos.org%2Fplosone%2Fs%2Ffile%3Fid=ba62%2FPLOSOne_formatting_sample_title_authors_affiliations.pdf/1/010f019a5efa13c7-7b7a1957-aeaa-42b3-aa6b-170462347b07-000000/eMlyHms_aWVag2FFKW8MhLg2ARzuCjPoG9qIufHuCOw=236).

Author Response:

The revised manuscript has reorganised as per the PLOS ONE’s style requirement.

2. Thank you for stating the following financial disclosure:

“Accelerating Genetic Gain (AGG) in Maize and Wheat Project Grant INV-003439 funded by the Bill and Melinda Gates Foundation (BMGF), the Foreign and Commonwealth Development Office (FCDO)”

Please state what role the funders took in the study.  If the funders had no role, please state: "The funders had no role in study design, data collection and analysis, decision to publish, or preparation of the manuscript."

If this statement is not correct you must amend it as needed.

Please include this amended Role of Funder statement in your cover letter; we will change the online submission form on your behalf.

3. In the online submission form you indicate that your data is not available for proprietary reasons and have provided a contact point for accessing this data. Please note that your current contact point is a co-author on this manuscript. According to our Data Policy, the contact point must not be an author on the manuscript and must be an institutional contact, ideally not an individual. Please revise your data statement to a non-author institutional point of contact, such as a data access or ethics committee, and send this to us via return email. Please also include contact information for the third party organization, and please include the full citation of where the data can be found.

4. Thank you for stating the following in the Acknowledgments Section of your manuscript:

“Financial supports from the Australian Centre for International Agricultural Research, Australia, One CGIAR Initiatives ABI and PHI, the Ministry of Agriculture, Forestry and Fisheries of Japan, The Krishi Gobeshona Foundation-Bangladesh, and Accelerating Genetic Gain (AGG) in Maize and Wheat Project Grant INV-003439 funded by the Bill and Melinda Gates Foundation (BMGF), the Foreign and Commonwealth Development Office (FCDO) are gratefully acknowledged. “

We note that you have provided additional information within the Acknowledgements Section that is not currently declared in your Funding Statement. Please note that funding information should not appear in the Acknowledgments section or other areas of your manuscript. We will only publish funding information present in the Funding Statement section of the online submission form.

Please remove any funding-related text from the manuscript and let us know how you would like to update your Funding Statement. Currently, your Funding Statement reads as follows:

“Accelerating Genetic Gain (AGG) in Maize and Wheat Project Grant INV-003439 funded by the Bill and Melinda Gates Foundation (BMGF), the Foreign and Commonwealth Development Office (FCDO)”

Please include your amended statements within your cover letter; we will change the online submission form on your behalf.

Author Response:

We removed funding information from Acknowledgement section, and we , and we have updated the Acknowledgement section in the revised manuscript.

5. If the reviewer comments include a recommendation to cite specific previously published works, please review and evaluate these publications to determine whether they are relevant and should be cited. There is no requirement to cite these works unless the editor has indicated otherwise. 

Additional Editor Comments (if provided):

Dear Authors,
A major revision is recommended from reviewers. Please revise your manuscript carefully and submit accordingly. Attached is the comments of the 3 reviewers.
 **Reviewers' comments:**

**Reviewer #1:** This manuscript presents a field-based evaluation and genome-wide association study (GWAS) for wheat blast resistance using a diverse panel of 432 common wheat accessions genotyped through ddRAD-seq. The topic is highly relevant and timely for wheat breeding programs, particularly in Bangladesh and other blast-endemic regions. The integration of field phenotyping and genotyping provides valuable insights into the genetic architecture of resistance, including confirmation of the well-known 2NS/2AS translocation and identification of additional loci across the genome.

While the experimental design is sound and the results are promising, the manuscript would benefit from clarification and improvement in several key areas.

Introduction
The introduction would be coherent and more engaging if it began with a general overview of wheat production and its importance in Bangladesh, followed by a concise description of wheat blast disease, its impact, and its specific relevance to the region. This could then transition into a discussion of resistance mechanisms, including the 2NS/2AS translocation, known resistance genes such as the Rmg loci, and previously identified QTLs.
It is recommended to include a brief summary of previously reported QTLs and SNPs associated with wheat blast resistance identified through GWAS or QTL mapping.
The objectives section would benefit from a clearer statement emphasizing the novelty and originality of the current study, and how it differs from and advances beyond previous investigations.

Author Response:

We thank the reviewer for the thoughtful recommendations to improve the structure and clarity of the Introduction.

In the revised manuscript, the Introduction has been substantially reorganized to enhance coherence and narrative flow. It now begins with the importance of wheat production and food security in Bangladesh, followed by a concise overview of wheat blast, its rapid global spread, and its specific agronomic and economic relevance to the region. The discussion then transitions to resistance mechanisms, including the 2NS/2AS translocation, major seedling resistance genes (e.g., Rmg loci), and previously identified QTL from both bi-parental and GWAS studies.

To address the reviewer’s recommendation, we have incorporated a structured summary of previously reported QTL and significant SNP associations for wheat blast resistance. This synthesis highlights the predominant role of the 2NS segment across studies, as well as the generally minor and environment-dependent effects of non-2NS loci reported in South American and South Asian germplasm.

The objectives section has been revised to more clearly articulate the originality and advancement of the present study. Unlike many prior investigations that focused primarily on CIMMYT-derived or South American germplasm, or relied heavily on controlled-condition screening, our study evaluates a diverse panel of Bangladeshi wheat genotypes under multi-environment field conditions representing natural epidemic variability. Furthermore, the use of a multi-model GWAS framework combined with LD-based locus delineation and cross-environment consistency criteria provides a more comprehensive assessment of resistance architecture relevant to local breeding programs.

These revisions clarify how the present study builds upon and extends prior South Asian GWAS efforts by focusing on locally adapted germplasm, environment-specific resistance dynamics, and identification of candidate loci beyond the widely deployed 2NS source.

Materials and Methods
In the description “1 m double rows separated by 20 cm spacing in all three locations, and no replication was arranged within each sowing,” please specify the exact experimental design and briefly justify the statistical rationale for its use.
The use of the WB index (incidence × severity) as the main phenotypic trait is appropriate and common in wheat blast studies. However, since the WB index may conflate genetic resistance with phenological escape, it would be helpful to explain why complementary traits, such as days to heading, spike sterility, and yield, were not recorded.
Please confirm whether a standardized wheat blast scoring protocol was used, for example that described by Cruz et al. (2016): Cruz, C. D., W. W. Bockus, J. P. Stack, B. Valent, J. N. Maciel, and G. L. Peterson (2016). A Standardized Inoculation Protocol to Test Wheat Cultivars for Reaction to Head Blast Caused by Magnaporthe oryzae (Triticum pathotype). Plant Health Progress, 17(3), 186–187. <https://doi.org/10.1094/PHP-BR-16-0041>.
The authors are encouraged to justify the choice of sampling (10 spikes per plot at 21 days) and add references to standard methods.
The results section currently lacks estimates of trait heritability and genotype × environment (G×E) variance components. It is recommended to fit a multi-environment mixed model (e.g., including genotype, environment, and G×E as random effects) and to report broad-sense heritability (H²) within and across environments.
The statement that Manhattan plots were generated using “the pooled highest WB scores across 14 environments” requires clarification. Please specify whether GWAS was performed using BLUEs, BLUPs, or another aggregated phenotype.

Author Response:

We thank the reviewer for these helpful suggestions and provide the following clarifications.

The field trials were conducted using an unreplicated design, with different sowing dates treated as temporal replications within each environment. This approach has been commonly used in large-scale wheat blast screening nurseries under artificial inoculation, where logistical constraints limit full replication while allowing evaluation of a large number of genotypes [1,2].

The wheat blast (WB) index (incidence × severity) was used as the primary phenotypic trait because it is a widely accepted metric for quantifying head blast under field epidemic conditions[2–4]. We acknowledge the potential confounding effect of phenological escape. To address this, days to heading and plant height were recorded for all genotypes and are now reported in the revised manuscript to support interpretation of disease response independent of maturity differences. Artificial inoculation targeting to each genotype’s anthesis and mist irrigation were implemented to minimize escape effects due to asynchronous flowering.

A standardized inoculation and scoring protocol consistent with Cruz et al. (2016) and subsequent CIMMYT-led studies was followed. Disease assessment based on 10 tagged spikes per plot at 21 days after anthesis has been widely applied and shown to provide stable estimates of wheat blast severity under field conditions and the reference were added in the revised manuscript.

In response to the reviewer’s recommendation, variance components were estimated using mixed linear models, and broad-sense heritability on a genotype-mean basis ($H_{gm}^{2}$) is now reported (Table 1). Genotype × environment interaction was evaluated through multi-environment analysis, including within-environment ANOVA and likelihood ratio testing implemented in the Metan R package. These additions strengthen quantitative inference regarding the consistency of genotype performance [5,6].

Regarding the GWAS phenotype, analyses were conducted using the maximum WB index per genotype across the 14 environments to capture resistance performance under severe epidemic conditions. The previous wording has been clarified in the revised manuscript. While BLUEs or BLUPs are commonly used in multi-environment analyses, the use of maximum disease expression was considered appropriate given the highly variable and epidemic-driven nature of wheat blast pressure, and aligns with the study objective of identifying loci effective under intense and variable disease conditions.

All relevant sections of the Materials and Methods and Results have been revised accordingly to improve transparency and statistical rigor.

Results
Lines 219–221: The site “Jash20a” is described as both a “low-pressure” and a “highly conducive” environment. Please verify and correct this discripancy.
Line 299: Please indicate the exact numerical threshold used after Bonferroni correction.
Line 311: Correct the phrase “form GWAS” to “from GWAS”.

Author Response:

We thank the reviewer for these helpful comments on result section. We have address them as follows:

The discrepancy in Lines 219–221 has been corrected. The reference to a highly conducive environment was a typographical error; the environment with high disease pressure is Jash21a, not Jash20a.

The exact Bonferroni-corrected threshold has now been clearly stated in the Results section as α = 0.05/7,211 SNPs (p = 6.93 × 10⁻⁶; −log₁₀(p) = 5.16).

The typographical error in Line 311 has been corrected from “form GWAS” to “from GWAS.”

In addition to Supplementary Figure 3, consider including a genome-wide LD-decay curve (average r² vs. physical distance) and report the LD-decay threshold, as this would add useful genomic context.

Author Response:

We thank the reviewer for this valuable suggestion. We have now included a genome-wide LD-decay curve (average r² vs. physical distance) as Figure S4. Background LD was estimated using the 95th percentile of inter-chromosomal marker r² values. Genome-wide LD decayed below this threshold at approximately 144 Kb, indicating relatively rapid LD decay and providing genomic context for QTL interval definition. The Methods and Results sections have been updated accordingly.

For each reported lead SNP/QTL, please provide estimates of allelic effect size and the percentage of phenotypic variance explained (R²), as these values are essential for breeders to assess practical significance.

Author Response:
We thank the reviewer for this important suggestion. In response, we have now included, for each reported lead SNP/QTL, the estimated additive allelic effect size (β) and the corresponding proportion of phenotypic variance explained (R²) in Table 3. Accordingly, the Results section has been corrected where appropriate.

Discussion
Line 349: Please justify the choice of the ddRAD-seq approach, particularly given that after filtering, only 7,211 SNPs remained from an initial 82,948.
Discuss how the lower SNP density may limit fine-scale mapping resolution.
Line 359: The discussion would benefit from a more explicit comparison between the QTLs identified in this study and those reported in previous GWAS and mapping studies on wheat blast resistance (e.g., Cruppe et al., 2023; Phuke et al., 2022; Goddard et al., 2020; He et al., 2021; Juliana et al., 2020). At present, it is not entirely clear which of the Qwb.cim loci correspond to previously known regions, such as the 2NS/2AS translocation, and which may represent novel genomic regions.

Author Response:

We thank the reviewer for this comment. The Discussion has been revised (Line 349) to justify the use of ddRAD-seq as a cost-effective genotyping approach for a large and diverse wheat panel and to clarify the stringent SNP filtering process that resulted in 7,211 high-quality markers, with appropriate references added.

We appreciate the suggestion and have also revised the Discussion (Line 359) to provide a clearer comparison between the QTLs identified in this study and those reported previously. We indicate that several Qwb.cim loci correspond to known resistance regions, including the 2NS/2AS translocation on chromosome 2A, while others are physically distinct from previously reported loci. The discussion focuses on the linkage and distribution of known resistance genes (e.g., 2NS and selected Rmg loci), as illustrated in Figure 8, which was used as the primary basis for this discussion.

**Reviewer #2:** Dear Editor,
The authors did field screening and genetic mapping of wheat blast using six different model analysis. The association analysis identified about 1,121 significant markers and grouped 45 QTLS. The study is important for the scientific community.
I have some queries and suggestions kindly address that
1. The experiment screened 432 genotypes for wheat blast in seven environment’s using two row per genotype and it is not replicated (line # 118). The within-environment error variance could not be estimated, because experimental units were not replicated. Differences between genotypes may be confounded with micro environment variation. The GWAS results based on non-replicated are not reliable enough. The authors should give clear evidence how to resolve such issues.

Author Response:

We thank the reviewer for this important comment and acknowledge the limitations associated with the unreplicated field design.

The wheat blast evaluations were conducted using an unreplicated screening design, which is frequently used in large-scale disease resistance nurseries where space, inoculation logistics, and the need to evaluate a large number of genotypes constrain full replication [2,4]. We recognize that, under such a design, within-environment error variance cannot be directly estimated, and that micro-environmental variation may influence single-environment observations.

To reduce the impact of these limitations, genotypes were evaluated across seven independent environments, including multiple locations and sowing times, providing temporal replication and allowing resistance responses to be assessed under diverse epidemic conditions. In addition, artificial inoculation and mist irrigation were used to promote relatively uniform disease pressure within environments, which is standard practice in wheat blast phenotyping [7].

For GWAS, phenotypic values were derived from multi-environment observations rather than single-environment measurements, and analyses were conducted using multiple GWAS models incorporating population structure and kinship. Marker–trait associations were interpreted conservatively, emphasizing consistency across environments and models and support from local LD structure, rather than reliance on single-environment effects. Similar approaches have been applied in previous wheat blast GWAS studies conducted under unreplicated or partially replicated screening conditions[2,5].

We explicitly acknowledge in the *Concluding Remarks and Recommendations* section of the manuscript that “these loci represent a foundation for future efforts in fine mapping, functional validation, and candidate gene discovery,” thereby indicating that the identified loci should be regarded as putative resistance regions that warrant validation through higher-resolution mapping and further studies.

2. Candidate genes need to be explored in different database.

Author Response:

We thank the reviewer for this valuable suggestion. While we agree that exploring candidate genes across multiple databases would provide additional insights, the primary objective of our current study is to identify novel putative resistance regions in the wheat genome. These regions represent a robust foundation for future discovery of candidate genes. Comprehensive characterization, including fine mapping, functional validation, and database-driven exploration of candidate genes, is planned as a follow-up to this work. This approach allows us to first establish a reliable genomic framework, which will enhance the accuracy and efficiency of subsequent gene-level analyses.

**Reviewer #3:** The study addresses an important topic which is Genetic dissection of wheat blast resistance in Bangladeshi germplasm; but falls short in methodological rigor, interpretation of GWAS results, and clarity regarding QTL definition and independence. The experimental design and statistical analyses do not support the strength of the claims made.
Major Concerns
1. Material description and population structure
- The mention of “respective parental lines” is ambiguous. It is unclear whether these include hybrid materials or simply refer to the parental lines used to generate the advanced breeding lines and varieties included in the panel. The authors should clarify whether all or only a subset of parental lines were represented. Furthermore, the later reference to “synthetic lines” is inconsistent with the materials description, as no synthetic germplasm was mentioned in the Plant Materials section. This discrepancy should be addressed and the composition of the panel clearly defined.
- The structure plot shows weak distinction among groups; authors claim clear separation, which is not evident.
- The PCA cloud contradicts the claim of “distinct clusters,” and the description of synthetic wheat should be supported by pedigree data.

Author Response:

We thank the reviewer for this comment. The “respective parental lines” in our panel represent a subset of parental lines used in the breeding program that contributed directly to the advanced lines included in this study. Additionally, several entries are derived from synthetic wheat (*Aegilops squarrosa* crosses), forming a distinct cluster in our analysis. We revised the Plant Materials section to clearly describe the panel composition, including these synthetic derivatives.

We acknowledge that the majority of genotypes show overlapping clustering, which is expected due to shared ancestry in breeding populations. However, the 3D structure plot (Figure 3c) reveals a small, distinct cluster clearly separated from the main group. This cluster corresponds to synthetic wheat derivatives, originating from *Aegilops squarrosa* crosses. Additionally, the overall clustering pattern aligns with the distribution of four Rwt (Rmg) genes and the 2NS translocation, supporting meaningful genetic differentiation among the panel.

Synthetic wheat is already supported by the pedigree data (*Aegilops squarrosa* derivatives) .

2. Experimental Design
- The authors treated different planting dates as replications, and the presentation of the ANOVA results (Table 1) is insufficient to support any quantitative inference about genetic variance or heritability. Moreover, the manuscript does not include any variance partitioning or heritability estimates across environments, which are essential for evaluating the consistency of genotype effects and the robustness of the GWAS results. Without within- and across-environment variance component analysis, the statistical strength of the reported associations remains uncertain.

Author Response:

We thank the reviewer for raising important concerns regarding experimental design, variance partitioning, and the strength of statistical inference.

We acknowledge that the field evaluation followed an unreplicated screening design within each environment, where different sowing dates were treated as temporal blocks rather than true biological replications. Within-environment replication was constrained by the large panel size and the operational requirements of uniform artificial inoculation and mist irrigation under high disease pressure. Such designs are commonly implemented in large-scale disease resistance screening programs to balance panel size with experimental feasibility[2,4].

In response to the reviewer’s concern, we have strengthened the statistical analysis in the revised manuscript. Specifically, variance components were estimated using mixed linear models, and Heritability of means ($H_{gm}^{2}$) is now reported (revised Table 2). These estimates incorporate genotype and genotype × environment components across the multi-environment dataset, thereby providing a quantitative assessment of the consistency of genotype effects.

We clarify that although within-environment replication was limited, genotypes were evaluated across multiple independent environments (distinct locations, seasons, and sowing dates) under standardized artificial inoculation protocols. This multi-environment framework allowed partitioning of genotype and genotype × environment interaction effects, thereby providing inferential support for the robustness of the phenotypic dataset used for GWAS. To further ensure robustness of the association results, GWAS was conducted using multiple complementary models implemented in GAPIT, incorporating principal components (fixed effects) and kinship matrices (random effects) to control for population structure and relatedness. Marker–trait associations were interpreted conservatively, emphasizing loci detected across multiple environments and/or supported by local LD structure. This multi-model, multi-environment consistency criterion reduces the likelihood of spurious associations and aligns with established practices in discovery-stage GWAS of complex traits.[8,9].

These clarifications, along with expanded variance component reporting and explicit discussion of design limitations, have now been incorporated into the revised manuscript.

3. Statistical analysis
- The statistical models used are missing
- Given the lack of replication, genomic repeatability can be estimated to gauge consistency of marker effects within environments, how relevant each environment can be concluded in such away!
- The models used for GWAS are mentioned, but the model specification (fixed vs random terms, kinship, structure correction) is missing. No validation or cross-model comparison is presented.
- A hierarchical clustering of the 14 environments based on the pairwise Pearson correlation coefficients for the disease is missing.

Author Response:

We thank the reviewer for the constructive comments on statistical analysis.

The revised manuscript now explicitly details the statistical models used for both phenotypic analysis and GWAS. Different statistical parameters were estimated using mixed linear models, and heritability on a genotype-mean basis is now reported in Table 2 of the revised manuscript.

For GWAS, we clarified that population structure (principal components) was included as fixed effects and kinship as a random effect within the GAPIT framework. Full model specifications are provided in Supplementary Document 1, with explicit reference in the main text.

Although within-environment replication was limited, robustness of marker effects was assessed through multi-environment evaluation and conservative interpretation of associations supported across multiple models and/or environments. This consistency-based approach serves as internal validation of the reported loci.

Additionally, hierarchical clustering of the 14 environments based on pairwise Pearson correlations of the WB index has now been conducted and included (Fig 3), with corresponding methodological details added.

4. Purpose and interpretation of environment-specific GWAS
- Running GWAS within each environment is acceptable, but the rationale is unclear; is it to capture race-specific resistance, breakdown effects, or local adaptation?
- The authors could have categorized loci as single-environment QTL (SEQ) versus multi-environment QTL (MEQ) to highlight consistency of associations.

Author Response:

We thank the reviewer for this thoughtful comment and clarify the rationale for conducting environment-specific GWAS.

Environment-specific GWAS was performed to capture genetic associations expressed under distinct epidemic scenarios, including variation in disease pressure, environmental conditions, and potential differences in pathogen composition across locations and planting times (there was natural inoculum in addition to the artificially applied inoculum). Rather than targeting race-specific resistance per se, this approach was intended to identify loci whose effects are detectable under particular disease-favorable conditions, as well as those that remain stable across contrasting environments. This strategy is commonly used in disease resistance GWAS to account for strong genotype × environment interactions and heterogeneity in disease expression.

To address consistency, robustness of associations was evaluated by comparing GWAS signals across environments and analytical models, and loci detected repeatedly were emphasized in the Results and Discussion. The type of QTLs like single or overlapped, their detections in different environments across model was included in the Table 3 of the revised manuscript.

We have clarified this rationale in the revised manuscript to better explain the purpose and interpretation of environment-specific GWAS analyses.

5. QTL definition and independence
- The criteria which you use for grouping SNP into QTL based on linkage disequilibirum need to be pointed. Especially for introgression they may not be optimal because it will depend on the frequency of a given introgression in a population whether MTAs will be grouped into a single QTL or several QTL.
- The manuscript lists several QTL but does not clarify how many are independent versus overlapping. Several QTL may be artifacts of LD clustering.
- The authors need to summarize the QTLs with the corresponding relevant information and then specify the leading marker of the region.
- The LD thresholds (r² ≥ xx, D′ ≥ xx) are arbitrary; the choice and its implications, especially in regions with introgressions, need justification. The delineation of the QTL is not clear enough, SNPs located on the same chromosome and within which Mb distance were considered as representatives of the same QTL when their LD measured as r2 was greater to what threshold? which threshold has been used for SNPs located on different chromosomes if significant SNPs are mismapped (I hope the authors omitted those missmaped ones).
- For introgressed segments, LD-based grouping may merge or split true QTL incorrectly depending on allele frequency and haplotype structure.

Author Response:

We thank the reviewer for these detailed and constructive comments regarding QTL definition and independence.

In this study, QTLs were defined by grouping significant marker–trait associations (MTAs) into QTL regions based on local linkage disequilibrium (LD) structure, rather than by individual SNPs alone. Chromosome-wise LD patterns (r² and D′) were examined and used to delineate QTL boundaries. This criteria was already explained in the Line 207-210. To improve transparency, we have updated Supplementary Table-2 listing all significant SNPs within each QTL region and number of overlapping QTLs within the identified QTL. This approach was adopted because QTLs are intended to represent LD-supported genomic regions that are informative for downstream candidate gene identification, rather than relying on single-marker signals.

We recognize that extended LD, particularly within large genomic regions, may lead to clusters of closely linked MTAs. Accordingly, multiple significant SNPs detected within a single LD block were considered to represent one QTL region, whereas distinct LD blocks separated by physical distance and reduced LD were treated as independent QTL regions when supported by the LD structure. This strategy avoids artificial inflation of QTL numbers due to LD clustering and is consistent with discovery-stage GWAS practice. The updated supplementary table 2 clarifies the distribution of MTAs within each QTL region and number of overlapping loci. To further improve clarity, QTLs are now summarized using consistent terminology, including chromosome, physical interval (bp) and number of supporting SNPs.

The LD thresholds applied (r² ≥ 0.70 and/or D′ ≥ 0.85) were selected based on empirical chromosome-wise LD decay patterns observed in this population (Figure S3) and are consistent with thresholds commonly applied in wheat GWAS. Previous studies have used a broad range of LD cutoffs (e.g., r² ≥ 0.20 to r² ≥ 0.90), depending on marker density and population structure [10,11]. While r² ≥ 0.80 is often used to define tag SNPs, an r² ≥ 0.70 threshold is frequently adopted in high-density panels to balance LD resolution, marker redundancy, and computational efficiency, particularly in regions with extended LD such as introgressed segments.

6. Missing PVE and dominance information
- The proportion of variance explained (PVE) by each QTL is not described. It is unclear how environmental data were incorporated into PVE and degree-of-dominance calculations.
- Substantial differences between environments suggest that per-environment effect sizes should have been visualized (e.g. heatmap of overlapping SNPs).

Author Response:

We thank the reviewer for this comment. In the present study, GWAS was conducted using large-scale disease response data derived from non-replicated trials across multiple environments. Under such conditions, reliable estimation of phenotypic variance components is not statistically defensible, and therefore conventional calculation of phenotypic variance explained (PVE) was not attempted. This decision is consistent with established practice in GWAS of disease resistance using unreplicated field nurseries, where variance-based metrics may be biased or misleading[12].

Instead, we emphasize environment-wise additive effect estimates, cross-environment consistency, and cross-model robustness. GWAS was performed separately for each environment using six models implemented in GAPIT, and loci were prioritized based on reproducibility of associations and consistency of effect direction rather than variance explained. Dominance and degree-of-dominance effects were not estimated, as analyses were conducted under an additive genetic framework, which is standard for GWAS in unreplicated disease screening panels and is most relevant for downstream marker-assisted and genomic selection[13]. To address environmental heterogeneity, we have added the SNP list in Table 3 facilitating comparison of stable and environment-dependent loci without relying on variance-based measures.

Bibliography:

1.	He X, Gupta V, Bainsla NK, Chawade A, Singh PK. Breeding for wheat blast resistance. Wheat Blast. 2020; 163–174.

2.	Juliana P, He X, Kabir MR, Roy KK, Anwar MB, Marza F, et al. Genome-wide association mapping for wheat blast resistance in CIMMYT’s international screening nurseries evaluated in Bolivia and Bangladesh. Sci Rep. 2020;10: 15972.

3.	Fady B, Médail F. TEMPERATE AND MEDITERRANEAN FORESTS | Mediterranean Forest Ecosystems. In: Burley JBT-E of FS, editor. Oxford: Elsevier; 2004. pp. 1403–1414. doi:https://doi.org/10.1016/B0-12-145160-7/00177-0

4.	He X, Juliana P, Kabir MR, Roy KK, Islam R, Marza F, et al. Screening and mapping for head blast resistance in a panel of CIMMYT and South Asian bread wheat germplasm. Front Genet. 2021;12: 679162.

5.	Cruppe G, Silva P, Lemes da Silva C, Peterson G, Pedley KF, Cruz CD, et al. Genome‐wide association reveals limited benefits of pyramiding the 1B and 1D loci with the 2NvS translocation for wheat blast control. Crop Sci. 2021;61: 1089–1103.

6.	Wu L, He X, Kabir MR, Roy KK, Anwar MB, Marza F, et al. Genetic sources and loci for wheat head blast resistance identified by genome-wide association analysis. Crop J. 2022;10: 793–801.

7.	Cruz CD, Peterson GL, Bockus WW, Kankanala P, Dubcovsky J, Jordan KW, et al. The 2NS translocation from Aegilops ventricosa confers resistance to the Triticum pathotype of Magnaporthe oryzae. Crop Sci. 2016;56: 990–1000. doi:10.2135/cropsci2015.07.0410

8.	Xu Y, Li P, Zou C, Lu Y, Xie C, Zhang X, et al. Enhancing genetic gain in the era of molecular breeding. J Exp Bot. 2017;68: 2641–2666. doi:10.1093/jxb/erx135

9.	Huang X, Han B. Natural Variations and Genome-Wide Association Studies in Crop Plants. Annu Rev Plant Biol. 2014;65: 531–551. doi:https://doi.org/10.1146/annurev-arplant-050213-035715

10.	Neumann K, Kobiljski B, Denc S, Varshney RK, Bo A. Genome-wide association mapping : a case study in bread wheat ( Triticum aestivum L .). 2011; 37–58. doi:10.1007/s11032-010-9411-7

11.	Davis UC, Godoy J, Gizaw S, Chao S, Blake N, Carter A, et al. Genome-wide Association Study of Agronomic Traits in a Spring-Planted North American Elite Hard Red Spring Wheat Panel. 2018. doi:10.2135/cropsci2017.07.0423

12.	Juliana P, Poland J, Huerta-Espino J, Shrestha S, Crossa J, Crespo-Herrera L, et al. Improving grain yield, stress resilience and quality of bread wheat using large-scale genomics. Nat Genet. 2019;51: 1530–1539. doi:10.1038/s41588-019-0496-6

13.	Lipka AE, Tian F, Wang Q, Peiffer J, Li M, Bradbury PJ, et al. GAPIT: Genome association and prediction integrated tool. Bioinformatics. 2012;28: 2397–2399. doi:10.1093/bioinformatics/bts444
