# Supplementary material for: Field screening and genetic mapping of wheat blast resistance for a panel of common wheat from Bangladesh
Source: PLoS One. 2026 Jun 11;21(6):e0349201. doi: 10.1371/journal.pone.0349201 (PMC13258015; doi:10.1371/journal.pone.0349201)
Supplement: Peer review history

# Peer review: Decision Letter 0

**DOI:** 10.1371/journal.pone.0349201.r001

**Authors:** Wuletaw Tadesse

7 Nov 2025

PONE-D-25-52203

Field screening and genetic mapping of wheat blast resistance for a panel of common wheat from Bangladesh

PLOS ONE

Dear Dr. Singh,

Thank you for submitting your manuscript to PLOS ONE. After careful consideration, we feel that it has merit but does not fully meet PLOS ONE’s publication criteria as it currently stands. Therefore, we invite you to submit a revised version of the manuscript that addresses the points raised during the review process.

Please submit your revised manuscript by Dec 22 2025 11:59PM. If you will need more time than this to complete your revisions, please reply to this message or contact the journal office at plosone@plos.org. When you're ready to submit your revision, log on to https://www.editorialmanager.com/pone/ and select the 'Submissions Needing Revision' folder to locate your manuscript file.

Please include the following items when submitting your revised manuscript:

- A rebuttal letter that responds to each point raised by the academic editor and reviewer(s). You should upload this letter as a separate file labeled 'Response to Reviewers'.
- A marked-up copy of your manuscript that highlights changes made to the original version. You should upload this as a separate file labeled 'Revised Manuscript with Track Changes'.
- An unmarked version of your revised paper without tracked changes. You should upload this as a separate file labeled 'Manuscript'.

If you would like to make changes to your financial disclosure, please include your updated statement in your cover letter. Guidelines for resubmitting your figure files are available below the reviewer comments at the end of this letter.

If applicable, we recommend that you deposit your laboratory protocols in protocols.io to enhance the reproducibility of your results. Protocols.io assigns your protocol its own identifier (DOI) so that it can be cited independently in the future. For instructions see: https://journals.plos.org/plosone/s/submission-guidelines#loc-laboratory-protocols. Additionally, PLOS ONE offers an option for publishing peer-reviewed Lab Protocol articles, which describe protocols hosted on protocols.io. Read more information on sharing protocols at https://plos.org/protocols?utm_medium=editorial-email&utm_source=authorletters&utm_campaign=protocols.

We look forward to receiving your revised manuscript.

Kind regards,

Wuletaw Tadesse

Academic Editor

PLOS ONE

Journal Requirements:

When submitting your revision, we need you to address these additional requirements.

1. Please ensure that your manuscript meets PLOS ONE's style requirements, including those for file naming. The PLOS ONE style templates can be found at

https://journals.plos.org/plosone/s/file?id=wjVg/PLOSOne_formatting_sample_main_body.pdf and

https://journals.plos.org/plosone/s/file?id=ba62/PLOSOne_formatting_sample_title_authors_affiliations.pdf.

2. Thank you for stating the following financial disclosure:

“Accelerating Genetic Gain (AGG) in Maize and Wheat Project Grant INV-003439 funded by the Bill and Melinda Gates Foundation (BMGF), the Foreign and Commonwealth Development Office (FCDO)”

Please state what role the funders took in the study.  If the funders had no role, please state: "The funders had no role in study design, data collection and analysis, decision to publish, or preparation of the manuscript."

If this statement is not correct you must amend it as needed.

Please include this amended Role of Funder statement in your cover letter; we will change the online submission form on your behalf.

3. In the online submission form you indicate that your data is not available for proprietary reasons and have provided a contact point for accessing this data. Please note that your current contact point is a co-author on this manuscript. According to our Data Policy, the contact point must not be an author on the manuscript and must be an institutional contact, ideally not an individual. Please revise your data statement to a non-author institutional point of contact, such as a data access or ethics committee, and send this to us via return email. Please also include contact information for the third party organization, and please include the full citation of where the data can be found.

4. Thank you for stating the following in the Acknowledgments Section of your manuscript:

“Financial supports from the Australian Centre for International Agricultural Research, Australia, One CGIAR Initiatives ABI and PHI, the Ministry of Agriculture, Forestry and Fisheries of Japan, The Krishi Gobeshona Foundation-Bangladesh, and Accelerating Genetic Gain (AGG) in Maize and Wheat Project Grant INV-003439 funded by the Bill and Melinda Gates Foundation (BMGF), the Foreign and Commonwealth Development Office (FCDO) are gratefully acknowledged. “

We note that you have provided additional information within the Acknowledgements Section that is not currently declared in your Funding Statement. Please note that funding information should not appear in the Acknowledgments section or other areas of your manuscript. We will only publish funding information present in the Funding Statement section of the online submission form.

Please remove any funding-related text from the manuscript and let us know how you would like to update your Funding Statement. Currently, your Funding Statement reads as follows:

“Accelerating Genetic Gain (AGG) in Maize and Wheat Project Grant INV-003439 funded by the Bill and Melinda Gates Foundation (BMGF), the Foreign and Commonwealth Development Office (FCDO)”

Please include your amended statements within your cover letter; we will change the online submission form on your behalf.

5. If the reviewer comments include a recommendation to cite specific previously published works, please review and evaluate these publications to determine whether they are relevant and should be cited. There is no requirement to cite these works unless the editor has indicated otherwise.

Additional Editor Comments (if provided):

Dear Authors,

A major revision is recommended from reviewers. Please revise your manuscript carefully and submit accordingly. Attached is the comments of the 3 reviewers.

[Note: HTML markup is below. Please do not edit.]

Reviewers' comments:

Reviewer's Responses to Questions

**Comments to the Author**

1. Is the manuscript technically sound, and do the data support the conclusions?

The manuscript must describe a technically sound piece of scientific research with data that supports the conclusions. Experiments must have been conducted rigorously, with appropriate controls, replication, and sample sizes. The conclusions must be drawn appropriately based on the data presented.

Reviewer #1: Yes

Reviewer #2: Partly

Reviewer #3: Partly

**********

2. Has the statistical analysis been performed appropriately and rigorously?

Reviewer #1: Yes

Reviewer #2: Yes

Reviewer #3: No

**********

3. Have the authors made all data underlying the findings in their manuscript fully available?

The PLOS Data policy requires authors to make all data underlying the findings described in their manuscript fully available without restriction, with rare exception (please refer to the Data Availability Statement in the manuscript PDF file). The data should be provided as part of the manuscript or its supporting information, or deposited to a public repository. For example, in addition to summary statistics, the data points behind means, medians and variance measures should be available. If there are restrictions on publicly sharing data—e.g. participant privacy or use of data from a third party—those must be specified.

Reviewer #1: Yes

Reviewer #2: Yes

Reviewer #3: No

**********

4. Is the manuscript presented in an intelligible fashion and written in standard English?

PLOS ONE does not copyedit accepted manuscripts, so the language in submitted articles must be clear, correct, and unambiguous. Any typographical or grammatical errors should be corrected at revision, so please note any specific errors here.

Reviewer #1: Yes

Reviewer #2: Yes

Reviewer #3: No

**********

5. Review Comments to the Author

Please use the space provided to explain your answers to the questions above. You may also include additional comments for the author, including concerns about dual publication, research ethics, or publication ethics. (Please upload your review as an attachment if it exceeds 20,000 characters)

Reviewer #1: This manuscript presents a field-based evaluation and genome-wide association study (GWAS) for wheat blast resistance using a diverse panel of 432 common wheat accessions genotyped through ddRAD-seq. The topic is highly relevant and timely for wheat breeding programs, particularly in Bangladesh and other blast-endemic regions. The integration of field phenotyping and genotyping provides valuable insights into the genetic architecture of resistance, including confirmation of the well-known 2NS/2AS translocation and identification of additional loci across the genome.

While the experimental design is sound and the results are promising, the manuscript would benefit from clarification and improvement in several key areas.

Introduction

The introduction would be coherent and more engaging if it began with a general overview of wheat production and its importance in Bangladesh, followed by a concise description of wheat blast disease, its impact, and its specific relevance to the region. This could then transition into a discussion of resistance mechanisms, including the 2NS/2AS translocation, known resistance genes such as the Rmg loci, and previously identified QTLs.

It is recommended to include a brief summary of previously reported QTLs and SNPs associated with wheat blast resistance identified through GWAS or QTL mapping.

The objectives section would benefit from a clearer statement emphasizing the novelty and originality of the current study, and how it differs from and advances beyond previous investigations.

Materials and Methods

In the description “1 m double rows separated by 20 cm spacing in all three locations, and no replication was arranged within each sowing,” please specify the exact experimental design and briefly justify the statistical rationale for its use.

The use of the WB index (incidence × severity) as the main phenotypic trait is appropriate and common in wheat blast studies. However, since the WB index may conflate genetic resistance with phenological escape, it would be helpful to explain why complementary traits, such as days to heading, spike sterility, and yield, were not recorded.

Please confirm whether a standardized wheat blast scoring protocol was used, for example that described by Cruz et al. (2016):

Cruz, C. D., W. W. Bockus, J. P. Stack, B. Valent, J. N. Maciel, and G. L. Peterson (2016). A Standardized Inoculation Protocol to Test Wheat Cultivars for Reaction to Head Blast Caused by Magnaporthe oryzae (Triticum pathotype). Plant Health Progress, 17(3), 186–187. https://doi.org/10.1094/PHP-BR-16-0041

The authors are encouraged to justify the choice of sampling (10 spikes per plot at 21 days) and add references to standard methods.

The results section currently lacks estimates of trait heritability and genotype × environment (G×E) variance components. It is recommended to fit a multi-environment mixed model (e.g., including genotype, environment, and G×E as random effects) and to report broad-sense heritability (H²) within and across environments.

The statement that Manhattan plots were generated using “the pooled highest WB scores across 14 environments” requires clarification. Please specify whether GWAS was performed using BLUEs, BLUPs, or another aggregated phenotype.

Results

Lines 219–221: The site “Jash20a” is described as both a “low-pressure” and a “highly conducive” environment. Please verify and correct this discripancy.

Line 299: Please indicate the exact numerical threshold used after Bonferroni correction.

Line 311: Correct the phrase “form GWAS” to “from GWAS”.

In addition to Supplementary Figure 3, consider including a genome-wide LD-decay curve (average r² vs. physical distance) and report the LD-decay threshold, as this would add useful genomic context.

For each reported lead SNP/QTL, please provide estimates of allelic effect size and the percentage of phenotypic variance explained (R²), as these values are essential for breeders to assess practical significance.

Discussion

Line 349: Please justify the choice of the ddRAD-seq approach, particularly given that after filtering, only 7,211 SNPs remained from an initial 82,948.

Discuss how the lower SNP density may limit fine-scale mapping resolution.

Line 359: The discussion would benefit from a more explicit comparison between the QTLs identified in this study and those reported in previous GWAS and mapping studies on wheat blast resistance (e.g., Cruppe et al., 2023; Phuke et al., 2022; Goddard et al., 2020; He et al., 2021; Juliana et al., 2020). At present, it is not entirely clear which of the Qwb.cim loci correspond to previously known regions, such as the 2NS/2AS translocation, and which may represent novel genomic regions.

Reviewer #2: Dear Editor,

The authors did field screening and genetic mapping of wheat blast using six different model analysis. The association analysis identified about 1,121 significant markers and grouped 45 QTLS. The study is important for the scientific community.

I have some queries and suggestions kindly address that

1. The experiment screened 432 genotypes for wheat blast in seven environment’s using two row per genotype and it is not replicated (line # 118). The within-environment error variance could not be estimated, because experimental units were not replicated. Differences between genotypes may be confounded with micro environment variation. The GWAS results based on non-replicated are not reliable enough. The authors should give clear evidence how to resolve such issues.

2. Candidate genes need to be explored in different database.

Reviewer #3: The study addresses an important topic which is Genetic dissection of wheat blast resistance in Bangladeshi germplasm; but falls short in methodological rigor, interpretation of GWAS results, and clarity regarding QTL definition and independence. The experimental design and statistical analyses do not support the strength of the claims made.

Major Concerns

1. Material description and population structure

- The mention of “respective parental lines” is ambiguous. It is unclear whether these include hybrid materials or simply refer to the parental lines used to generate the advanced breeding lines and varieties included in the panel. The authors should clarify whether all or only a subset of parental lines were represented. Furthermore, the later reference to “synthetic lines” is inconsistent with the materials description, as no synthetic germplasm was mentioned in the Plant Materials section. This discrepancy should be addressed and the composition of the panel clearly defined.

- The structure plot shows weak distinction among groups; authors claim clear separation, which is not evident.

- The PCA cloud contradicts the claim of “distinct clusters,” and the description of synthetic wheat should be supported by pedigree data.

2. Experimental Design

- The authors treated different planting dates as replications, and the presentation of the ANOVA results (Table 1) is insufficient to support any quantitative inference about genetic variance or heritability. Moreover, the manuscript does not include any variance partitioning or heritability estimates across environments, which are essential for evaluating the consistency of genotype effects and the robustness of the GWAS results. Without within- and across-environment variance component analysis, the statistical strength of the reported associations remains uncertain.

3. Statistical analysis

- The statistical models used are missing

- Given the lack of replication, genomic repeatability can be estimated to gauge consistency of marker effects within environments, how relevant each environment can be concluded in such away!

- The models used for GWAS are mentioned, but the model specification (fixed vs random terms, kinship, structure correction) is missing. No validation or cross-model comparison is presented.

- A hierarchical clustering of the 14 environments based on the pairwise Pearson correlation coefficients for the disease is missing.

4. Purpose and interpretation of environment-specific GWAS

- Running GWAS within each environment is acceptable, but the rationale is unclear; is it to capture race-specific resistance, breakdown effects, or local adaptation?

- The authors could have categorized loci as single-environment QTL (SEQ) versus multi-environment QTL (MEQ) to highlight consistency of associations.

5. QTL definition and independence

- The criteria which you use for grouping SNP into QTL based on linkage disequilibirum need to be pointed. Especially for introgression they may not be optimal because it will depend on the frequency of a given introgression in a population whether MTAs will be grouped into a single QTL or several QTL.

- The manuscript lists several QTL but does not clarify how many are independent versus overlapping. Several QTL may be artifacts of LD clustering.

- The authors need to summarize the QTLs with the corresponding relevant information and then specify the leading marker of the region.

- The LD thresholds (r² ≥ xx, D′ ≥ xx) are arbitrary; the choice and its implications, especially in regions with introgressions, need justification. The delineation of the QTL is not clear enough, SNPs located on the same chromosome and within which Mb distance were considered as representatives of the same QTL when their LD measured as r2 was greater to what threshold? which threshold has been used for SNPs located on different chromosomes if significant SNPs are mismapped (I hope the authors omitted those missmaped ones).

- For introgressed segments, LD-based grouping may merge or split true QTL incorrectly depending on allele frequency and haplotype structure.

6. Missing PVE and dominance information

- The proportion of variance explained (PVE) by each QTL is not described. It is unclear how environmental data were incorporated into PVE and degree-of-dominance calculations.

- Substantial differences between environments suggest that per-environment effect sizes should have been visualized (e.g. heatmap of overlapping SNPs).

**********

6. PLOS authors have the option to publish the peer review history of their article (what does this mean?). If published, this will include your full peer review and any attached files.

If you choose “no”, your identity will remain anonymous but your review may still be made public.

**Do you want your identity to be public for this peer review?** For information about this choice, including consent withdrawal, please see our Privacy Policy.

Reviewer #1: No

Reviewer #2: **Yes:** Molla Mekonnen Kassie

Reviewer #3: No

**********

[NOTE: If reviewer comments were submitted as an attachment file, they will be attached to this email and accessible via the submission site. Please log into your account, locate the manuscript record, and check for the action link "View Attachments". If this link does not appear, there are no attachment files.]

To ensure your figures meet our technical requirements, please review our figure guidelines: https://journals.plos.org/plosone/s/figures

You may also use PLOS’s free figure tool, NAAS, to help you prepare publication quality figures: https://journals.plos.org/plosone/s/figures#loc-tools-for-figure-preparation.

NAAS will assess whether your figures meet our technical requirements by comparing each figure against our figure specifications.

Attachment Submitted filename: PONE-D-25-52203.pdf

Attachment Submitted filename: Review_PLOSone_SEL.docx

# Author comment: Author response to Decision Letter 1

**DOI:** 10.1371/journal.pone.0349201.r002

6 Apr 2026

Thank you for submitting your manuscript to PLOS ONE. After careful consideration, we feel that it has merit but does not fully meet PLOS ONE’s publication criteria as it currently stands. Therefore, we invite you to submit a revised version of the manuscript that addresses the points raised during the review process.

Please submit your revised manuscript by Dec 22 2025 11:59PM. If you will need more time than this to complete your revisions, please reply to this message or contact the journal office at plosone@plos.org. When you're ready to submit your revision, log on to https://www.editorialmanager.com/pone/ and select the 'Submissions Needing Revision' folder to locate your manuscript file.

Please include the following items when submitting your revised manuscript:

A rebuttal letter that responds to each point raised by the academic editor and reviewer(s). You should upload this letter as a separate file labeled 'Response to Reviewers'.

A marked-up copy of your manuscript that highlights changes made to the original version. You should upload this as a separate file labeled 'Revised Manuscript with Track Changes'.

An unmarked version of your revised paper without tracked changes. You should upload this as a separate file labeled 'Manuscript'.

If you would like to make changes to your financial disclosure, please include your updated statement in your cover letter. Guidelines for resubmitting your figure files are available below the reviewer comments at the end of this letter.

If applicable, we recommend that you deposit your laboratory protocols in protocols.io to enhance the reproducibility of your results. Protocols.io assigns your protocol its own identifier (DOI) so that it can be cited independently in the future. For instructions see: https://journals.plos.org/plosone/s/submission-guidelines#loc-laboratory-protocols. Additionally, PLOS ONE offers an option for publishing peer-reviewed Lab Protocol articles, which describe protocols hosted on protocols.io. Read more information on sharing protocols at https://plos.org/protocols?utm_medium=editorial-email&utm_source=authorletters&utm_campaign=protocols.

Author Response:

Thank you for offering us to publish protocol. We do not have novel laboratory protocol to publish for this manuscript.

We look forward to receiving your revised manuscript.

Kind regards,

Wuletaw Tadesse

Academic Editor

PLOS ONE

Journal Requirements:

When submitting your revision, we need you to address these additional requirements.

1. Please ensure that your manuscript meets PLOS ONE's style requirements, including those for file naming. The PLOS ONE style templates can be found at

https://journals.plos.org/plosone/s/file?id=wjVg/PLOSOne_formatting_sample_main_body.pdf and

https://journals.plos.org/plosone/s/file?id=ba62/PLOSOne_formatting_sample_title_authors_affiliations.pdf.

Author Response:

The revised manuscript has reorganised as per the PLOS ONE’s style requirement.

2. Thank you for stating the following financial disclosure:

“Accelerating Genetic Gain (AGG) in Maize and Wheat Project Grant INV-003439 funded by the Bill and Melinda Gates Foundation (BMGF), the Foreign and Commonwealth Development Office (FCDO)”

Please state what role the funders took in the study. If the funders had no role, please state: "The funders had no role in study design, data collection and analysis, decision to publish, or preparation of the manuscript."

If this statement is not correct you must amend it as needed.

Please include this amended Role of Funder statement in your cover letter; we will change the online submission form on your behalf.

3. In the online submission form you indicate that your data is not available for proprietary reasons and have provided a contact point for accessing this data. Please note that your current contact point is a co-author on this manuscript. According to our Data Policy, the contact point must not be an author on the manuscript and must be an institutional contact, ideally not an individual. Please revise your data statement to a non-author institutional point of contact, such as a data access or ethics committee, and send this to us via return email. Please also include contact information for the third party organization, and please include the full citation of where the data can be found.

4. Thank you for stating the following in the Acknowledgments Section of your manuscript:

“Financial supports from the Australian Centre for International Agricultural Research, Australia, One CGIAR Initiatives ABI and PHI, the Ministry of Agriculture, Forestry and Fisheries of Japan, The Krishi Gobeshona Foundation-Bangladesh, and Accelerating Genetic Gain (AGG) in Maize and Wheat Project Grant INV-003439 funded by the Bill and Melinda Gates Foundation (BMGF), the Foreign and Commonwealth Development Office (FCDO) are gratefully acknowledged. “

We note that you have provided additional information within the Acknowledgements Section that is not currently declared in your Funding Statement. Please note that funding information should not appear in the Acknowledgments section or other areas of your manuscript. We will only publish funding information present in the Funding Statement section of the online submission form.

Please remove any funding-related text from the manuscript and let us know how you would like to update your Funding Statement. Currently, your Funding Statement reads as follows:

“Accelerating Genetic Gain (AGG) in Maize and Wheat Project Grant INV-003439 funded by the Bill and Melinda Gates Foundation (BMGF), the Foreign and Commonwealth Development Office (FCDO)”

Please include your amended statements within your cover letter; we will change the online submission form on your behalf.

Author Response:

We removed funding information from Acknowledgement section, and we , and we have updated the Acknowledgement section in the revised manuscript.

5. If the reviewer comments include a recommendation to cite specific previously published works, please review and evaluate these publications to determine whether they are relevant and should be cited. There is no requirement to cite these works unless the editor has indicated otherwise.

Additional Editor Comments (if provided):

Dear Authors,

A major revision is recommended from reviewers. Please revise your manuscript carefully and submit accordingly. Attached is the comments of the 3 reviewers.

Reviewers' comments:

Reviewer #1: This manuscript presents a field-based evaluation and genome-wide association study (GWAS) for wheat blast resistance using a diverse panel of 432 common wheat accessions genotyped through ddRAD-seq. The topic is highly relevant and timely for wheat breeding programs, particularly in Bangladesh and other blast-endemic regions. The integration of field phenotyping and genotyping provides valuable insights into the genetic architecture of resistance, including confirmation of the well-known 2NS/2AS translocation and identification of additional loci across the genome.

While the experimental design is sound and the results are promising, the manuscript would benefit from clarification and improvement in several key areas.

Introduction

The introduction would be coherent and more engaging if it began with a general overview of wheat production and its importance in Bangladesh, followed by a concise description of wheat blast disease, its impact, and its specific relevance to the region. This could then transition into a discussion of resistance mechanisms, including the 2NS/2AS translocation, known resistance genes such as the Rmg loci, and previously identified QTLs.

It is recommended to include a brief summary of previously reported QTLs and SNPs associated with wheat blast resistance identified through GWAS or QTL mapping.

The objectives section would benefit from a clearer statement emphasizing the novelty and originality of the current study, and how it differs from and advances beyond previous investigations.

Author Response:

We thank the reviewer for the thoughtful recommendations to improve the structure and clarity of the Introduction.

In the revised manuscript, the Introduction has been substantially reorganized to enhance coherence and narrative flow. It now begins with the importance of wheat production and food security in Bangladesh, followed by a concise overview of wheat blast, its rapid global spread, and its specific agronomic and economic relevance to the region. The discussion then transitions to resistance mechanisms, including the 2NS/2AS translocation, major seedling resistance genes (e.g., Rmg loci), and previously identified QTL from both bi-parental and GWAS studies.

To address the reviewer’s recommendation, we have incorporated a structured summary of previously reported QTL and significant SNP associations for wheat blast resistance. This synthesis highlights the predominant role of the 2NS segment across studies, as well as the generally minor and environment-dependent effects of non-2NS loci reported in South American and South Asian germplasm.

The objectives section has been revised to more clearly articulate the originality and advancement of the present study. Unlike many prior investigations that focused primarily on CIMMYT-derived or South American germplasm, or relied heavily on controlled-condition screening, our study evaluates a diverse panel of Bangladeshi wheat genotypes under multi-environment field conditions representing natural epidemic variability. Furthermore, the use of a multi-model GWAS framework combined with LD-based locus delineation and cross-environment consistency criteria provides a more comprehensive assessment of resistance architecture relevant to local breeding programs.

These revisions clarify how the present study builds upon and extends prior South Asian GWAS efforts by focusing on locally adapted germplasm, environment-specific resistance dynamics, and identification of candidate loci beyond the widely deployed 2NS source.

Materials and Methods

In the description “1 m double rows separated by 20 cm spacing in all three locations, and no replication was arranged within each sowing,” please specify the exact experimental design and briefly justify the statistical rationale for its use.

The use of the WB index (incidence × severity) as the main phenotypic trait is appropriate and common in wheat blast studies. However, since the WB index may conflate genetic resistance with phenological escape, it would be helpful to explain why complementary traits, such as days to heading, spike sterility, and yield, were not recorded.

Please confirm whether a standardized wheat blast scoring protocol was used, for example that described by Cruz et al. (2016): Cruz, C. D., W. W. Bockus, J. P. Stack, B. Valent, J. N. Maciel, and G. L. Peterson (2016). A Standardized Inoculation Protocol to Test Wheat Cultivars for Reaction to Head Blast Caused by Magnaporthe oryzae (Triticum pathotype). Plant Health Progress, 17(3), 186–187. https://doi.org/10.1094/PHP-BR-16-0041.

The authors are encouraged to justify the choice of sampling (10 spikes per plot at 21 days) and add references to standard methods.

The results section currently lacks estimates of trait heritability and genotype × environment (G×E) variance components. It is recommended to fit a multi-environment mixed model (e.g., including genotype, environment, and G×E as random effects) and to report broad-sense heritability (H²) within and across environments.

The statement that Manhattan plots were generated using “the pooled highest WB scores across 14 environments” requires clarification. Please specify whether GWAS was performed using BLUEs, BLUPs, or another aggregated phenotype.

Author Response:

We thank the reviewer for these helpful suggestions and provide the following clarifications.

The field trials were conducted using an unreplicated design, with different sowing dates treated as temporal replications within each environment. This approach has been commonly used in large-scale wheat blast screening nurseries under artificial inoculation, where logistical constraints limit full replication while allowing evaluation of a large number of genotypes [1,2].

The wheat blast (WB) index (incidence × severity) was used as the primary phenotypic trait because it is a widely accepted metric for quantifying head blast under field epidemic conditions[2–4]. We acknowledge the potential confounding effect of phenological escape. To address this, days to heading and plant height were recorded for all genotypes and are now reported in the revised manuscript to support interpretation of disease response independent of maturity differences. Artificial inoculation targeting to each genotype’s anthesis and mist irrigation were implemented to minimize escape effects due to asynchronous flowering.

A standardized inoculation and scoring protocol consistent with Cruz et al. (2016) and subsequent CIMMYT-led studies was followed. Disease assessment based on 10 tagged spikes per plot at 21 days after anthesis has been widely applied and shown to provide stable estimates of wheat blast severity under field conditions and the reference were added in the revised manuscript.

In response to the reviewer’s recommendation, variance components were estimated using mixed linear models, and broad-sense heritability on a genotype-mean basis (H_gm^2) is now reported (Table 1). Genotype × environment interaction was evaluated through multi-environment analysis, including within-environment ANOVA and likelihood ratio testing implemented in the Metan R package. These additions strengthen quantitative inference regarding the consistency of genotype performance [5,6].

Regarding the GWAS phenotype, analyses were conducted using the maximum WB index per genotype across the 14 environments to capture resistance performance under severe epidemic conditions. The previous wording has been clarified in the revised manuscript. While BLUEs or BLUPs are commonly used in multi-environment analyses, the use of maximum disease expression was considered appropriate given the highly variable and epidemic-driven nature of wheat blast pressure, and aligns with the study objective of identifying loci effective under intense and variable disease conditions.

All relevant sections of the Materials and Methods and Results have been revised accordingly to improve transparency and statistical rigor.

Results

Lines 219–221: The site “Jash20a” is described as both a “low-pressure” and a “highly conducive” environment. Please verify and correct this discripancy.

Line 299: Please indicate the exact numerical threshold used after Bonferroni correction.

Line 311: Correct the phrase “form GWAS” to “from GWAS”.

Author Response:

We thank the reviewer for these helpful comments on result section. We have address them as follows:

The discrepancy in Lines 219–221 has been corrected. The reference to a highly conducive environment was a typographical error; the environment with high disease pressure is Jash21a, not Jash20a.

The exact Bonferroni-corrected threshold has now been clearly stated in the Results section as α = 0.05/7,211 SNPs (p = 6.93 × 10⁻⁶; −log₁₀(p) = 5.16).

The typographical error in Line 311 has been corrected from “form GWAS” to “from GWAS.”

In addition to Supplementary Figure 3, consider including a genome-wide LD-decay curve (average r² vs. physical distance) and report the LD-decay threshold, as this would add useful genomic context.

Author Response:

We thank the reviewer for this valuable suggestion. We have now included a genome-wide LD-decay curve (average r² vs. physical distance) as Figure S4. Background LD was estimated using the 95th percentile of inter-chromosomal marker r² values. Genome-wide LD decayed below this threshold at approximately 144 Kb, indicating relatively rapid LD decay and providing genomic context for QTL interval definition. The Methods and Results sections have been updated accordingly.

For each reported lead SNP/QTL, please provide estimates of allelic effect size and the percentage of phenotypic variance explained (R²), as these values are essential for breeders to assess practical significance.

Author Response:

We thank the reviewer for this important suggestion. In

Attachment Submitted filename: Response to Reviewers.docx

# Peer review: Decision Letter 1

**DOI:** 10.1371/journal.pone.0349201.r003

**Authors:** Wuletaw Tadesse

27 Apr 2026

Field screening and genetic mapping of wheat blast resistance for a panel of common wheat from Bangladesh

PONE-D-25-52203R1

Dear Dr. Singh

We’re pleased to inform you that your manuscript has been judged scientifically suitable for publication and will be formally accepted for publication once it meets all outstanding technical requirements.

Within one week, you’ll receive an e-mail detailing the required amendments. When these have been addressed, you’ll receive a formal acceptance letter and your manuscript will be scheduled for publication.

An invoice will be generated when your article is formally accepted. Please note, if your institution has a publishing partnership with PLOS and your article meets the relevant criteria, all or part of your publication costs will be covered. Please make sure your user information is up-to-date by logging into Editorial Manager at Editorial Manager® and clicking the ‘Update My Information' link at the top of the page. For questions related to billing, please contact billing support.

If your institution or institutions have a press office, please notify them about your upcoming paper to help maximize its impact. If they’ll be preparing press materials, please inform our press team as soon as possible -- no later than 48 hours after receiving the formal acceptance. Your manuscript will remain under strict press embargo until 2 pm Eastern Time on the date of publication. For more information, please contact onepress@plos.org.

Kind regards,

Wuletaw Tadesse

Academic Editor

PLOS One

Additional Editor Comments (optional):

no further comments

Reviewers' comments:

Reviewer's Responses to Questions

**Comments to the Author**

1. If the authors have adequately addressed your comments raised in a previous round of review and you feel that this manuscript is now acceptable for publication, you may indicate that here to bypass the “Comments to the Author” section, enter your conflict of interest statement in the “Confidential to Editor” section, and submit your "Accept" recommendation.

Reviewer #1: All comments have been addressed

Reviewer #2: All comments have been addressed

Reviewer #3: All comments have been addressed

**********

2. Is the manuscript technically sound, and do the data support the conclusions?

The manuscript must describe a technically sound piece of scientific research with data that supports the conclusions. Experiments must have been conducted rigorously, with appropriate controls, replication, and sample sizes. The conclusions must be drawn appropriately based on the data presented.

Reviewer #1: Yes

Reviewer #2: Yes

Reviewer #3: No

**********

3. Has the statistical analysis been performed appropriately and rigorously?

Reviewer #1: Yes

Reviewer #2: Yes

Reviewer #3: Yes

**********

4. Have the authors made all data underlying the findings in their manuscript fully available?

The PLOS Data policy requires authors to make all data underlying the findings described in their manuscript fully available without restriction, with rare exception (please refer to the Data Availability Statement in the manuscript PDF file). The data should be provided as part of the manuscript or its supporting information, or deposited to a public repository. For example, in addition to summary statistics, the data points behind means, medians and variance measures should be available. If there are restrictions on publicly sharing data—e.g. participant privacy or use of data from a third party—those must be specified.

Reviewer #1: Yes

Reviewer #2: Yes

Reviewer #3: Yes

**********

5. Is the manuscript presented in an intelligible fashion and written in standard English?

PLOS ONE does not copyedit accepted manuscripts, so the language in submitted articles must be clear, correct, and unambiguous. Any typographical or grammatical errors should be corrected at revision, so please note any specific errors here.

Reviewer #1: Yes

Reviewer #2: Yes

Reviewer #3: Yes

**********

6. Review Comments to the Author

Please use the space provided to explain your answers to the questions above. You may also include additional comments for the author, including concerns about dual publication, research ethics, or publication ethics. (Please upload your review as an attachment if it exceeds 20,000 characters)

Reviewer #1: The revised manuscript presents a genome-wide association study of wheat blast resistance using a diverse panel of Bangladeshi germplasm evaluated across multiple field environments. The study addresses an important topic and effectively integrates field phenotyping with genotyping data.

The authors have made substantial improvements in response to the reviewer comments, particularly in restructuring the Introduction, clarifying the GWAS methodology, and incorporating multi-environment statistical analyses. However, several methodological and interpretative concerns remain insufficiently addressed, particularly regarding experimental design limitations, QTL interpretation, and the depth of the discussion.

Overall, the manuscript has improved significantly but still requires minor revision before it can be considered for publication.

Line 66: Grammar issue “but require large-scale field validation”, should be “but requires large-scale field validation”

Line 80: Missing space “Goddard et al.[25]”, should be “Goddard et al. [25]”

Line 109–110: “including a wide range of agro-morphologically and genetically diverse accessions including…”. Repetition of “including”

Line 140: “anthesis[17,19,23]”, add space “anthesis [17,19,23]”

Line 142: “spikelet’s damaged”, Incorrect apostrophe, fix to “spikelets damaged”

Line 160: “disease pressure ,” remove space “disease pressure,”

Line 180: “picked up only SNPs”, replace it with “retained only SNPs”

Line 183: “we picked up the markers” replace it with “we retained markers”

Line 195: “Rwt4-B1 (Rmg1), Rwt4-D1 (Rmg1), earlier was Rwt4-1B / Rwt4-1D, must standardize naming everywhere

Line 200: “GWAS was performed by ‘R’ package”, better “GWAS was performed using the R package”

Line 200: “viz”, better, “namely”

Line 207: “Statistical explanation of each model is explained”, redundant, better write “Statistical details of each model are provided”

Line 215: “The common MTA in at least two GWAS models or in two environments were picked up”, better write “Common MTAs detected in at least two GWAS models or across two environments were retained”

Line 255 and 272: WB Index vs WB index.

Line 260: “The contrast results among environments”, better write “The contrasting results among environments”

Line 269: “reveals that WB index was highly heritable (> 50%)” better write “revealed that the WB index was highly heritable (>50%)”

Line 270: “Farthermore, the, overall 2 for WB”, typo + punctuation error “Furthermore, the overall H² for WB”

Line 275: “experienced high WB index (%)” add “a”, “experienced a high WB index (%)”

Line 292–294: “The approach ensures” better write “This approach ensures”.

Line 312 and 376: B-genome, sometimes B genome (Line 309, 373), unify.

Line 315-316: “while an additional 8% showed resistant reactions and 4% were classified as moderately resistant”, the word resistant is repeated, better write “while an additional 8% showed resistance and 4% were classified as moderately resistant”

Line 376: “These SNP markers identified from GWAS was then used”, replace was with were “These SNP markers identified from GWAS were then used”

Line 386: “with the first corresponds to”, better write “with the first corresponding to”

Reviewer #2: (No Response)

Reviewer #3: The authors have adequately addressed the major concerns raised in the previous round, and the manuscript has substantially improved in clarity, methodological transparency, and overall rigor. The experimental design, GWAS framework, and QTL delineation are now more clearly described, and the limitation of the study is appropriately acknowledged, including the role of these loci as a foundation for future validation. One minor point remains regarding the interpretation of GWAS results derived from maximum disease scores, which may favor environment-specific signals; it would be beneficial to more explicitly distinguish between loci with consistent effects across environments and those driven by specific conditions. With this minor clarification, I consider the manuscript suitable for publication.

**********

7. PLOS authors have the option to publish the peer review history of their article (what does this mean?). If published, this will include your full peer review and any attached files.

If you choose “no”, your identity will remain anonymous but your review may still be made public.

**Do you want your identity to be public for this peer review?** For information about this choice, including consent withdrawal, please see our Privacy Policy.

Reviewer #1: No

Reviewer #2: No

Reviewer #3: **Yes:** Samira El Hanafi

**********

# Editor report: Acceptance letter

**DOI:** 10.1371/journal.pone.0349201.r004

**Authors:** Wuletaw Tadesse

PONE-D-25-52203R1

PLOS One

Dear Dr. Singh,

I'm pleased to inform you that your manuscript has been deemed suitable for publication in PLOS One. Congratulations! Your manuscript is now being handed over to our production team.

At this stage, our production department will prepare your paper for publication. This includes ensuring the following:

* All references, tables, and figures are properly cited

* All relevant supporting information is included in the manuscript submission,

* There are no issues that prevent the paper from being properly typeset

You will receive further instructions from the production team, including instructions on how to review your proof when it is ready. Please keep in mind that we are working through a large volume of accepted articles, so please give us a few days to review your paper and let you know the next and final steps.

Lastly, if your institution or institutions have a press office, please let them know about your upcoming paper now to help maximize its impact. If they'll be preparing press materials, please inform our press team within the next 48 hours. Your manuscript will remain under strict press embargo until 2 pm Eastern Time on the date of publication. For more information, please contact onepress@plos.org.

You will receive an invoice from PLOS for your publication fee after your manuscript has reached the completed accept phase. If you receive an email requesting payment before acceptance or for any other service, this may be a phishing scheme. Learn how to identify phishing emails and protect your accounts at https://explore.plos.org/phishing.

If we can help with anything else, please email us at customercare@plos.org.

Thank you for submitting your work to PLOS ONE and supporting open access.

Kind regards,

PLOS ONE Editorial Office Staff

on behalf of

Dr. Wuletaw Tadesse

Academic Editor

PLOS One
